# Design and Characterization of Phosphatizing Coatings for Magnesium Implants

**DOI:** 10.1021/acsbiomaterials.5c01846

**Published:** 2026-02-13

**Authors:** Erdem Şahin, Francesco Paduano, Marco Tatullo, Roberta Ruggiero, Elisabetta Aiello, Rosa Maria Marano, Meltem Alp, Ahmed Şeref

**Affiliations:** † Department of Metallurgical and Materials Engineering, 52986 Muğla Sıtkı Koçman University, Mugla 48000, Turkey; ‡ Stem Cells and Medical Genetics Units, 695570 Tecnologica Research Institute and Marrelli Health, Crotone 88900, Italy; § Department of Translational Biomedicine and Neuroscience, School of Medicine, 9295 University of Bari “Aldo Moro”, Bari 70124, Italy

**Keywords:** AZ31 alloy, orthophosphoric acid, hydroxyethyl cellulose, cementitious coating, passivation

## Abstract

Magnesium alloys are promising biodegradable implant materials, but their rapid corrosion in physiological environments limits their clinical applications. This work is focused on the development of cementitious coatings inducing magnesium phosphate formation on magnesium AZ31 alloys. First, the alloy surfaces immersed in orthophosphoric acid (OPA) solutions with six additives of various functions (sodium chloride, magnesium chloride, calcium nitrate, magnesium nitrate, trisodium citrate, and hydroxyethyl cellulose (HEC)) were comparatively analyzed to understand the effect of solution chemistry on surface evolution. OPA solutions were also saturated with respect to magnesium ions, which effectively limited surface degradation. Sample mass and solution pH were monitored for 21 days, and depositions were characterized using SEM, EDX, and electrochemical methods to identify the surface composition and investigate its effectiveness against Mg degradation. In the next stage, alloy plates were dip-coated with the multicomponent suspension of the most effective composition (OPA, MgCl_2_, HEC, and Mg-saturated deionized water). The phase evolution of the dried samples in 3.5 wt % NaCl solution was monitored with regular gravimetric, pH, quantitative XRD, SEM, EDX, and electrochemical Tafel analyses. Samples passivated despite the high chlorine concentration, as initially formed newberyite crystals, were replaced by Mg oxychlorides, Mg phosphates, and Mg hydroxide in order, in response to the shift in solution pH from acidic to alkaline values that is driven by the dissolution and transformation of the alloy and coating phases. Thermally cross-linking HEC improved the stability of the coatings, which slightly retarded the degradation kinetics. In vitro cell culture tests validated the coated AZ31 as both being biocompatible and potentially bioactive. Thus, the phosphatizing coating approach offers a promising strategy for controlled biodegradation of magnesium implants in physiological environments.

## Graphical abstract

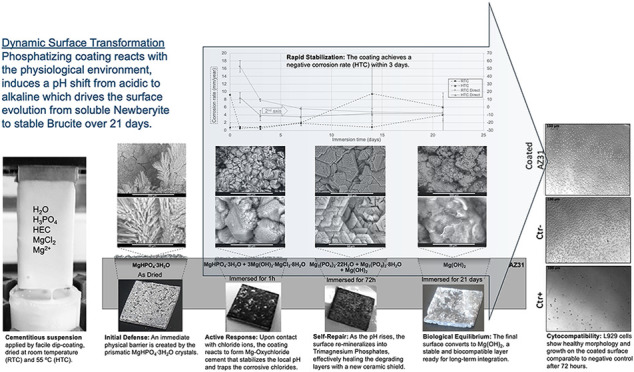

## 1 Introduction

Mg and its alloys are extensively used as temporary, degradable implants that can assist in augmentation and regeneration of bone provided that their mechanical properties are maintained for 4 to 16 weeks, depending on defect size, shape, and physiology.^1–5^ They are characterized by excellent biodegradability and biocompatibility, as well as suitable mechanical compatibility with human bone. Magnesium alloys containing high concentrations of elements, such as aluminum, zirconium, and rare earth metals, can cause tissue damage and inflammatory cascades. Therefore, not all compositions are considered suitable for resorbable medical implants.^3,6^ It has been demonstrated experimentally that the magnesium alloy AZ31, which has a comparatively low amount of aluminum (3%) and zinc (1%), is biocompatible.^7^ Despite these advantages, the extremely high corrosion rates of magnesium-based implants under physiological conditions containing chlorine significantly restrict their practical application. It can lead to rapid loss of mechanical integrity, excessive hydrogen evolution, and localized alkalization.^8–10^ Therefore, surface modification strategies are crucial for controlling deterioration while preserving mechanical integrity in vivo.^11^

Degradation mechanisms of magnesium surfaces in water have been elucidated in the excellent study by Song and Atrens.^12^ Briefly, an atom of magnesium dissolves in water to give out 2 electrons that are used to hydrolyze water into hydroxide and proton ions according to reactions 1–2. As a result, hydrogen gas evolves from the dissolving surfaces, and the hydroxides accumulate in water to increase the pH (3). Thus, degradation of the magnesium surface gradually makes the solution alkaline, which in turn induces the formation of Mg­(OH)_2_. Mg hydroxides are typically stable in aqueous solutions without chlorine ions so that they significantly reduce the degradation of the alloy.^13^ However, chemical species in the solution (e.g., chlorine or phosphate ions) can disturb their stability as such another magnesium compound becomes more stable in expense of the hydroxide phase according to reactions 4 and 5.^14,15^ In fact, the fast corrosion of Mg alloys under physiological conditions of pH 7.4 and 0.9% NaCl concentration is due to the rapid conversion of its surface into highly soluble Mg oxychlorides.^16^

1 $$\mathrm{Mg}\to {\mathrm{Mg}}^{++}+{2e}^{-}$$

2 $$H_{2}O\to {\mathrm{OH}}^{-}+H^{+}$$

3 $$2H^{+}+2e^{-}\to H_{2}$$

4 $$3\mathrm{Mg}{\left(\mathrm{OH}\right)}_{2}+\mathrm{MgC}l_{2}\cdot 6H_{2}O+2H_{2}O\to 3\mathrm{Mg}{\left(\mathrm{OH}\right)}_{2}\cdot \mathrm{Mg}{\mathrm{Cl}}_{2}\cdot 8H_{2}O$$

5 $$\mathrm{Mg}{\left(\mathrm{OH}\right)}_{2}+H_{3}{\mathrm{PO}}_{4}+H_{2}O\to \mathrm{MgHP}O_{4}\cdot 3H_{2}O$$

The latter reactions are exploited in magnesium oxychloride (Sorel) and magnesium phosphate cements (MPC) that have been used extensively in various industries as inorganic particulate binder materials. MPC’s are also effective bone cement compositions with similar mechanical properties, setting times, biocompatibilities, and bioresorbabilities to brushite calcium phosphate cements, which are used extensively in orthopedics.^17–21^ Their compatibility and adhesion to magnesium make utilization of this cement type as magnesium coatings an intuitive choice. This approach has been realized indirectly by the use of conversion baths to deposit highly stable and corrosion-resistant MPC products newberyite, farringtonite, and struvite on AZ31, depending on the bath chemistry.^22–26^ Despite their effectiveness in the short term, these magnesium phosphate depositions were reported to degrade after 24 h in saline solutions. In fact, cement products newberyite and struvite are reported to undergo a unique volume degradation mechanism in vivo even in compact, nonporous form.^21^ Therefore, a detailed investigation of their degradation and phase evolution in physiological fluids is necessary for understanding and optimizing coating performance. In parallel with this objective, the present study is built on the hypothesis that magnesium phosphate degradation can be directed as a self-passivation mechanism with tailored solution chemistry. More specifically, phosphate suspensions deposited on magnesium alloys can utilize the reactivity of the magnesium ions leaching out from the alloys to induce dynamic solution chemistry and a cascade of cement reactions, favoring the formation of passivating phases in aqueous salt solutions such as physiological fluids. Furthermore, with the right biochemical surface composition, such a cementitious coating may render typically biocompatible Mg alloys into bioactive surfaces due to its capacity to release biomolecules and biomimetic crystallization. In our recent study, we have demonstrated that even scarcely soluble calcium phosphate suspensions could passivate AZ31 in salt solution as a physical barrier by slowing down initial corrosion.^22^ Specifically, we focused on the relatively soluble AZ31 alloy to evaluate the efficacy of the proposed treatment.

This proof of concept study demonstrates the feasibility of an alternative approach to conversion baths, namely, phosphatizing cementitious coatings to provide sustained dissolution of phosphates and additives in the aqueous immersion medium and thus enabling further control on the implant stability by time-dependent phase transformations that are induced by the initial coating composition, alloy degradation kinetics, and the resulting solution chemistry.^27^ To that end, an aqueous orthophosphoric acid suspension was first designed, then coated on AZ31 as the initial surface makeup to investigate the resulting chemical reactions in the alloy-solution interface without any biological moieties. The coating was designed according to a comparative analysis of the AZ31 surfaces converted in OPA solutions with various additives by monitoring the deposition rates and composition, solution pH, and the degradation rates of the converted surfaces. AZ31 alloy was selected for its reactivity and mechanical compatibility with cortical bone.^28^ Although aluminum content is a potential concern, recent studies demonstrate that surface modifications effectively mitigate ion release, ensuring biocompatibility.^29–31^

## 2 Materials and Methods

Magnesium alloy plates were manufactured at Chongqing University by hot rolling at 340 °C to a thickness of 1 mm, followed by air cooling. The surface microstructure of the as-rolled plates was analyzed in our previous study.^13^ They were manually cut into square samples of 15 × 15 mm dimensions and cleaned by ultrasonication in deionized water and ethanol baths prior to immersion in aqueous solutions of OPA.

### 2.1 Immersion of Alloy Plates in OPA Solutions

All solutions comprised of 5 mL of 85% OPA in 45 mL of deionized water, and one of the functional adjuvants in the following amounts in addition to deionized water: 1 g of HEC (Sigma-Aldrich, CAS: 9004–62–0), 1.5 g of NaCl, 1.5 g of Mg chloride hexahydrate (MgCl_2_·6H_2_O), 1.5 g of Magnesium nitrate hexahydrate (Mg­(NO_3_)_2_·6H_2_O, Sigma-Aldrich CAS: 13446–18–9), 1.5 g of Ca nitrate tetrahydrate (Ca­(NO_3_)_2_·4H_2_O) and 1.47 g (0.1 M) trisodium citrate dihydrate (Na_3_C_6_H_5_O_7_·2H_2_O, Isolab chemicals, CAS: 6132–04–3). They were initially homogenized by an ultrasound probe at 300 W for 10 min and mixed by a magnetic stirrer for 3 h to saturate the solution and mix the residue phases. Alloy samples were immersed statically in the solutions in Falcon tubes for 21 days and analyzed at specific intervals (1, 3, 7, 14, 21 days) by gravimetry to account for the variations in their weights. Also, the solution pH was monitored at the same intervals using a glass pH electrode (Hanna Edge HI-2002). A parallel set of solutions were also added to 1 g of pure Mg strips that were chipped from a Mg ingot with a driller to investigate the effect of Mg saturation on the surface and solution chemistry.

### 2.2 Coating Preparation and Application

Concentrated suspensions of the reagents were prepared with higher solid loading, applied to AZ31 alloy plates by dip coating, and dried for deposition of stable multicomponent coatings. They comprised 85% of 85% OPA solution (8 mL, 2.37 M), magnesium chloride hexahydrate (5 g, 0.49 M), 25 mL of 4% hydroxyethyl cellulose, and 17 mL of magnesium-saturated water (total Mg added was 1.6 g). They were initially homogenized by an ultrasound probe at 300 W for 10 min and mixed by a magnetic stirrer for 24 h to ensure well mixing of the suspensions. The prepared coatings were applied by immersing AZ31 strips with approximate dimensions of 50 × 20 × 1 mm. All samples underwent an initial washing process. One side of the strips was coated with cellulose acetate tape to keep it conductive for corrosion tests after the coating. The dipping rate during immersion was set to a speed of 45 mm/min to ensure sufficient reaction time. Each dipping-withdrawal cycle lasted approximately 2 min, followed by room temperature drying using a high-speed fan for 3 min. After two drying cycles between the three immersion cycles, the coating process was completed in approximately 12 min. The coatings removed after the final immersion were placed in a ventilated environment at room temperature or kept in an oven at 55 °C for 72 h. Subsequently, they were immersed in a saline solution for extended periods of time to validate their barrier performance and characterize the evolution of the reacting surface.

### 2.3 Extended Immersion Tests

Dried coatings were immersed in separate polymeric mesh teabags in a 2 L volume of 3.5% NaCl solution for various periods of time for the extended corrosion tests. Initially, a coated strip was directly subjected to a corrosion test without prior immersion in the solution. Other samples were immersed in the salt solution for 1-, 3-, 7-, 14-, and 21-day periods, followed by subjecting them to the same corrosion test. Additionally, their corrosion rates were determined by directly measuring mass loss according to the ASTM G1–03 standard at the end of the immersion periods. Furthermore, the pH change of the immersion solution was monitored throughout the 21-day immersion. Gravimetry data were converted to corrosion rates according to the following equation:

$$c\mathrm{orrosion}\;\mathrm{rate}\;\left(\mathrm{mm}/\mathrm{year}\right)=\left(87,600\;\mathrm{mm}/\mathrm{year}*\mathrm{mass}\;\mathrm{loss}\;\left(g\right)\right)/\left(\mathrm{surface}\;\mathrm{area}\;\left({\mathrm{cm}}^{2}\right)*\mathrm{immersion}\;\mathrm{period}\;\left(\mathrm{hours}\right)*\mathrm{density}\;\left(g/{\mathrm{cm}}^{3}\right)\right)$$

### 2.4 Electrochemical Corrosion Tests

Dried samples were further characterized using a BioLogic electrochemical impedance spectrometer in a potential scan mode. A three-electrode setup was used, consisting of the sample housed in a sample holder with a 1 cm^2^ window as the working electrode, a platinum mesh electrode with a surface area of 1 cm^2^ as the counter electrode, and a saturated calomel electrode as the reference electrode. Initially, the open-circuit potential of the setup was determined upon immersion of the electrodes in 3.5% NaCl solution for a period of 15 min, which allowed the surface to reach an equilibrium potential. Subsequently, a potential scan around the open-circuit potential, from −1.9 to −1 V, was applied to induce both reduction and oxidation to the surfaces for equal periods of time. Samples were removed, washed in deionized water, and dried at room temperature prior to further analyses. The polarization curves were analyzed by the Tafel method using a fixed sample surface area of 1 cm^2^, equivalent weight of 12.15 g, and sample density of 1.74 g/cm^3^.

### 2.5 Surface Analysis

Phase evolution of the surfaces immersed in OPA solutions and 3.5% NaCl solution was monitored by scanning electron microscopy in imaging and spectroscopy modes and quantitative X-ray diffraction analyses. Morphological analysis was performed by using a Philips XL-30S FEG scanning electron microscope. A secondary electron detector was used to capture micrographs at an accelerating voltage of 3.00 kV and a wedge distance of 10 mm. An energy-dispersive X-ray detector was used for the elemental analysis of the sample surface. XRD analyses were conducted by using a Philips X’Pert Pro powder diffractometer with Cu K_a_ radiation at a generator voltage of 45 kV and a tube current of 40 mA. All XRD patterns were obtained at a scan step size of 0.05 and 4 s per step. The Rietveld refinement method was employed for the quantitative XRD analysis. Profex software from Doebelin.org and XRD references from the Crystallography Open Database were used for phase identification and quantification. The cross sections of coated and dried alloy plates were examined under an optical microscope (Nikon Eclipse) at 10× magnification. The sides of the coated plates were ground dry with fine grinding paper before they were mounted on a styrofoam support perpendicularly.

### 2.6 Biological Characterization

The in vitro cell toxicities of coated AZ31 and ZX31 alloy plates have been determined at Ege University Materials Research Laboratories (MATAL), İzmir Turkey. Samples with dimensions of 15 × 15 mm were extracted in cell culture medium and serum for 24 h at 37 °C, and then diluted at various ratios to incubate human-derived SaOS-2 cells (ATCC HTB-85). MTT tests were conducted according to ISO 10993–5 standard, and absorbances were measured using a spectrophotometer (at 570 nm) after 24 and 72 h of incubation at 37 °C with 5.0% CO_2_ and 95% humidity. The cell culture medium consisted of high-glucose DMEM (Capricorn CP 40–1309), 10% fetal bovine serum (FBS, A0500–3010, Cegrogen Biotech, Germany), 0.5% Gentamicin 10 mg/mL (A2712 Merck, Germany), and sodium piruvate 100 mM (L0473 Merck, Germany). Cell culture containing 1% dimethyl sulfoxide (DMSO) was used as the negative control, and cell culture without alloy plates was used as the positive control.

Additionally, the biocompatibility of the samples was evaluated using L929 (NCTC clone 929, ATCC CCL-1) cells in accordance with ISO 10993–5. The AZ31 samples were tested by the indirect method of the cytotoxicity assay. The L929 cells were cultured until they reached 80% confluence and then trypsinized and seeded at a density of 3 × 10^3^ cells/150 μL within 96-well plates. To obtain the extract medium for cell treatment, the samples were immersed in Dulbecco Modified Eagle’s (DMEM) supplemented with 10% FBS following a modified procedure of the ISO 10993–12 as proposed by Fischer et al., and later validated by Ruggiero et al.^30^ This modified approach involved a 10-fold increase in extraction volume, resulting in a sample weight-to-extraction volume ratio of 0.2 g/10 mL. Then, the samples immersed in the extract media were incubated for 72 h at 37° according to ISO 10993–12. After this time, the extracts have been collected and sterilized through a filter of 0.2 μm. 24 h after seeding, the cells were treated with 100 μL/well of AZ31 extracts and incubated for 1, 3, and 7 days. A 10% DMSO solution and DMEM were employed as positive and negative controls, respectively. At each designed time point, the cells were first observed under an optical microscope, and then 100 μL of 1× PrestoBlue solution was added to each well and incubated for 4 h, allowing viable cells to metabolize resazurin into resofurin.

The murine fibroblast cell line L929 (NCTC clone 929, ATCC CCL-1) and the human osteosarcoma cell line Saos-2 (ATCC HTB-85) were purchased from the American Type Culture Collection (ATCC, Manassas, VA). The L929 cells are an established line derived from mouse (*Mus musculus*) subcutaneous connective tissue, while Saos-2 cells are an established line derived from human osteosarcoma. Since this study utilized exclusively commercially available, immortalized cell lines and did not involve the use of live animals or the isolation of primary cells from human patients, specific institutional ethics committee approval and patient informed consent were not required.

### 2.7 Statistical Analysis

Biological and corrosion tests were repeated three times with different samples. Test results were reported in terms of 95% confidence intervals determined according to Student’s *t*-distribution. A statistical comparison was performed between the biological test groups and the controls by Student’s *t* test using the GraphPad Prism 6 software (GraphPad Software, Inc., San Diego, CA).

## 3 Results and Discussion

In our preliminary studies, 85% orthophosphoric acid (14.615 M) was used as the phosphatizing precursor, which, due to its high concentration resulted in a highly acidic solution that dissolved multiple immersed samples in a short time with vigorous hydrogen gas evolution. Upon saturation with respect to Mg, the solution became a viscous sol that was stable under ambient conditions. Subsequently, immersion of AZ31 plates in diluted OPA solutions (1.5 M) with various additives was monitored by gravimetry and a pH probe.

### 3.1 Deposition on AZ31 Plates Immersed in Various OPA Solutions

Since orthophosphoric acid provides the most acidic corrosion environment of all phosphates, the samples kept in its single- and double-component solutions had small and irregular surfaces upon extraction after 21 days. For this reason, some tests had to be repeated to obtain bulky samples. The sample kept in a 1.5 M OPA solution (DIW), seen in Figure 1C, is a small residue recovered from such samples. The crystal formations seen are high definition prisms that are characteristic of a newberyite (MgHPO_4_·3H_2_O) phase that forms spontaneously in Mg/PO_4_ systems under acidic conditions. EDX elemental analysis results in the Supporting Information (Table S1) confirm newberyite stoichiometry that has also been confirmed by XRD analysis as presented below.

**Figure 1:**
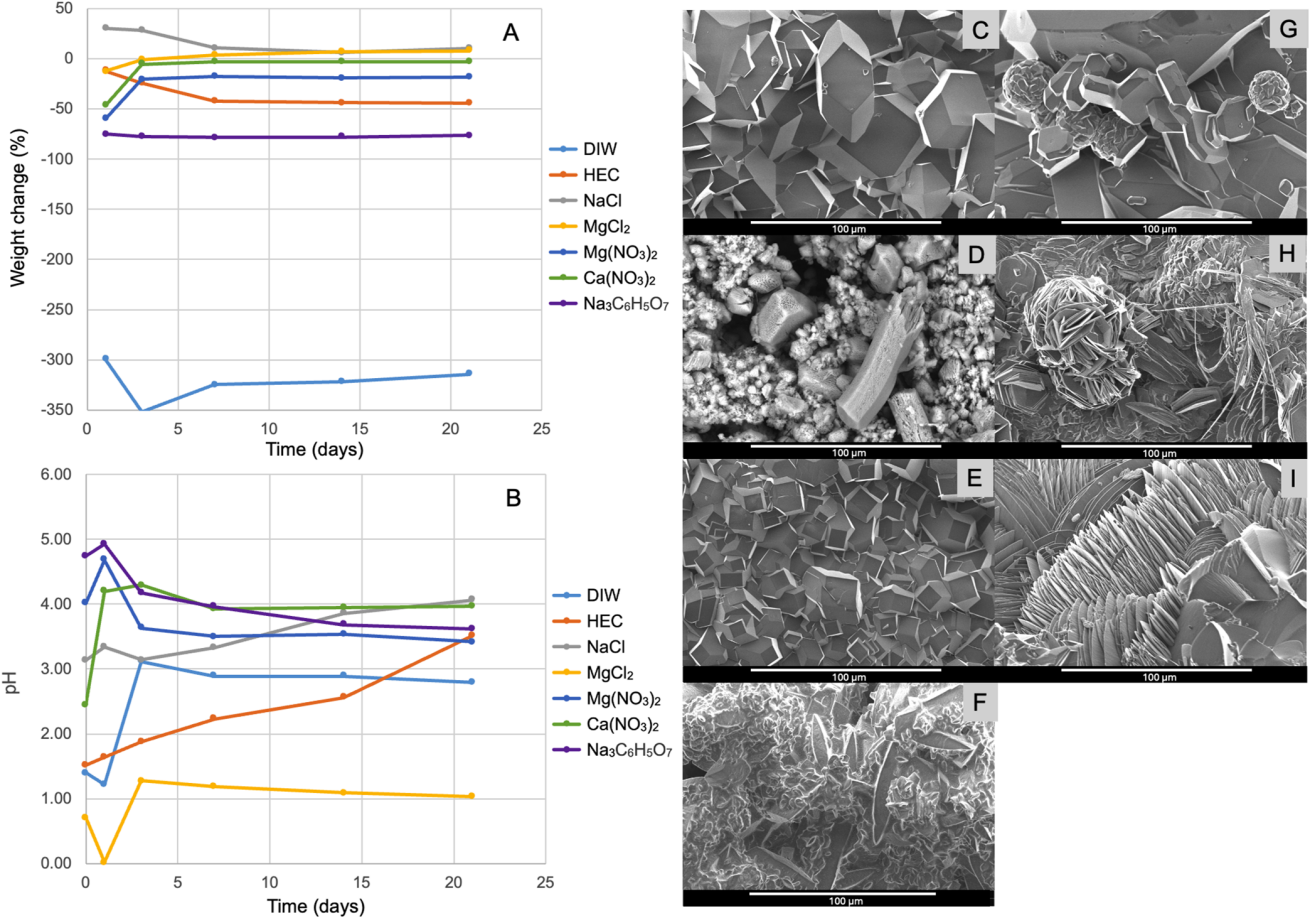
Variations in sample weight (A) and solution pH (B) with time upon immersion of the AZ31 plate into 1.5 M OPA solutions with various additives. The crystal morphologies obtained at the end of the immersion period are presented in the SEM micrographs for DIW (C), 2 wt % HEC (D), 3.5 wt % NaCl (E), 3.5 wt % MgCl_2_ (F), 3 wt % Mg­(NO_3_)_2_ (G), 3 wt % Ca­(NO_3_)_2_ (H), and 0.1 M trisodium citrate (I).

HEC was added to the solution of the OPA as a hydrogel with viscosity tunable according to its concentration and cross-linking extent. It generally slowed down the deposition but not the initial degradation that is expected to reduce and increase the pH, respectively. The former effect is related to its high viscosity, as indicated by the macroscopic views of the OPA/HEC solution (Figure S1 in the Supporting Information) wherein the alloy plate stays suspended in the viscous solution. High viscosity due to the entrapment of water by the hydrogel network is seen to slow the mass transport to the surface significantly so that surface reactions are limited by a diffusion barrier. Therefore, HEC functions to reduce the rate of degradation in such aggressive solutions and also can improve the mechanical stability of the coating. Its viscoelasticity and dynamical rheological properties have been optimized by a cross-linking heat treatment at 55 °C, as previously shown to induce stiffening of the thermoresponsive hydrogel.^27^ The microstructure of the surface in HEC containing the OPA solution is made of more elongated macro newberyite crystals that are embedded in a packed matrix of microcrystals (Figure 1D, Table S2 in the Supporting Information). The macro crystals seem to be corroded by the highly acidic medium after 21 days, and there is considerable porosity within and between them. Additions of HEC together with Mg saturation are expected to increase the effectiveness of solutions with OPA due to their retardation of Mg corrosion, as seen in the next section.

Chloride salts were added to the OPA solutions for activation of the alloy surface by the pitting corrosion mechanism commonly attributed to chlorine ions. Conversely, NaCl addition resulted in a much slower rate of alloy degradation and some extra deposition compared to the OPA solution (DIW). Variation of the pH shows one of the possible effects leading to this change. In NaCl-containing solution, pH starts above 3 and gradually reaches the highest value of 4. This is attributed to magnesium oxychloride precipitation on the surface that binds hydroxides with chlorides, which is another phase transformation favoring a rise in pH. Toward the end of immersion, the proton concentration reduced to about 1/100 of that in DIW, which apparently slowed down the alloy dissolution. Since Mg or MgO rather than Mg­(OH)_2_ are stable on AZ31 surface at the acidic pH levels of OPA solutions, chlorine ions that are known to penetrate Mg hydroxide in a pitting fashion do not promote degradation in the presence of OPA. The morphology seen in Figure 1E is again well-defined prismatic newberyite crystals covering the alloy surface compactly. EDX analysis results in Table S3 and detailed micrographs in Figure S2 indicate that newberyite and MgO are both present on the surface.

The high initial concentration of Mg ions in the MgCl_2_/OPA solution also inhibited surface dissolution and resulted in a very low pH of around 1. The similarly high deposition rate to NaCl solution indicates that Mg oxychloride formation is favored, whereas Mg dissolution is strongly inhibited. Similar newberyite composition is detected but with an irregular morphology consisting of crystals with worn-out corners under these highly acidic conditions (Figure 1F). Detailed micrographs in Figure S3 and EDX analysis given in Table S4 show a mixed morphology with parts of surfaces covered with needle-like crystals, characteristic of oxychloride crystals. The spectroscopy results show newberyite stoichiometry together with Cl, indicating a mixture of magnesium phosphate and oxychloride crystals.

Nitrate salts were added to the OPA solutions to prevent H_2_ bubble formation in the coating since they consume protons and convert to nitrites.^32^ Mg­(NO_3_)_2_ solution induced the formation of mostly newberyite (Figure 1G), while struvite (MgNH_4_PO_4_·6H_2_O) was also seen in some areas, as shown in the detailed micrographs and EDX analysis results (Figure S4 and Table S5). The elemental composition is obtained from a surface region consisting of three layers. The base layer is composed of MgO, the middle layer is struvite, and the top layer is newberyite according to their atomic ratios. Their micrographs show that the top layer is made of round lumps that are also made of round crystals in the microscale (Figure 1G). The pH fluctuation between 4 and 5 indicates the initial formation of newberyite, then its degradation and struvite formation, then newberyite back at the top.

Substitution of Mg nitrate with calcium nitrate resulted in depositions that contain elemental compositions close to dicalcium phosphate dihydrate, brushite (CaHPO_4_·2H_2_O), and newberyite (MgHPO_4_·3H_2_O) stoichiometries. EDX analysis in Table S6 and micrographs in Figures 1H and S5 show that these phases are found in a mixed state. A microcomposite layer is formed by their simultaneous formation in the solution with a suitable pH favoring the formation of both phases that are known as acidic calcium and magnesium phosphates, respectively.^13,33^ Figure 1H mostly shows the tabular crystals of newberyite and their flocculations. The fractured surface of this layer is exposed in the micrographs in Figure S5 as a dense ceramic structure consisting of overlapped layers that may provide effective barriers to degradation of the alloy surface.

Citrate salt is added to the OPA solution as a chelating agent for Mg ions and surface active sites to limit the dissolution and crystallization rates. Elongated prisms found in previous samples were also present in the citrate/OPA solution, together with the worn-out plates of newberyite observed in highly acidic solutions. Compositional analysis given in the Supporting Information indicates newberyite stoichiometry in various morphologies (Figure S6 and Table S7). The tabular crystals typically form as a result of citrate groups occupying growth sites on these plates.

### 3.2 Effect of Mg Saturation of the OPA Solutions on AZ31 Surface Composition

When the solution with the same OPA concentration is initially saturated with respect to Mg, the crystals on the surfaces of the immersed plates generally become smaller (Figure 2). While Mg saturation prevents dissolution, it also initially provides the driving force for the formation of Mg phosphate crystals on the surface. In this case, it is anticipated that the initial nucleation rate will be higher with a high number of resultant crystals. Coverage of the surface with depositing crystals is also expected to slow the dissolution of the alloy in later stages. Therefore, during long-term immersion, Mg conversion to Mg phosphate crystals remains limited, which causes the volume of the crystals to remain smaller as observed in Figure 2C compared to Figure 1C. A part of the pristine alloy surface is exposed in this micrograph, indicating the neutrality of the solution throughout the immersion process. Figure S7 also depicts the surface morphology of the AZ31 plate kept in a 1.5 M OPA solution for 21 days. According to the EDX analysis in Table S8, the smaller crystals also have newberyite stoichiometry.

**Figure 2:**
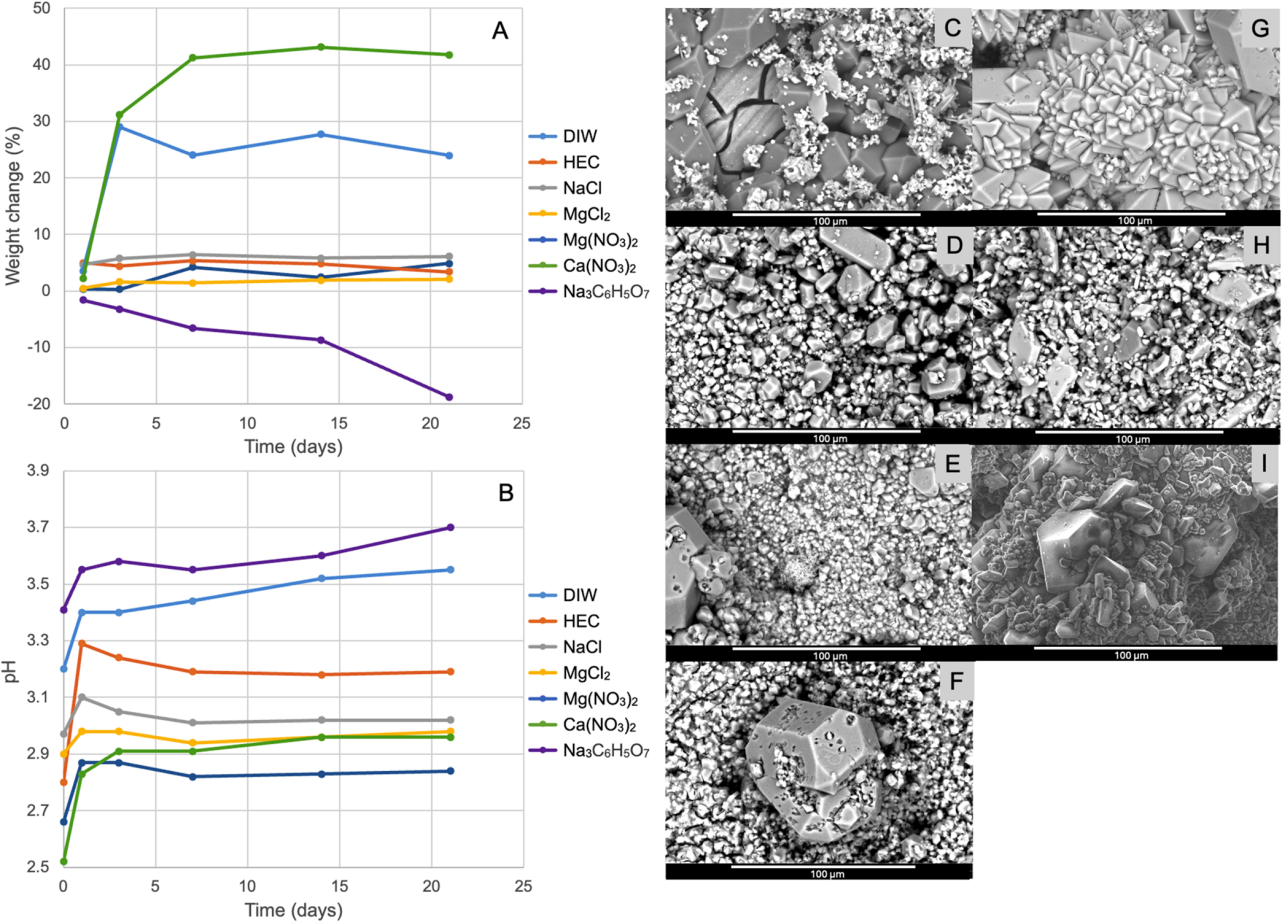
Variations in sample weight (A) and solution pH (B) with time upon immersion of the AZ31 plate into Mg-saturated 1.5 M OPA solutions with various additives. The crystal morphologies obtained at the end of the immersion period are presented in the SEM micrographs for DIW (C), 2 wt % HEC (D), 3.5 wt % NaCl (E), 3.5 wt % MgCl_2_ (F), 3 wt % Mg­(NO_3_)_2_ (G), 3 wt % Ca­(NO_3_)_2_ (H), and 0.1 M trisodium citrate (I).

As seen in Figure 2D, the surface morphology of the sample kept in the Mg-saturated HEC/OPA solution is a more compact structure compared to Figure 1D. Under these conditions, it is observed that newberyite crystals form coaxially, not longitudinally. Apparently, a different crystal formation has an effect on the density of the structure according to the EDX analysis results in the Supporting Information. Table S9 shows that the platy crystals have the stoichiometry of hydrated trimagnesium phosphate (Mg_3_(PO_4_)_2_). This phase covers the newberyite matrix and is seen to undergo degradation in response to the reduction in pH from pH 3.3 after the first day of immersion. Conversely, long newberyite crystals were observed in solutions without Mg saturation, where the pH continuously increased from 1.5 to above 3.5. Different phases and morphologies are attributed to variations in Mg/PO_4_ concentrations as well as pH of the solutions.

Mg saturation greatly influenced the conversion of the alloy surface immersed in chloride solutions. Both the pH profile and the surface morphology became more stable, which is attributed to a reduction in dissolution rates (Figure 2E,F). For both NaCl and MgCl_2_ solutions, the pH slightly varied in a narrow range around 3, whereas it was around 1 for MgCl_2_ solution without Mg saturation. Extra Mg may have resulted in supersaturation of a Mg chloride phase, precipitation of which seems to reduce the Mg concentration to a level that accelerates degradation of the alloy. Conversely, Mg saturation with NaCl inhibited the degradation of the alloy that is apparent from the constant pH level, while NaCl without Mg caused a gradual rise in pH to 4. This indicates that chlorine ions do not pit the Mg surface at the Mg ion concentration range of around 0.82 M, the amount added for Mg saturation. Table S10 shows that the matrix consists of fine newberyite crystals, whereas a few large newberyite, trimagnesium phosphate, and MgCl_2_ crystal formations are seen in Table S11, Figures S8, and S9. Generally, finer crystals indicate that a slow crystal growth at Mg and phosphate concentrations close to the saturation limit has occurred in the presence of Cl and Mg ions.

There was also a significant interaction of concentrated Mg and nitrate ions, as Mg saturation reversed the initial surface reactions from degradation to deposition, as seen in Figures 1A and 2A. Without initial Mg concentration, both Mg­(NO_3_)_2_ and Ca­(NO_3_)_2_ solutions induced strong dissolution of the surface that was also observed in ammonium dihydrogen phosphate solutions in our previous study.^34^ Nitrates consume hydrogen ions to convert to nitrites and eventually ammonia.^32^ Thus, they can prevent H_2_ gas bubble formation but also accelerate dissolution according to the Le Chatelier principle, since H_2_ is a product of Mg corrosion. Here, it is seen that Mg saturation alters this mechanism, presumably by limiting proton generation, in favor of a more stable and compact surface layer. Newberyite crystals seen in Figure 2G have formed a dense ceramic layer with the help of a high nucleation rate. These crystals form cauliflower-like polycrystals rather than typical isolated prisms, as shown in detail in Figure S10 and Table S12. Calcium nitrate substitution again induced the formation of brushite with newberyite that is a molecular analogue of each other in the Ca and Mg phosphate systems (Table S13). This mixed structure does not have the coherency of the newberyite matrix induced by Mg nitrate (Figures 2H and S11).

Mg saturation also reversed the surface activity in the presence of citrate ions, such that the initial solution pH changed from 4.8 for no-Mg to 3.4 for Mg saturation, resulting in gradual dissolution and an eventual rise in pH to 3.7 (Figure 2A,B). Citrate/OPA solution with Mg saturation showed the highest pH and weight loss among all others, similar to the behavior without Mg saturation (Figure 1A,B). Its synergistic effect with Mg is seen as a reduction in the dissolution rate as a rapid burst within the first day was seen without initial Mg saturation. The observed morphology changed as a result of stacked newberyite sheets to equiaxed crystals (Figure 2I).

Surface analysis of AZ31 plates immersed in the OPA solution was followed by corrosion tests of the extracted samples in 3.5 wt % NaCl solution. Applying a voltage sweep around the open-circuit potential value of the samples yielded different rates for each additive. The general order was Ca­(NO_3_)_2_ > DIW > Mg­(NO_3_)_2_ > trisodium citrate > HEC > NaCl > MgCl_2_. Three-component OPA solutions of the least corroding MgCl_2_, HEC, and trisodium citrate were prepared, and the same characterization tests were applied on AZ31 plates immersed for 21 days again. The weight loss and corrosion rates shown in Figure S12 and Table S14 were lowest for the surface immersed in the OPA, MgCl_2_, and HEC solution. Therefore, this composition was applied as a coating suspension on AZ31 plates, and its effect on surface degradation was assessed during the extended immersion test in salt solution. The other combinations of solutes containing OPA caused weight loss between 83 and 94% of the initial mass, while the solution containing OPA, HEC, and MgCl_2_ induced slight weight gain within the first week of immersion. The deposited layers after the 21-day immersion period all reduced the corrosion rate significantly compared to bare AZ31, with the lowest rate resulting from the deposit of the chosen solution (Table 1). The morphologies and elemental compositions of the deposits of these samples are presented in Figures S13–S16 and Tables S15–S18. When applied as a suspension coating and dried on the surface of the alloy, these compositions are expected to provide a partially converted alloy surface with cementitious chemical species that can further react in contact with physiological fluids. The initially present newberyite layer is expected to provide a barrier to chlorine attack and retard degradation of the alloy until the buildup of solution supersaturation and shift in pH toward alkaline levels that may provide further driving force for surface evolution.

**Table 1:** Comparison of Various OPA Solution Compositions in Terms of the Deposition Composition and Corrosion Rate

deposition solution	deposited phases	corrosion rate
DIW (no OPA)	Mg_6_Al_2_CO_3_(OH)_16_·4H_2_O _9_	4.443 ^22^
1.5 M OPA	MgHPO_4_·3H_2_O	0.113
1.5 M OPA + 2% HEC	MgHPO_4_·3H_2_O	N/A^a^
1.5 M OPA + 3% NaCl	MgHPO_4_·3H_2_O 5Mg(OH)_2_·MgCl_2_·8H_2_O	0.404
1.5 M OPA + 3% MgCl_2_	MgHPO_4_·3H_2_O 3Mg(OH)_2_·MgCl_2_·8H_2_O	N/A^a^
1.5 M OPA + 3% Ca(NO_3_)_2_	MgHPO_4_·3H_2_O, CaHPO_4_·2H_2_O MgNH_4_PO_4_·6H_2_O	N/A^a^
1.5 M OPA + 3% Mg(NO_3_)_2_	MgHPO_4_·3H_2_O	2.006
1.5 M OPA + 0.1 M trisodium citrate	MgHPO_4_·3H_2_O	1.163
**1.5 M OPA + 3% MgCl**_**2**_ **+ 2% HEC**	**MgHPO**_**4**_ **·3H**_**2**_ **O and Mg**_**3**_ **(PO**_**4**_ **)**_**2**_ **·8H**_**2**_ **O**	**0**.**127** ^b^
1.5 M OPA + 3% MgCl_2_ + 0.1 M trisodium citrate	MgHPO_4_·3H_2_O and 5Mg(OH)_2_·MgCl_2_·8H_2_O	0.173
1.5 M OPA + 3% Mg(NO_3_)_2_ + 0.1 M trisodium citrate	MgHPO_4_·3H_2_O	0.326
1.5 M OPA + 0.1 M trisodium citrate + 2% HEC	MgHPO_4_·3H_2_O	0.257
1.5 M OPA + 3% Mg(NO_3_)_2_ + 2% HEC	MgHPO_4_·3H_2_O	N/A^a^

a Samples too degraded for corrosion test.

b Most effective composition yielding the lowest corrosion rate.

### 3.3 Immersion of Coated AZ31 in 3.5 wt % NaCl Solution

Coatings were applied on AZ31 plates by simple dip-coating and dried under two different conditions: ventilation at room temperature and heating to 55 °C in an oven. Cross-linking of hydroxyethyl cellulose was induced by the latter according to a thermo-mechanical test showing this induction temperature from our previous study.^27^ Both sets of dried coatings were immersed in 2 L of 3.5 wt % NaCl solution for extended periods of time to simulate physiological conditions. The pH evolution of the immersion media is shown in Figure 3. The solution instantly became acidic upon immersion and dissolution of the OPA and newberyite phases deposited during the coating. Initial pH shifted from 4 to 5 in a few hours for both sets, which is attributed to multiple reactions, including the dissolution of Mg alloy surface and the precipitation of Mg chloride and Mg phosphate phases from the solution as detected in the XRD analyses. The coatings delaminated within the first day and exposed the alloy surface. Further dissolution of the coating is presumed to saturate the solution with phosphate ions that initiated a magnesium phosphate cement reaction according to the sigmoidal pH profiles exhibited by the RTC and HTC samples after and before the first day of immersion, respectively. The setting kinetics of inorganic cements have been accurately described as such sigmoidal functions of time by directly probing the cement paste thermally, mechanically, chemically, or electrochemically.^35–39^ Occurrence of the maximum setting rate within the first day for the HTC set coincides with the higher magnesium phosphate formation at day 1 obtained with XRD analyses. The late shift in pH from 5 to 9 for the RTC set also coincides with higher magnesium oxychloride formation at day 1 that is mostly replaced by magnesium phosphates at day 3. Dehua et al. have shown in their potentiometric titration study of the Mg oxychloride cement system that its setting curve stabilizes in the range pH = 5–7.5, reaching neutral values as the water molar ratio is increased.^40^ Thus RTC set first undergoes Mg oxychloride formation while the early formation of Mg phosphates on the HTC samples seems to bypass this mechanism by shifting the pH through Mg phosphate formation which is reported to occur in a wide pH range from 4 to 8.^41,42^ Although the underlying mechanism is not clear, it is assumed that cross-linking of HEC provides a faster dissolution/release of the phosphates initially incorporated to the coating. Moderately alkaline pH levels are reached toward the end of the immersion test due to the observed continuous phase evolution for both sets.

**Figure 3:**
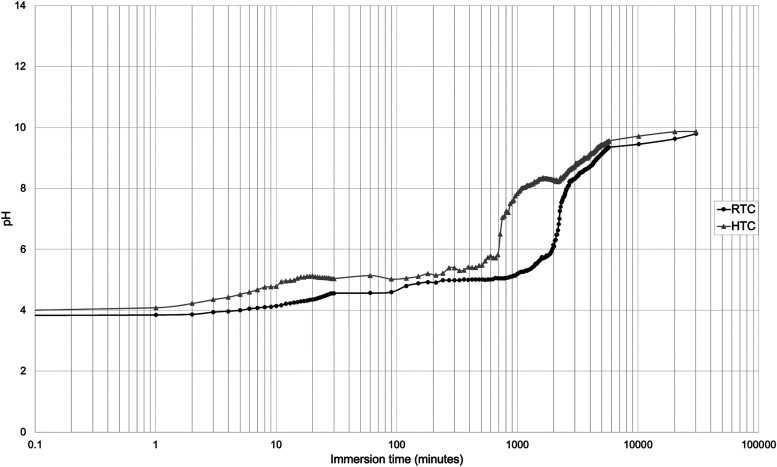
Variation of the immersion medium pH during the immersion of coated AZ31 plates of samples dried at room temperature and at 55 °C.

Weights of the coated samples were monitored at specific immersion intervals, which enabled direct calculation of the corrosion rate as a function of time according to ASTM G1–03. As seen in Figure 4, the directly measured corrosion rates started from around 50 mm/year for the room-temperature-dried (RTC) samples, whereas the corrosion rate on the first day was around 10 mm/year for the heat-treated (HTC) samples. These high values are partly due to the delamination of the coatings on the first day and partly due to the direct nature of the test that generally starts at higher values and ends at lower values compared with the electrochemical Tafel analysis results that are shown in the same figure as well. Directly measured rates quickly stabilized to a net negative rate for both sets of coated samples, with the HTC set starting to accumulate deposits after the third day. RTC samples reached that level by day 7 and stabilized to similar corrosion rates around −10 mm/year toward the end of immersion.

**Figure 4:**
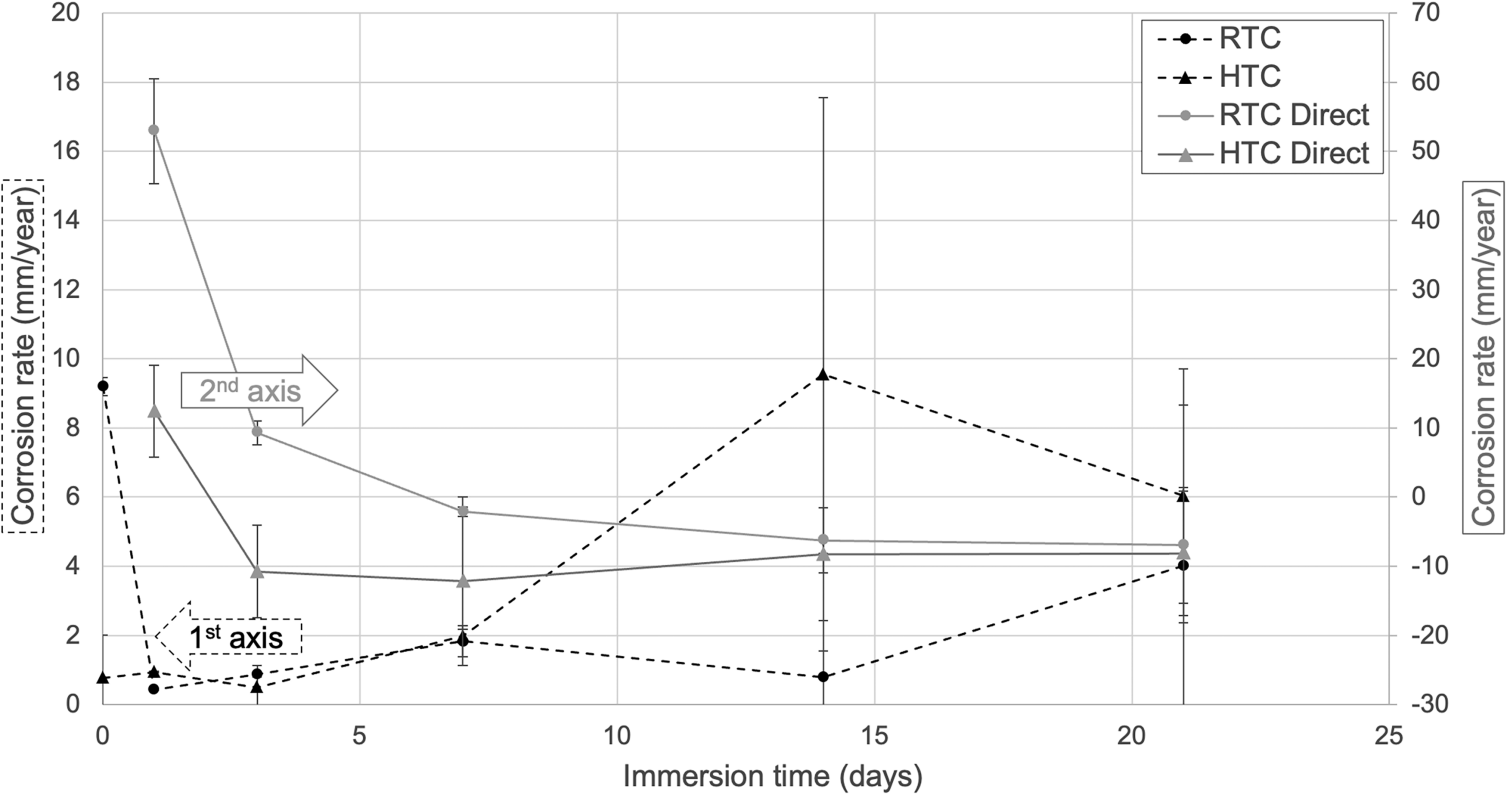
Corrosion rates of the coated AZ31 samples immersed for various periods of time in 3.5 wt % NaCl solutions.

The facility of cross-linked coatings for initial passivation was also observed in indirect Tafel analysis results obtained from voltage sweep tests. Tafel analysis is based on the extrapolation of the electron transfer rate toward the sample surface at the short time period of applied voltage to a year, hence it is prone to errors associated with surface changes occurring during that period, such as mechanical wear due to handling or dissolution due to concentration differences in the immersion media. Therefore, it is generally expected to observe higher corrosion rates using Tafel analysis compared to the direct weight measurement, as is the case here for longer periods of immersion time. The gradual direct measurement method, taking into account the whole history of surface conversion, is assumed to provide more accurate real-time data, considering the slowly evolving surface microstructure that is characteristic of cementitious coatings on Mg alloys. The parallel curves for the two sets match well until the last two data points taken at longest immersion times when the surfaces showed significant variations, including deep pits and cracks. The effect of such defects is accentuated especially at long periods of immersion that are averaged out in the direct measurement but not in the electrochemical tests. High concentrations of coating defects were seen to form during the dip coating process due to H_2_ gas evolution at the reacting interface that is thought to produce this variation in the corrosion rate in the long term. Simply bursting the H_2_ bubbles by a pressurized air gun was seen as an effective way to inhibit the formation of weak spots in the coating.

The thicknesses of the dried coatings were determined under an optical microscope. The composite images from three zoomings given in Figures S17 and S18 show that the average thicknesses of the two sets of coatings are similar, about 280 μm for RTC and 283 μm for HTC. As also seen in the macrograph insets in these figures, two layers have formed on the substrates after coating. There is a dark inner layer that is a highly coherent interphase on top of the alloy surface, presumably composed of newberyite. This interphase is a conversion coating that formed during the dipping and drying stages in contact with the reactive coating suspension. Its average thickness was measured as around 148 μm for RTC samples and 141 μm for HTC. The second bright layer that is about the same thickness as the inner layer is deposited upon drying and consists of unreacted salts of the suspension components. The bright prismatic crystals of these cementitious phases were physically embedded in the hydrogel matrix that adhered compactly to the inner layer. Still delamination was observed at some spots where the inner layer was exposed presumably due to microbubble formation as a result of H_2_ gas evolution during the wet coating stage. Less compact, flaky parts of the top layer were also observed for the same reason. Compaction during coating with the facility of a pressurized air gun minimized such defects and improved the binding of the two layers. The adhesion of the OPA-based coatings to the alloy surface was adequately high for handling and scratching due to the ionic bonding of the developed Mg phosphate interphase between the hydrogel suspension and the alloy surface. Drying and cross-linking of the coatings further stabilized them on the alloy, such that no erosion was observed upon manual impact.

In practice, cohesion of inorganic bone cements during handling and implantation by the surgeon is considered important, given their transient nature upon implantation.^33^ When mixed with water, typical bone cement forms a paste whose cohesiveness depends on the particle surface area and water volume. Under optimal conditions, it rapidly sets to a doughy consistency through an exponential rise in its elastic modulus and viscosity with time. In contrast, cementitious coatings are dried inorganic particles with surface area limited by the substrate surface area and deposition thickness. They are wetted and activated in contact with physiological fluids, so that they are expected to lose cohesion elasticity upon implantation. In fact, they are seen to delaminate from the surface, with debonding occurring through a combination of leaching and erosion mechanisms within the first day of immersion. Unlike conventional inert coatings, their bonding strength to the alloy substrate is significant only until implantation, after which they are desired to convert with the alloy surface.

### 3.4 Phase Evolution of the Coated Surfaces in 3.5 wt % NaCl Solution

Quantitative XRD analysis of the surfaces immersed for various periods clearly shows the phase evolution with immersion time. Figure 5 shows that the RTC samples, which made the salt solution acidic at the moment of immersion, were initially covered with the newberyite (MgHPO_4_·3H_2_O) phase. Within a few hours, the solution pH increased from 3.5 to 6, resulting in complete degradation of the newberyite phase within the first day. The reason for the rapid pH increase is that during this period, the coatings detached from the surface, exposing it, while the chloride ions broke down the underlying passive hydroxide phase. Within the first day, magnesium was released and oxychloride phases covered the surface. During the subsequent immersion process, the pH gradually increased from 6 to 9. In the long term, the oxychlorides dissolved, and the magnesium phosphates evolved from acidic to basic phases, making the solution alkaline. A similar phase evolution was observed for the HTC set, the main difference being the earlier phase transformations.

**Figure 5:**
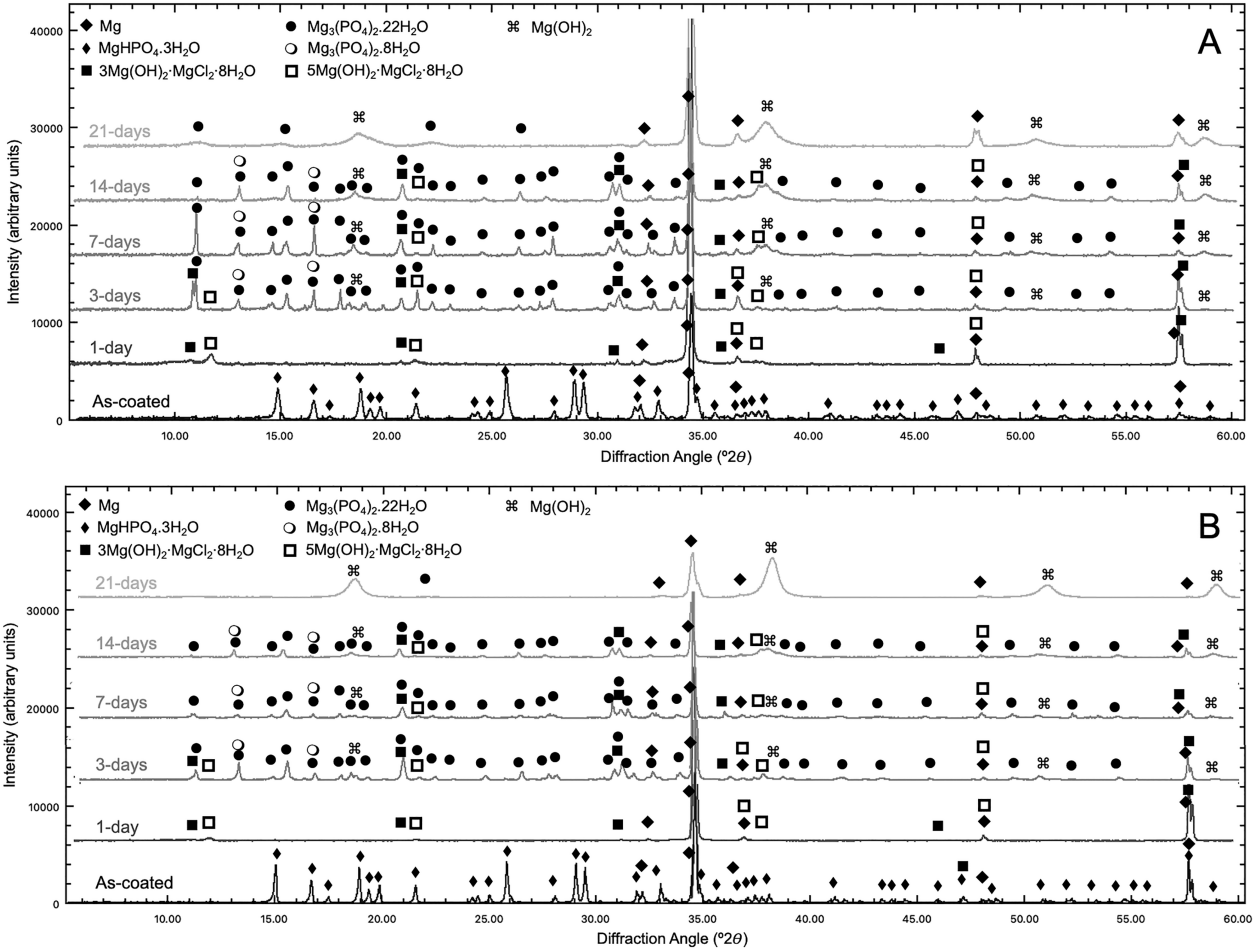
XRD phase analysis after various immersion times of samples dried at room temperature (A) and 55 °C (B).

Specific weight fractions of the stable phases on the surfaces were obtained by Reitveld analysis of the diffraction patterns. As seen in Figure 6 with surface analysis, the initial samples predominantly contained newberyite. The inset micrographs show the disintegration of newberyite crystals in a peeling fashion, upon corrosion testing in NaCl solution for about 30 min. This interesting strip morphology is a manifestation of the strong chlorine infiltration into Mg phosphate crystals as well as into Mg­(OH)_2_ that is well documented in the literature. Within the first day, this layer completely converted to magnesium oxychloride (5Mg­(OH)_2_·MgCl_2_·8H_2_O or 5–1–8, and 3Mg­(OH)_2_·MgCl_2_·8H_2_O or 3–1–8) phases. The driving force behind this sudden change is apparently a rapidly increasing pH due to chlorine attack. Uncoated alloy surface gave way to needle-like clusters of the same oxychloride phases, which are typically accompanied by pitting of the underlying substrate.^13^ As magnesium surface dissolved, pH continued to rise, which gradually degraded the 5–1–8 phase formed on the first day, which is known to be a metastable precursor for 3–1–8 formation in the cement literature. Accordingly, the 3–1–8 phase was seen to be more stable throughout the late stages. Magnesium was detected in large proportions in both samples, peaking on the first day of immersion and then gradually reducing to 5% by day 7. Sudden formation of magnesium phosphates was observed on the third day for both RTC and HTC samples (Figure 7). Supersaturation buildup upon exposure and dissolution of magnesium substrates as well as acidic phosphate induced the formation of trimagnesium phosphates, cattiite (Mg_3_(PO_4_)_2_·22H_2_O), and a small amount of bobierrite (Mg_3_(PO_4_)_2_·8H_2_O) phases, which further increased the pH. The microstructure at the end of the immersion test is similar to the as-coated morphology with brucite replacing magnesium phosphate crystals. These nanosized crystals, as indicated by the broad XRD peaks, seem to cover the entire surface as a thin layer that is expected to accentuate the diffraction from the underlying alloy. A relatively lower intensity of magnesium peaks from HTC samples was in parallel with the gravimetry data presented in Figure 4 as the lower direct corrosion rates indicated more deposition from the solution throughout the immersion period.

**Figure 6:**
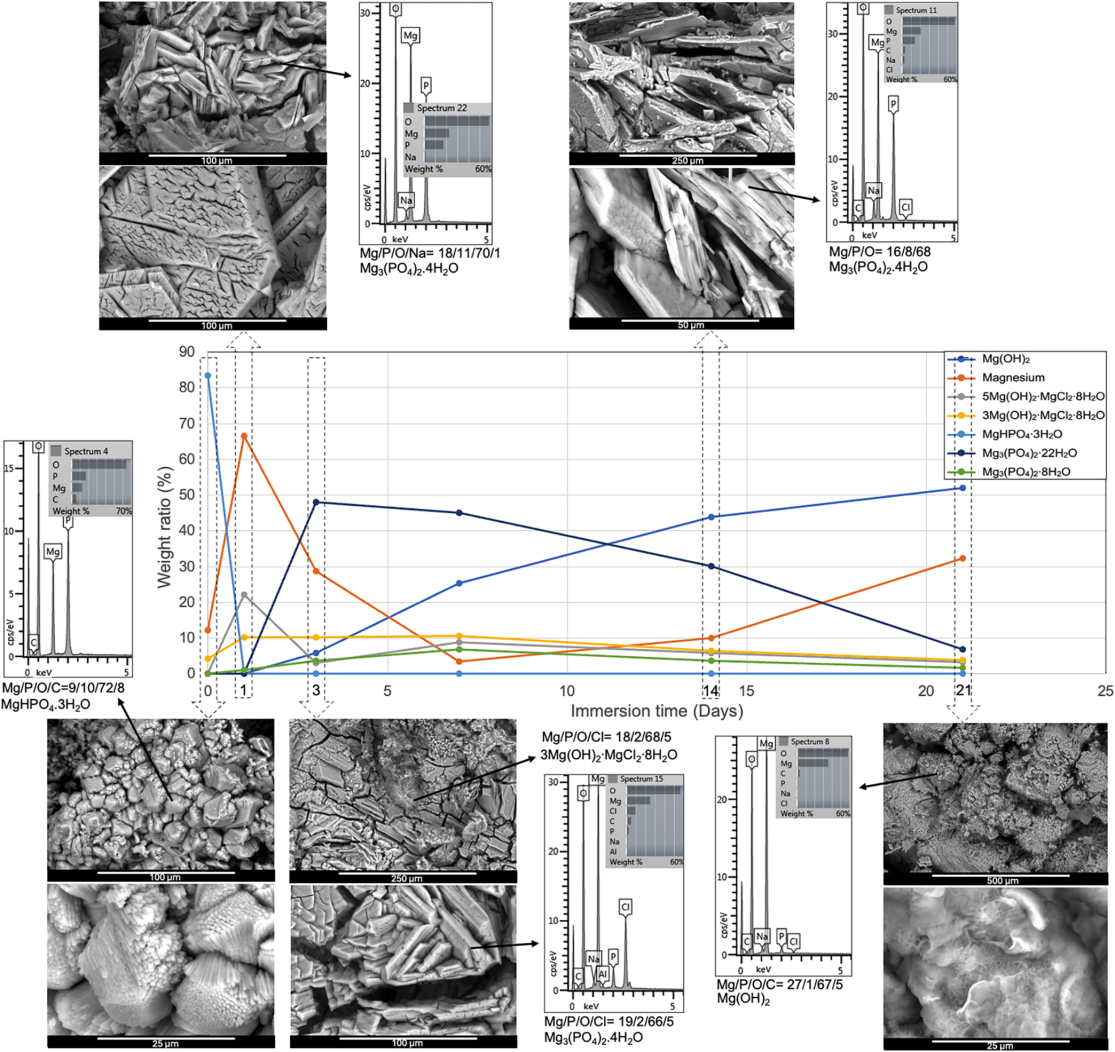
Evolution of the surface composition of room-temperature-coated AZ31 plates immersed in 3.5 wt % NaCl solution. Inset spectra were taken from the shown micrographs. Atomic percentages are presented quantitatively under the spectra with the most probable stoichiometry.

**Figure 7:**
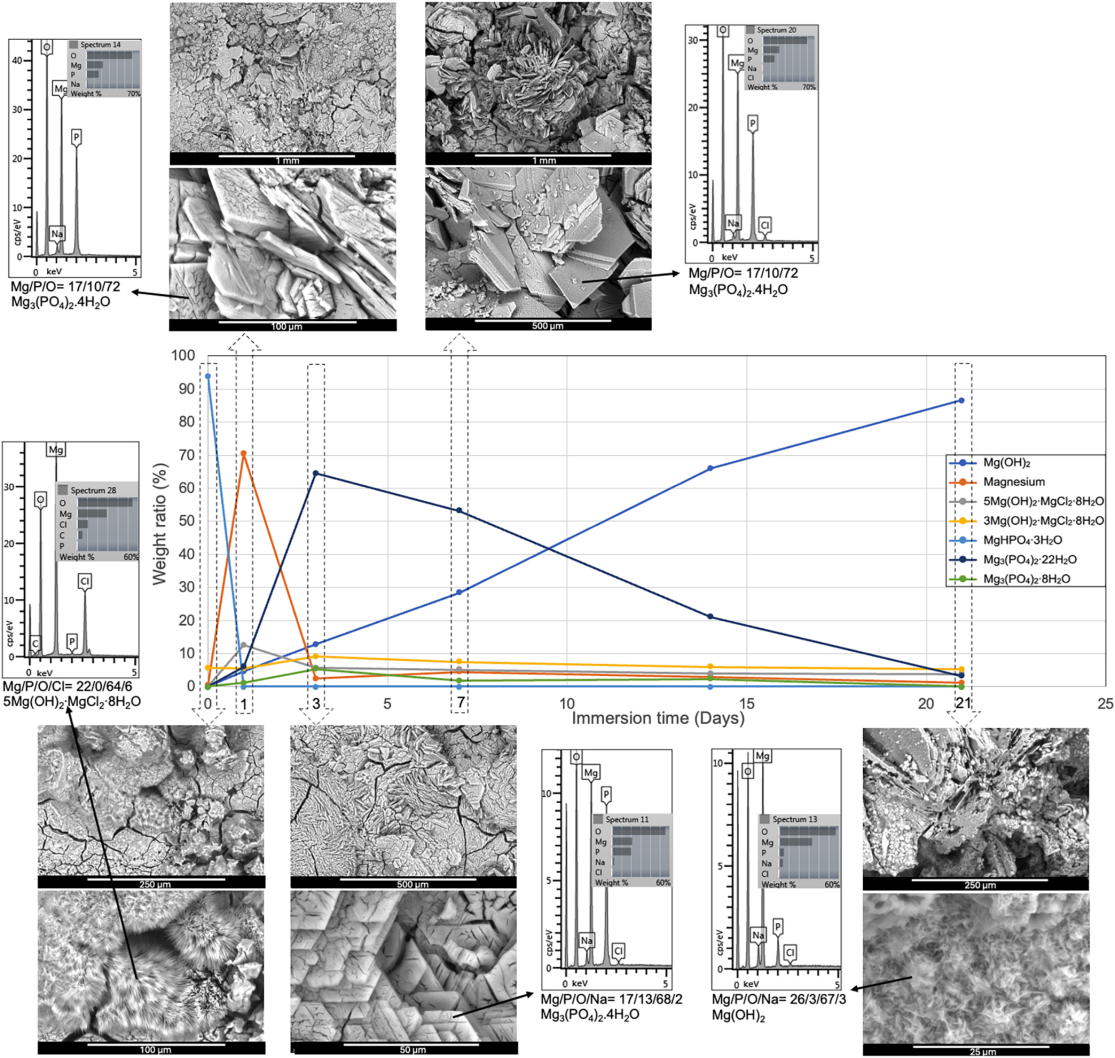
Evolution of the surface composition of heat-treated coatings on AZ31 plates immersed in 3.5 wt % NaCl solution. Inset spectra were taken from the shown micrographs. Atomic percentages are presented quantitatively under the spectra with the most probable stoichiometry.

The time-dependent morphological changes of the samples dried at 55 °C are shown in Figure 7. The morphology of the oxychloride phase along with the newberyite covering the initial sample surface is seen in the insets. Another interesting phase transformation is observed initially in the detailed micrograph, where the as-coated sample was again subject to chlorine attack in the corrosion test medium. Apparently, newberyite crystals started to decompose and transform into thin oxychloride (5–1–8) filaments during the test. The EDX analysis results that are given in more detail in Table S19 confirm that these formations contain chlorine close to the 5–1–8 stoichiometry. Similarly decomposing strips seen in Figure 6 seem to be the precursor of the oxychloride filaments that form by chlorine infiltration further into the crystal structure. Detection of Na and Cl in all spectra is assumed to be due to residues of dried solution after immersion tests. As seen in the micrographs in Figures 6 and 7, the surfaces previously covered with newberyite have worn down and converted to the 5–1–8 phase after 1 day of immersion in the salt solution. While new needle-like oxychloride clusters are observed in some areas after the first day, secondary phosphates that are known to be stable at neutral pH have started to form.^41^ The hexagonal formations on the surface on the third day are attributed to rapid crystal growth due to both increasing pH and high phosphate and magnesium concentrations, resulting in stacking of trimagnesium phosphate plates on top of each other. In parallel with the composition analysis, it is clear that this structure also began to degrade, as indicated by the dissolution pits on the crystal surfaces. By the 14th and 21st days, the prismatic crystals have eroded, and their surfaces were covered with the magnesium hydroxide phase. As a cumulative effect of phase formation and degradation, the surface has become increasingly indented, and the increased surface area has accelerated the degradation.

Analysis of the elemental compositions from EDX in conjunction with XRD results indicates that the trimagnesium phosphate precipitates on the surfaces are generally in a state of lower hydration compared to those at the bulk coating layer. Cattiite with the highest state of hydration is known to precipitate initially from neutral to alkaline solutions and at low temperatures.^43^ XRD results show, in parallel with the literature, that cattiite dominates the coating composition at day 3. According to the literature, solution pH evolution directly affects the solubility of trimagnesium phosphates, but there is no clear correlation for the order of precipitation with increasing pH due to its dependence on Mg^2+^ and PO_4_^3–^ concentrations as well as temperature. Precipitation studies clearly show that one of the hydrated trimagnesium phosphates rather than the anhydrate farringtonite precipitate from aqueous solutions. All trimagnesium phosphates transform to brucite above pH 10.^43,44^ It is also reported that trimagnesium phosphates initially precipitate as amorphous magnesium phosphate with a significant water content that transforms into cattiite, bobierrite, trimagnesium phosphate pentahydrate, and farringtonite with time and temperature.^45,46^ Hence, it is expected that the crystals at the surface in contact with the increasingly alkaline solution consist of different phases compared to the bulk, where transformation is assumed to be slower. The abundance of etch pits on these crystals confirms their transient composition at all immersion extents. The less hydrated Mg_3_(PO_4_)_2_·4–5H_2_O phases detected by EDX analysis, with a typical analysis depth of a few micrometers, apparently cover the surface around the etch pits that display comparable widths and depths. In comparison, the penetration depth of X-rays for the typical XRD analysis is expected to be an order of magnitude higher, yielding signals from the untransformed bulk of the crystals.

Detailed micrographs of both sets of samples at various immersion times presented in the Supporting Information show time-dependent formation and degradation of the mentioned alternative phases and morphologies (Figures S19, S22–S35, Table S19). According to the EDX data, there are significant differences in the stoichiometries of these phases. Their evolving Mg/Cl, Mg/PO_4_, and Mg/OH ratios from quantitative XRD analysis, shown in Figures S20 and S21, reflect the variation in the stabilities of these phases with the solution pH. It is generally seen for both sets that the precipitated hydroxide/magnesium ratio increased on the first day due to the rising pH, and then decreased with the formation of chloride phases. The continuously increasing pH and the degradation of intermediate phases provided the driving force for eventual brucite formation. The decrease in phosphorus ratios indicates that the degradation mechanism involves the acidic phosphate groups being drawn into the alkaline solution and the gradual change in the crystal structure to brucite. In both sets, the chlorine/magnesium ratio started to decrease from the third day and gradually reached a minimum level on the 21st day. The phosphate/magnesium ratio also changed in parallel with the chlorine ratio, except for the period between days 1 and 3, when secondary phosphate formation began with the decline of the 5–1–8 phase.

### 3.5 Biological Characterization of the Coated AZ31 Plates

The in vitro cytotoxicity test result of the coating obtained from the MTT assay is presented in Figure 8, as the percentage of viable cells relative to the negative control. The RTC coating applied to AZ31 was found to be safe at 1/10 and 1/5 dilution ratios at 24 and 72 h, while it showed a significant toxic effect at the highest concentration. In comparison, the uncoated alloys exhibited perfect biocompatibility under the same conditions. The coating slightly acidified the pH of the culture during the extraction process, therefore also accelerated the dissolution of the Mg alloy, particularly the toxic Al impurity. When the coating was applied to ZX31, another magnesium alloy with 3 wt % zinc, 1 wt % calcium, and no aluminum, it provided higher biocompatibility except for the most concentrated extract as seen in Figure 8B. Therefore, the toxicity of the extract of the coating applied on AZ31 at high concentrations can be partially attributed to the elevated Al concentrations.

**Figure 8:**
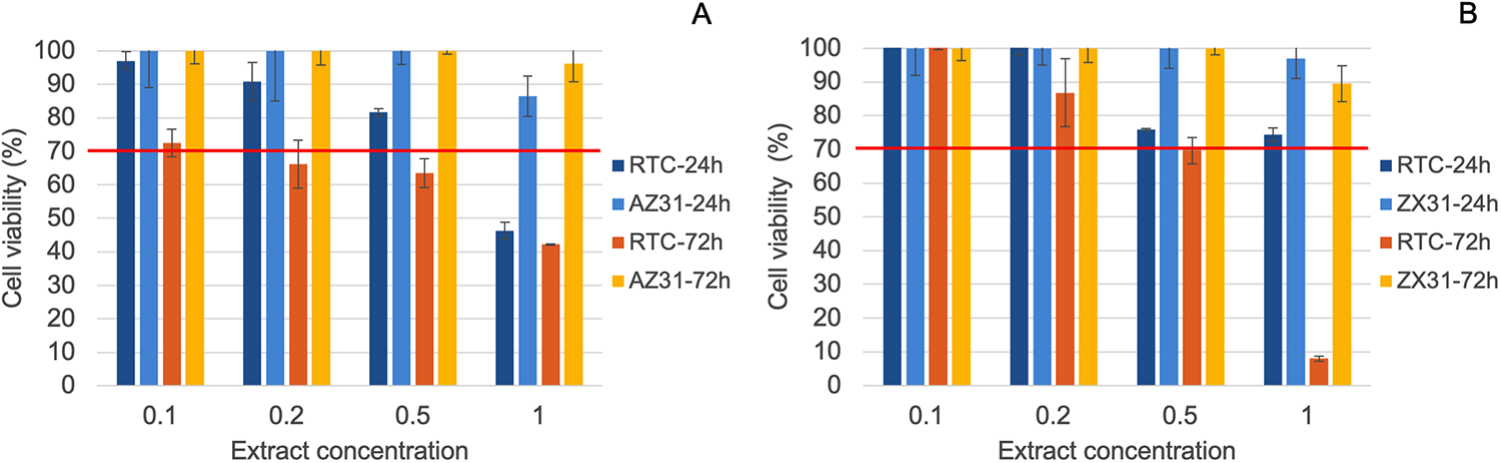
In vitro cytotoxicity test results for room-temperature-coated magnesium alloy samples obtained from the MTT assay using SaOS-2 cells: (A) Coating on AZ31 and the bare alloy, (B) coating on ZX31 and the bare alloy. The reference line indicates the standard safety limit for the test.

The most diluted extracts for both coated alloys provided maximum cell viability at both 24 and 72 h, and the 2-fold concentrated extracts showed similar biocompatibility. The 5-fold concentrated extracts also provided biocompatibility above the 70% threshold at both times. The most concentrated extract remained biocompatible at 24 h but showed high toxic effects at 72 h. Similar increases in toxicity over time were observed in other coatings containing HEC.^27^ This may be attributed to the gradual breakdown of the polymer structure to potentially toxic chemical groups. Regarding other additives, it is presumed that the addition of 3 wt % Mg chloride would not provide a significant difference in chlorine concentration from the physiological medium. As an alternative, it was observed that the addition of NaCl provided a milder pH that may increase cell viability indirectly through inhibition of Al dissolution. Hence, it is possible to make this coating safer by reducing its HEC concentration and acidity through NaCl replacement.

An alternate in vitro cytotoxicity test was performed on the L929 cell line using the Resazurin assay. The figure illustrates the different phases of the indirect cytotoxicity assay used to obtain the extracts (Figure 9A–C). Figure 9A shows a representative coated AZ31 sample at *t*_0_ (before immersion in the culture medium), while Figure 9B displays the extracts obtained after the extraction phase (72 h), which appear clear and transparent macroscopically, with no visible precipitates. Figure 9C depicts the coated AZ31 samples at *t*_1_ (72 h after immersion in the culture medium). The coated AZ31 samples show no visible signs of macroscopic degradation after the extraction phase. These findings suggest that the coating effectively mitigated the degradation process of the AZ31 samples, preserving their structural integrity.

**Figure 9:**
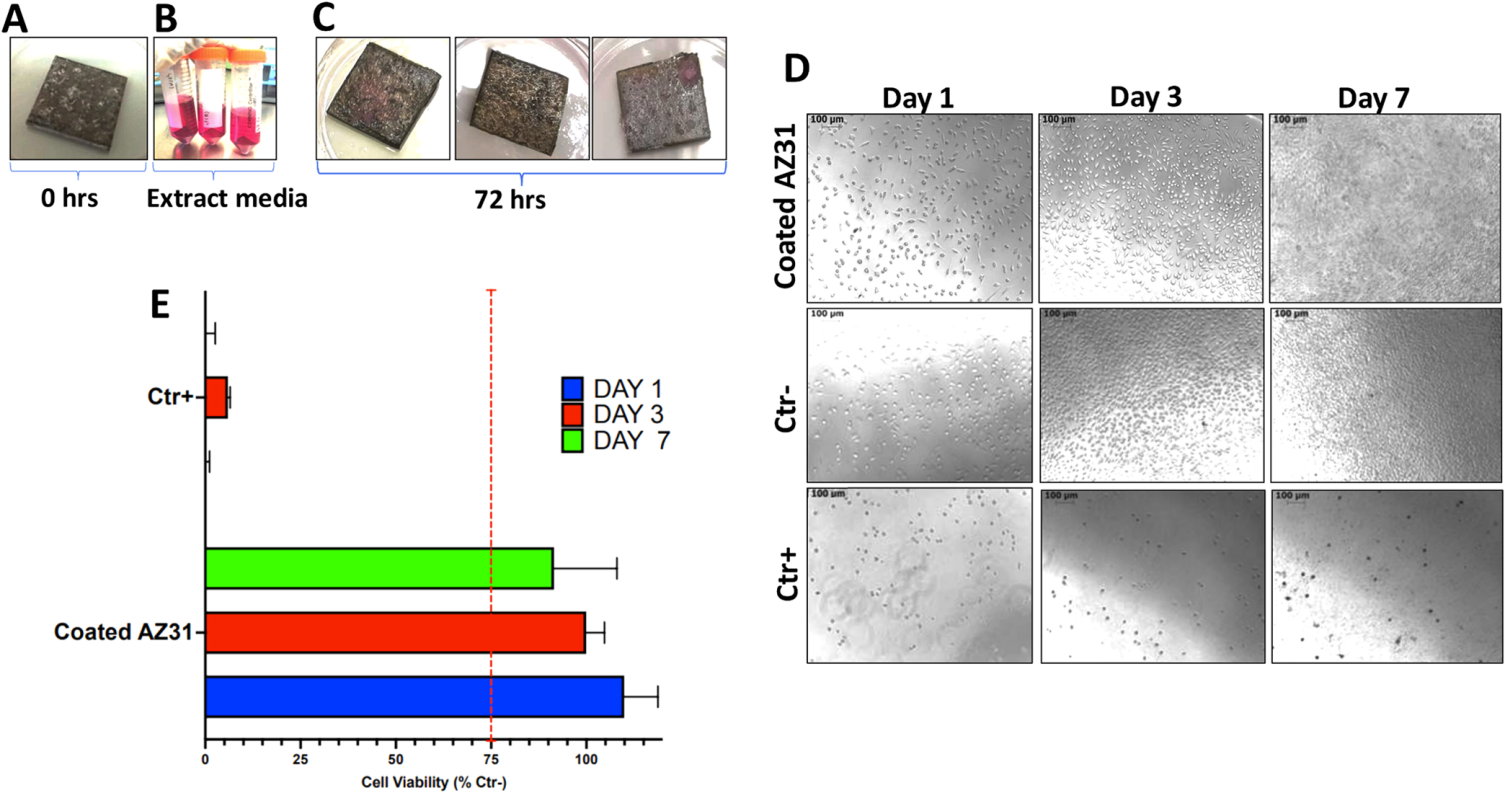
In vitro cytotoxicity test results for room-temperature-coated AZ31 samples obtained from Resazurin assay using L929 cells. (A) Coated AZ31 sample on *t*_0_ (0 h, before immersion in the culture medium); (B) extracts obtained after 72 h of incubation; (C) coated AZ31 samples on *t*_1_ (72 h, after immersion in the culture medium). (D) Morphology of L929 cells exposed to extract media, observed under a light microscope on days 1, 3, and 7. (E) Evaluation of L929 cell viability grown in extract media for 1, 3, and 7 days using the Resazurin assay. Ctr+: 10% DMSO solution.

The viability of L929 cells cultured in extract media was assessed after 1, 3, and 7 days. Figure 9D presents the microscopic evaluation of the cell morphology. At each assessed time point, treated cells exhibited morphology comparable to the negative control, suggesting a similar growth pattern between treated and untreated cells. Cell viability after 1, 3, and 7 days, as observed microscopically, was further confirmed by the PrestoBlue assay. Figure 9E illustrates that the cell viability (% relative to control) of L929 cells cultured in extracts from coated Mg AZ31 shows values comparable to the negative control. Specifically, cell viability was maintained at 109.95 ± 8.80% on day 1, 99.95 ± 4.79% on day 3, and 91.52 ± 16.49% on day 7. Accordingly, the coated AZ31 devices are considered cytocompatible in accordance with ISO standards, given that their cell viability exceeds the safety threshold of 75%. These findings underscore the high level of biocompatibility of the coated AZ31 devices.

## 4 Conclusions

Orthophosphoric acid (OPA) was used as the phosphate source in the cementitious aqueous suspensions that functioned as phosphatizing coatings on magnesium alloys after a facile dip-coating application. Extreme reactivity of both OPA and Mg in salt solutions was controlled by HEC, MgCl_2_, and Mg ion addition into the acidic solution. Two wt % HEC effectively slowed down the mass transport between the surface and the solution, while Mg saturation minimized the initial driving force for alloy degradation. Systematic study of various additives revealed that chloride salts also helped passivation, while nitrate salts accelerated the degradation of the alloy in 3.5% NaCl solution. The multicomponent suspension of 1.5 M OPA, 2 wt % HEC, 3 wt % MgCl_2_, and Mg-saturated water applied and dried on AZ31 plates at two temperatures was demonstrated to induce a favorable chemistry in the saline immersion medium for the evolution of the alloy surface to a passive composition within the 21-day immersion period. Cross-linking of HEC at 55 °C had an accelerating effect on the phase evolution, which reduced alloy degradation as a result. Phase analysis in conjunction with morphological analysis revealed that the coating initially converted the surface to newberyite. The phosphatizing hydrogel coating dissolved into the immersion medium within the first day and started a series of phase transformations with the chlorine ions infiltrating toward the alloy surface. The attack of chlorine on newberyite crystals was clearly observed, as they gradually converted to Mg oxychloride needles in a peeling fashion. 5–1–8 and 3–1–8 crystals formed both epitaxially and homogeneously on the surface within the first day. Then, they were replaced by a sudden trimagnesium phosphate formation after the third day. Hydrated trimagnesium phosphate phases cattiite and bobierrite were metastable between the third and 21st days of immersion as they were gradually replaced by the stable Mg hydroxide phase, brucite. Time-dependent evolution of the coated surfaces was correlated with the pH of the immersion medium, such that the transition from acidic to alkaline conditions facilitated their passivation. Such cementitious coatings and the evolving biomimetic, potentially bioactive surfaces they provide are promising alternatives to inert ceramic coatings on magnesium implants due to their ability to self-passivate in a physiological medium and their capacity for sustained release of biochemicals.

## Supplementary Material



## Notes

Detailed morphological and composition data including scanning electron microscope and optical microscope images of the coated specimen surfaces, elemental analysis of the surfaces by EDX, and quantitative XRD analysis of the surfaces; raw electrochemical and direct corrosion test data for various samples (PDF)
