# Supplementary material for: Design and Characterization of Phosphatizing Coatings for Magnesium Implants
Source: ACS Biomater Sci Eng. 2026 Feb 13;12(3):1492–507. doi: 10.1021/acsbiomaterials.5c01846 (PMC12976993; doi:10.1021/acsbiomaterials.5c01846)
Supplement: Supplementary file 1 [file ab5c01846_si_001.pdf]

Supplementary information to:

Design and characterization of phosphatizing coatings for magnesium implants

Erdem Şahin*[a], Francesco Paduano*[b], Marco Tatullo[c], Roberta Ruggiero[b], Elisabetta Aiello[b], Rosa Maria Marano[b], Meltem Alp[a], Ahmed Şeref[a]

[a]*Department of Metallurgical and Materials Engineering, Muğla Sıtkı Koçman University, 48000 Muğla, Türkiye*

[b]*Stem Cells and Medical Genetics Units, Tecnologica Research Institute and Marrelli Health, 88900 Crotone, Italy*

[c]*Department of Translational Biomedicine and Neuroscience, School of Medicine, University of Bari "Aldo Moro", 70124 Bari, Italy*

*** Corresponding authors:**

Erdem Şahin, Department of Metallurgical and Materials Engineering, Muğla Sıtkı Koçman University, 48000 Muğla, Türkiye.

Tel: +90 0252 211 5584

E mail: erdemsahin@mu.edu.tr

Stem Cells and Medical Genetics Units, Tecnologica Research Institute and Marrelli Health, 88900 Crotone, Italy

E mail: francesco.paduano@tecnologicasrl.com

Contents

EDX analysis of AZ31 plate immersed in 1.5M OPA solution for 21 days (Table S1);

Macroscopic views of the immersion solution containing OPA and HEC (Figure S1);

EDX analysis of the AZ31 plate kept for 21 days in 1.5M OPA solution containing 2wt% HEC (Table S2);

EDX analysis of the AZ31 plate kept for 21 days in 1.5M OPA solution containing 3wt% NaCl (Table S3);

SEM images of the AZ31 plate kept for 21 days in 1.5M OPA solution containing 3wt% NaCl (Figure S2);

SEM images of the AZ31 plate kept for 21 days in 1.5M OPA solution containing 3wt% $MgCl_2$ (Figure S3);

EDX analysis of the AZ31 plate kept for 21 days in 1.5M OPA solution containing 3wt% $MgCl_2$ (Table S4);

EDX analysis of the AZ31 plate kept for 21 days in 1.5M OPA solution containing 3wt% Mg nitrate (Table S5);

SEM images of the AZ31 plate kept for 21 days in 1.5M OPA solution containing 3wt% $Mg(NO_3)_2$ (Figure S4);

SEM images of the AZ31 plate kept for 21 days in 1.5M OPA solution containing 3wt% Ca nitrate (Figure S5);

SEM images of AZ31 plate immersed in 1.5M OPA solution with 3% Ca nitrate (Table S6);

EDX analysis of AZ31 plate immersed in 1.5M OPA and 0.1M trisodium citrate solution (Table S7);

SEM images of AZ31 sample immersed in 1.5M OPA and 0.1M trisodium citrate solution for 21 days (Figure S6);

SEM images of the AZ31 plate kept for 21 days in 1.5M OPA solution saturated with Mg (Figure S7);

EDX analysis of AZ31 plate immersed in 1.5M OPA saturated with Mg (Table S8);

Phase composition deposited on the surface of AZ31 plate kept for 21 days in 1.5M OPA solution containing 2wt% HEC saturated with Mg (Table S9);

SEM images of the AZ31 plate kept for 21 days in 1.5M OPA, 3wt% NaCl solution saturated with Mg (Figure S8);

EDX analysis of AZ31 plate kept for 21 days in 1.5M OPA solution containing 3wt% NaCl saturated with Mg (Table S10);

SEM images of the AZ31 plate kept for 21 days in 1.5M OPA, 3wt% $MgCl_2$ solution saturated with Mg (Figure S9);

EDX analysis of AZ31 plate kept for 21 days in 1.5M OPA solution containing 3wt% $MgCl_2$ saturated with Mg (Table S11); SEM images of the AZ31 plate kept for 21 days in 1.5M OPA, 3wt% Mg nitrate solution saturated with Mg (Figure S10);

EDX analysis of AZ31 plate kept for 21 days in 1.5M OPA solution containing 3wt% Mg nitrate saturated with Mg (Table S12);

SEM images of the AZ31 plate kept for 21 days in 1.5M OPA, 3wt% Ca nitrate solution saturated with Mg (Figure S11);

EDX analysis of AZ31 plate kept for 21 days in 1.5M OPA solution containing 3wt% Ca nitrate saturated with Mg (Table S13); Gravimetric analysis of AZ31 plates immersed in three component solutions for 21 days (Figure S12);

Electrochemical test results of AZ31 plates immersed in three component solutions for 21 days (Table S14);

The phase structure accumulated on the surface of the AZ31 plate kept in a solution containing OPA, $MgCl_2$, and HEC for 21 days (Figure S13);

EDX analysis of the AZ31 plate kept in the solution containing OPA, $MgCl_2$ and HEC for 21 days (Table S15);

SEM images of the AZ31 plate kept in a solution containing OPA, Citrate, and $MgCl_2$ for 21 days (Figure S14);
EDX analysis of the AZ31 plate kept in the solution containing OPA, Citrate and $MgCl_2$ for 21 days (Table S16);
SEM images of the AZ31 plate kept in a solution containing OPA, $MgNO_3$, and Citrate for 21 days (Figure S15);
EDX analysis of the AZ31 plate kept in the solution containing OPA, $MgNO_3$, and Citrate for 21 days (Table S17);
SEM images of the AZ31 plate kept in a solution containing OPA, Citrate, and HEC for 21 days (Figure S16);
EDX analysis of the AZ31 plate kept in the solution containing OPA, Citrate and HEC for 21 days (Table S18);
Coating thickness of RTC and HTC samples measured under 40X magnification (Figures S17-S18);
SEM images of RTC samples upon immersion in 3.5% NaCl solution at various immersion periods (Figure S19);
Variations in the anion/cation ratio of RTC samples upon immersion in 3.5% NaCl solution (Figures S20-S21);
Open circuit potentials of RTC and HTC samples immersed in 3.5% NaCl solution for various periods (Figures S22, S24);
Potential scan curves of RTC and RTC samples immersed in 3.5% NaCl solution for various periods (Figures S23, S25);
SEM images of HTC and RTC samples that were not immersed in 3.5% NaCl solution prior to the corrosion test (Figures S26-S27);
SEM images of HTC samples after 1 day immersion in 3.5% NaCl solution prior to the corrosion test (Figure S28);
SEM images of HTC samples after 3 day immersion in 3.5% NaCl solution prior to the corrosion test (Figure S29);
SEM images of RTC and HTC samples after 7 day immersion in 3.5% NaCl solution prior to the corrosion test (Figures S30-S31);
SEM images of HTC samples after 14 day immersion in 3.5% NaCl solution prior to the corrosion test (Figure S32);
SEM images of HTC samples after 21 day immersion in 3.5% NaCl solution prior to the corrosion test Figures S33-S34);
SEM images of RTC samples after 21 day immersion in 3.5% NaCl solution prior to the corrosion test (Figure S35) (PDF)

Table S1. EDX analysis of AZ31 plate immersed in 1.5M OPA solution for 21 days.

Surface morphology	EDX spectroscopy (weight and atomic ratio)
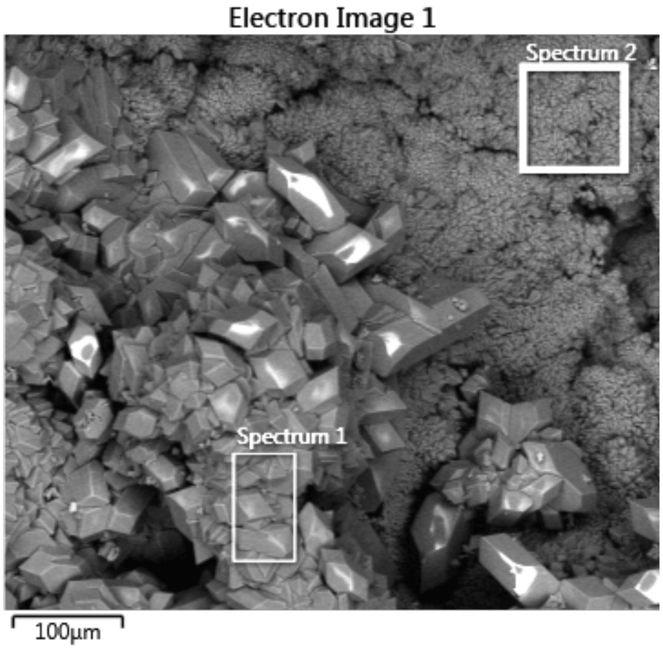	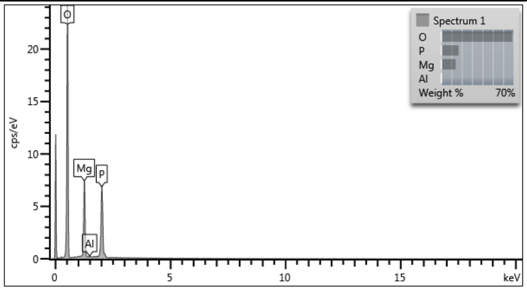 Mg/P/O=10.5/10/79
	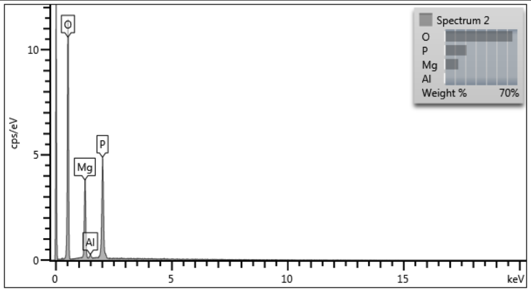 Mg/P/O=10/13/76.5

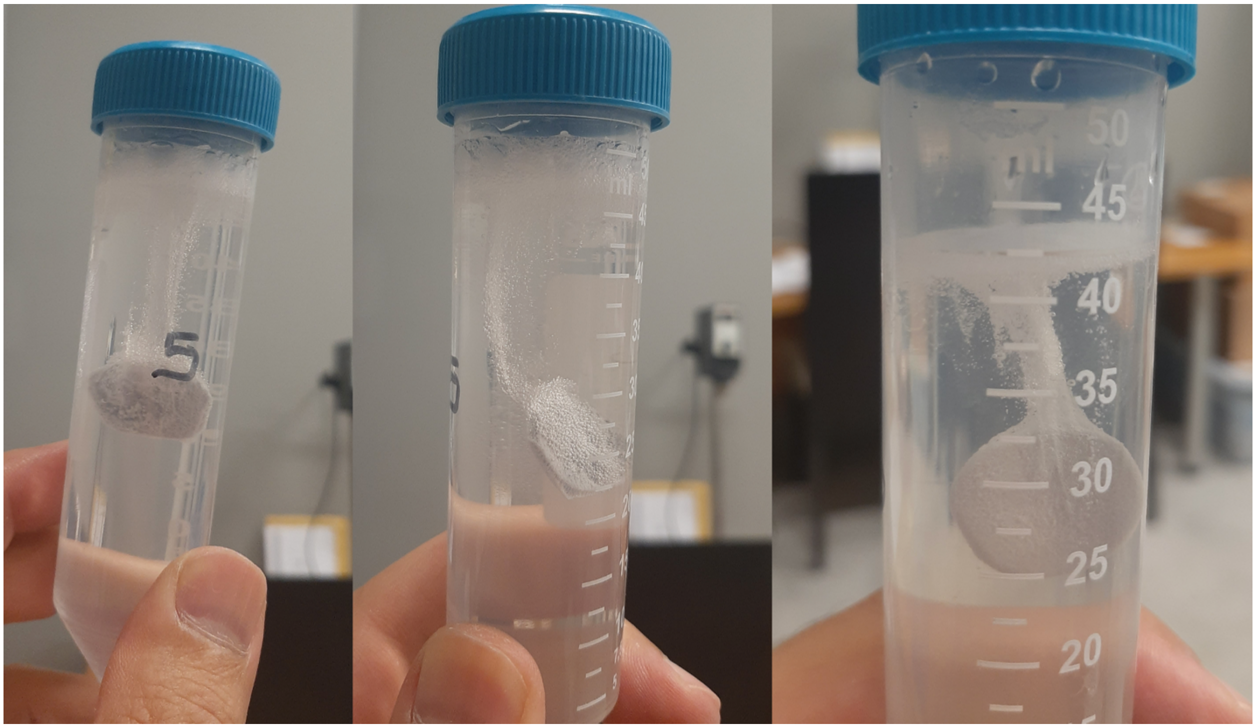

Figure S1. Macroscopic views of the immersion solution containing orthophosphoric acid and hydroxyethyl cellulose.

Table S2. Elemental composition deposited on the surface of the AZ31 plate kept for 21 days in 1.5M OPA solution containing 2wt% HEC.

Surface morphology	EDX spectroscopy (weight and atomic ratio)
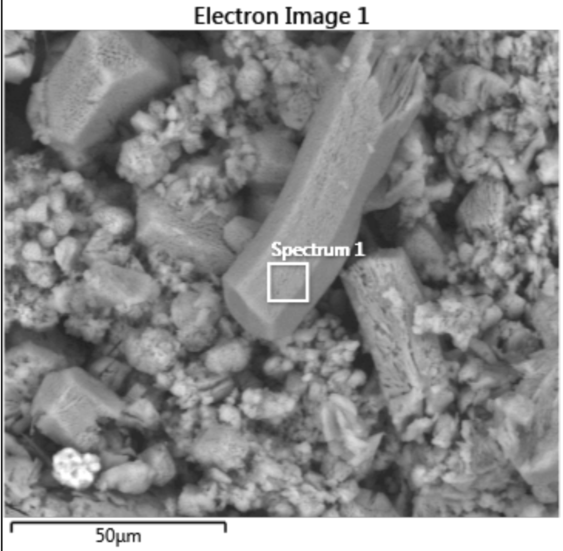	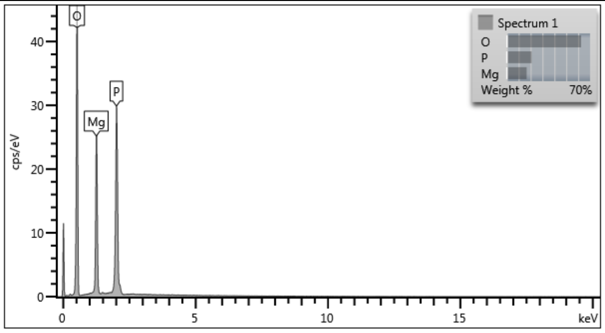<br>Mg/P/O=13/13/74

Table S3. Elemental composition deposited on the surface of the AZ31 plate kept for 21 days in 1.5M OPA solution containing 3wt% NaCl.

Surface morphology	EDX spectroscopy (weight and atomic ratio)
	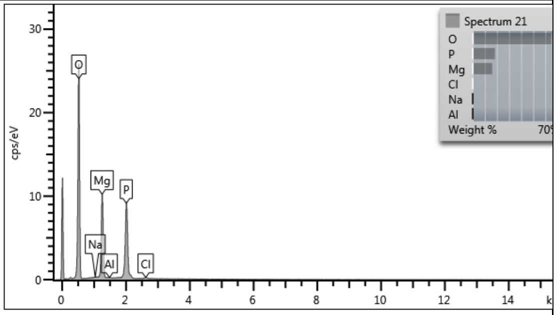<br>Mg/P/O= 12/11/76
	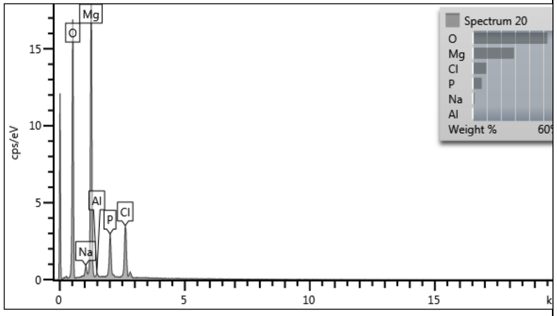<br>Mg/P/O/Cl= 24/4/65/5

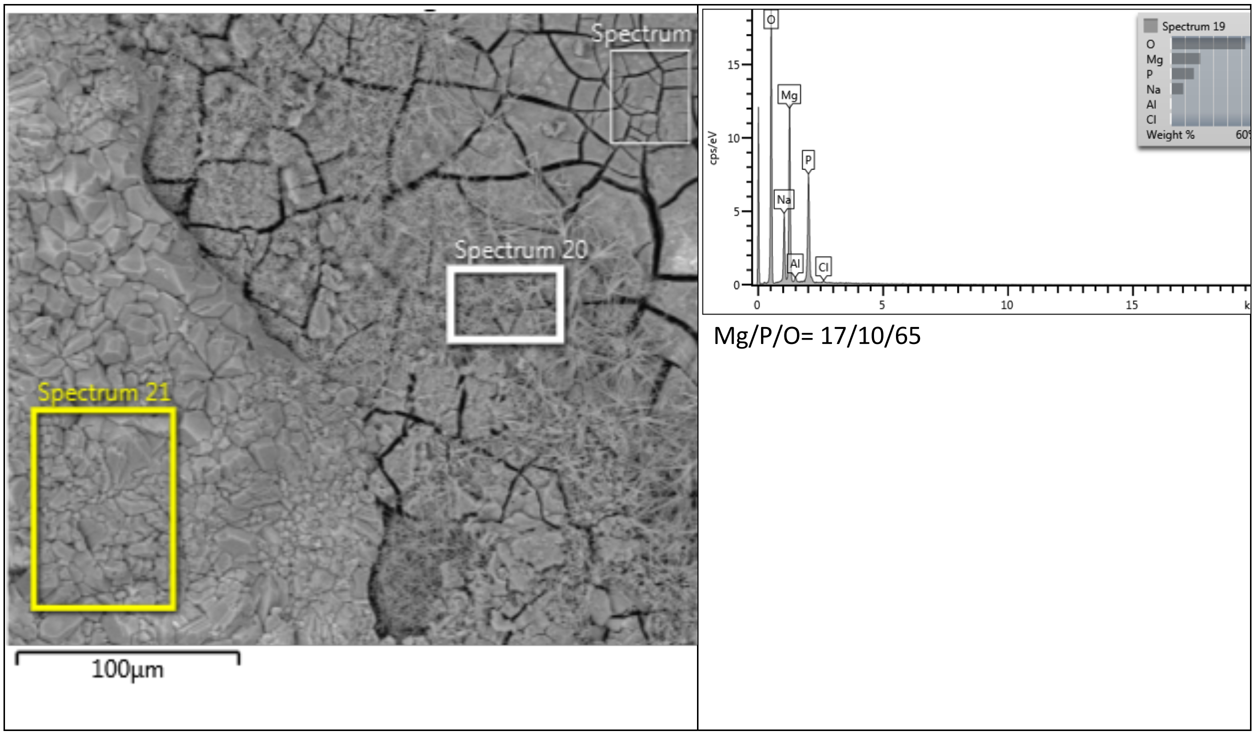

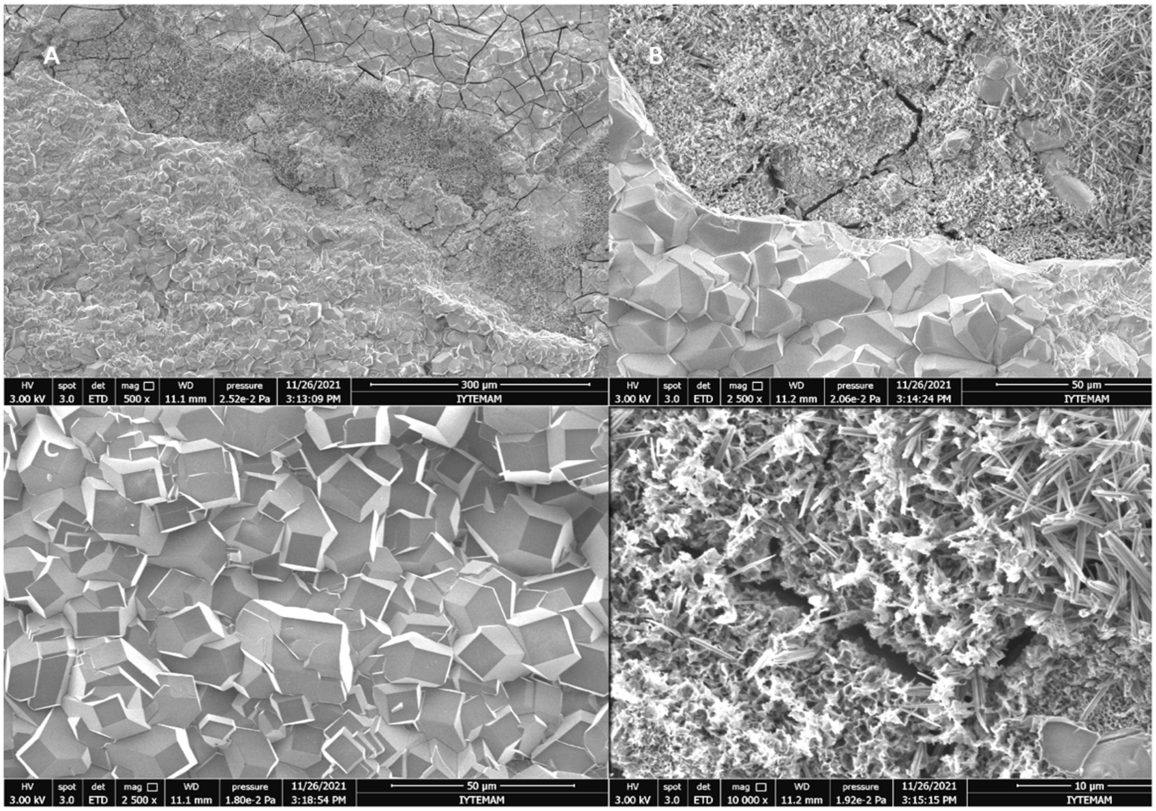

Figure S2. Surface morphology of the AZ31 plate kept for 21 days in 1.5M OPA solution containing 3wt% NaCl: (A) 500X, (B) 2500X, (C) 2500X, (D) 10000X.

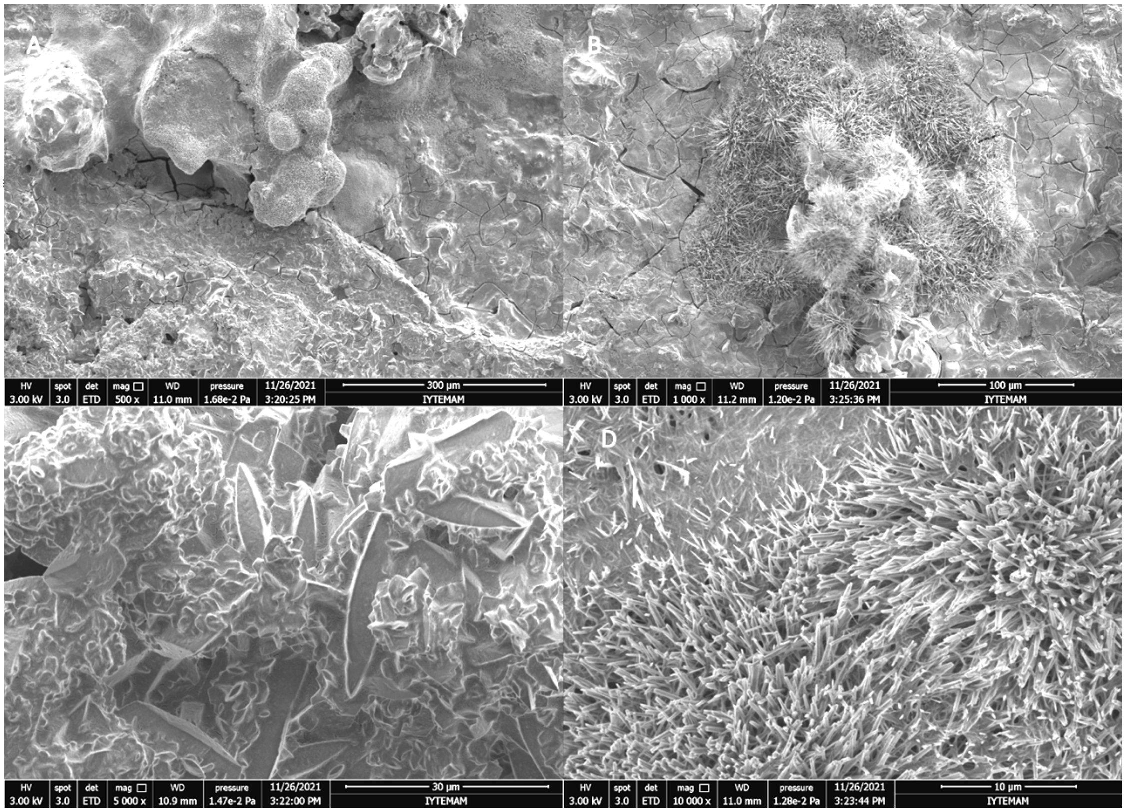

Figure S3. Surface morphology of the AZ31 plate kept for 21 days in 1.5M OPA solution containing 3wt% $MgCl_2$: (A) 500X, (B) 1000X, (C) 5000X, (D) 10000X.

Table S4. Elemental composition deposited on the surface of the AZ31 plate kept for 21 days in 1.5M OPA solution containing 3wt% $MgCl_2$.

Surface morphology	EDX spectroscopy (weight and atomic ratio)
	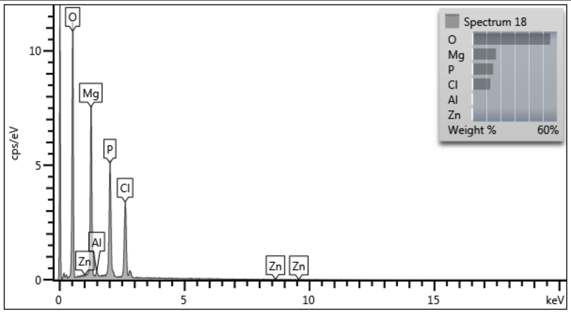 Mg/P/O/Cl= 14/10/69/7

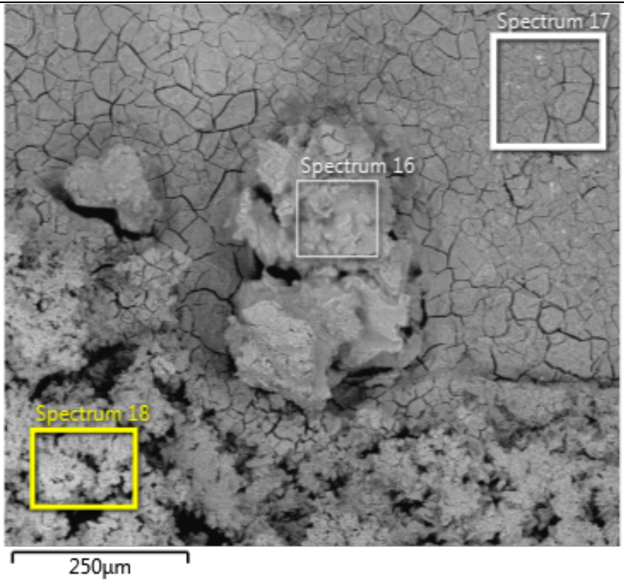	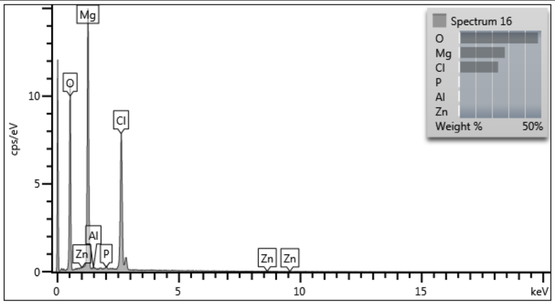<br>Mg/P/O/Cl= 24/0/62/14
	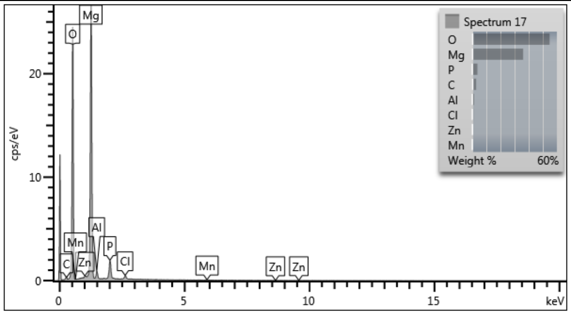<br>Mg/P/O= 28/2/64

Table S5. Elemental composition deposited on the surface of the AZ31 plate kept for 21 days in 1.5M OPA solution containing 3wt% Mg nitrate.

Surface morphology	EDX spectroscopy (weight and atomic ratio)
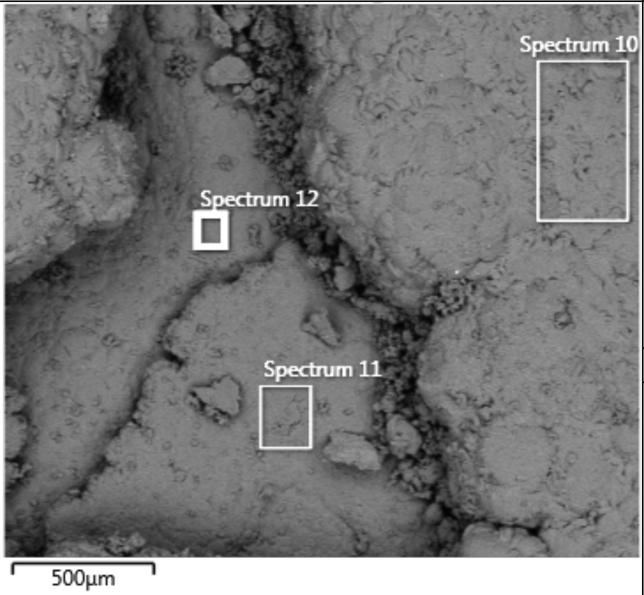	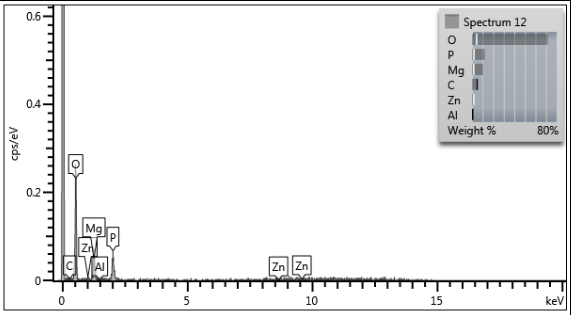<br>Mg/P/O= 8/7/79
	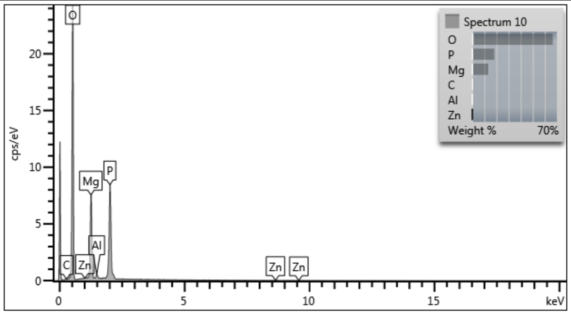<br>Mg/P/O= 10/11/77

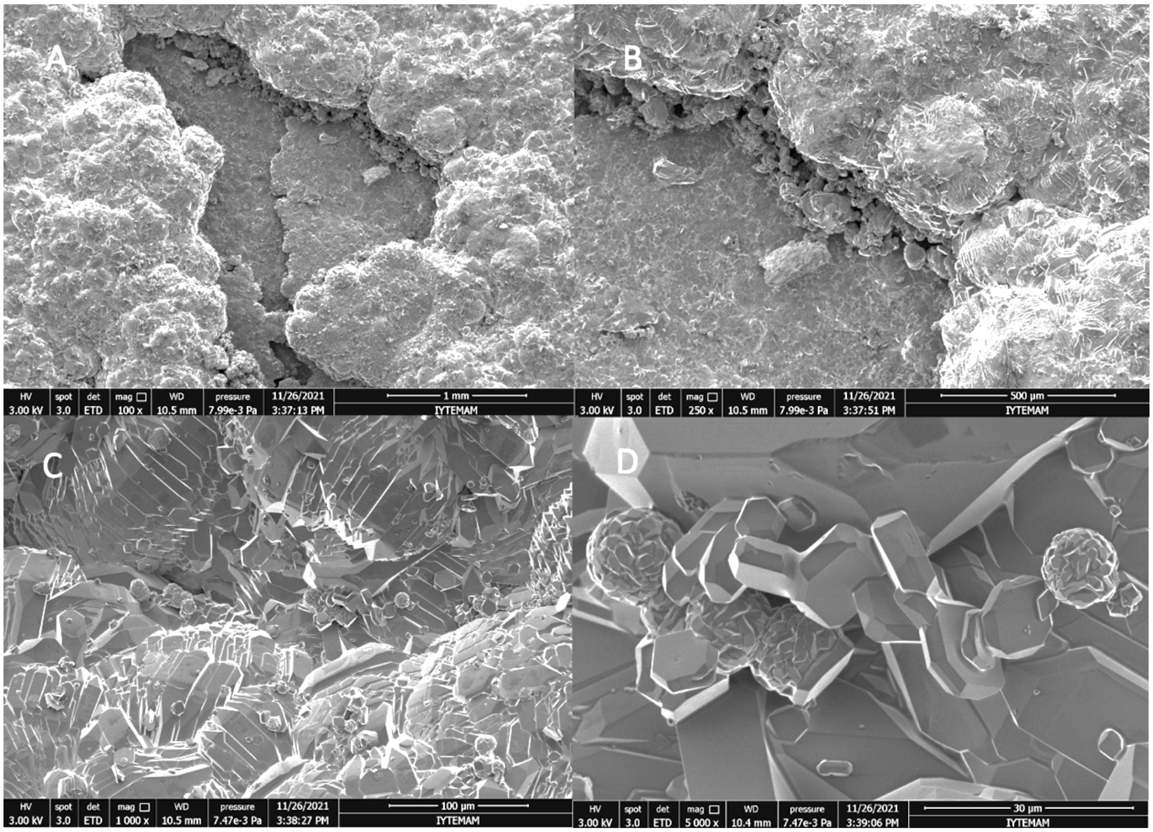

Figure S4. Surface morphology of the AZ31 plate kept for 21 days in 1.5M OPA solution containing 3wt% $Mg(NO_3)_2$: (A) 100X, (B) 250X, (C) 1000X, (D) 5000X.

Table S6. Surface analysis of AZ31 plate immersed in 1.5M OPA solution with 3% Ca nitrate.

Surface morphology	EDX spectroscopy (weight and atomic ratio)
	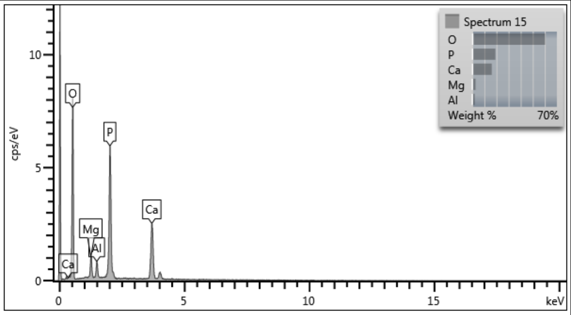 Mg/P/O/Ca/Al= 2/12/76/8/1

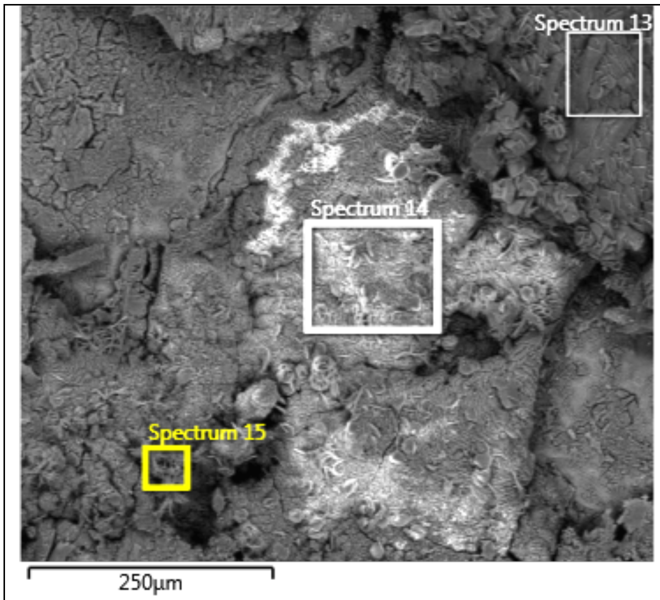

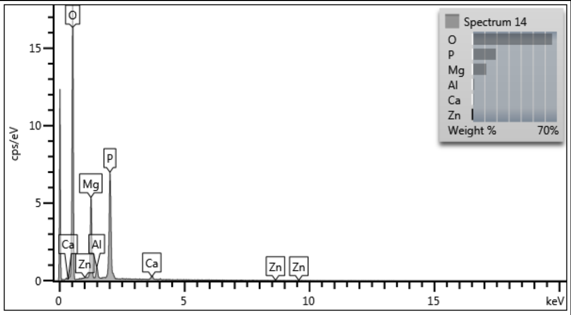

Mg/P/O/Al= 9/12/77/1

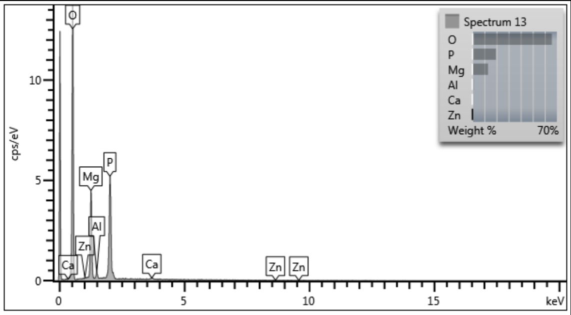

Mg/P/O/Al= 10/12/77/1

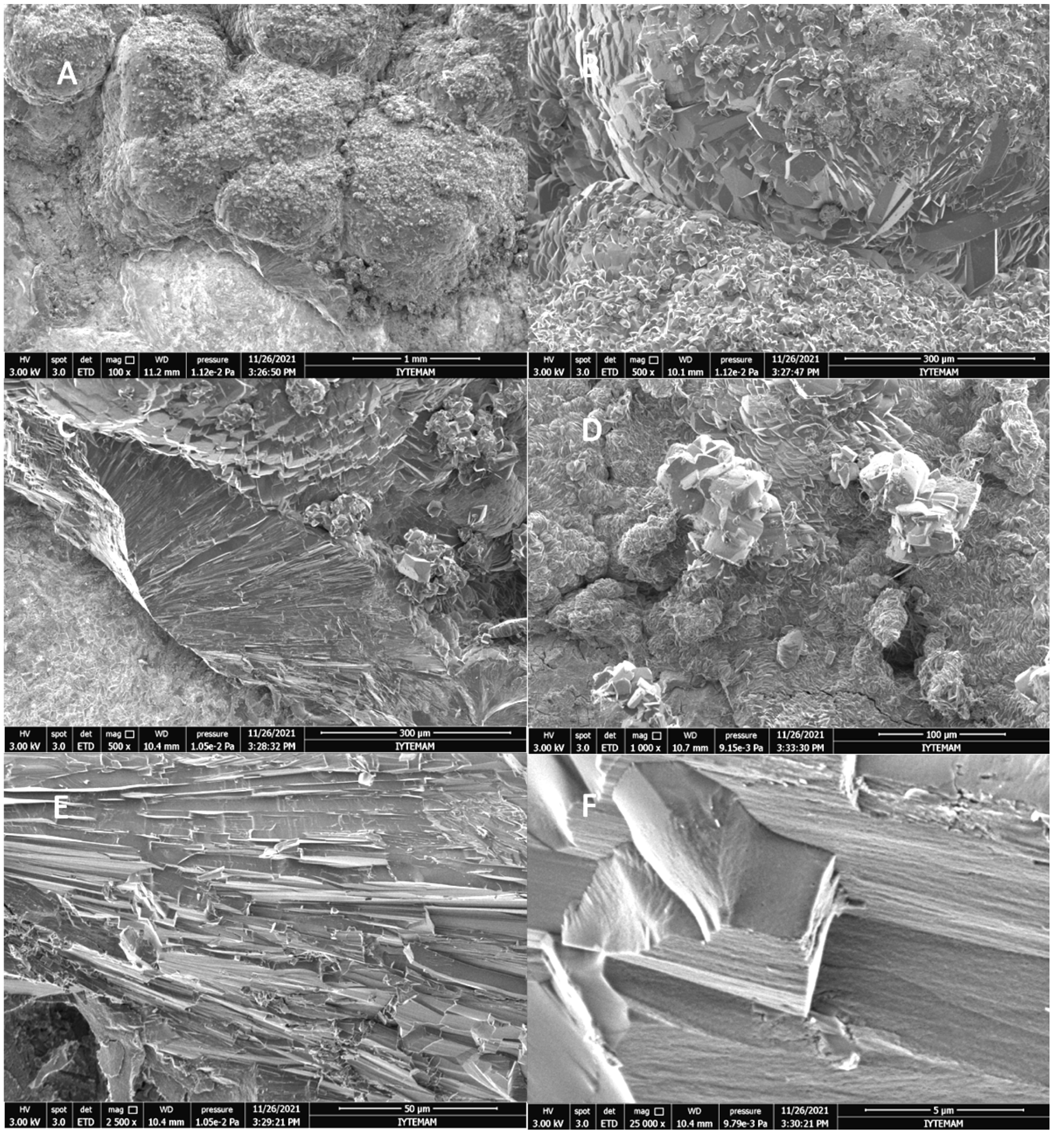

Figure S5. Surface morphology of AZ31 sample immersed in 1.5M OPA solution with 3% Ca nitrate for 21 days: (A) 100X, (B) 500X, (C) 500X, (D) 1000X, (E) 2500X, (F) 25000X.

Table S7. Surface analysis of AZ31 plate immersed in 1.5M OPA and 0.1M trisodium citrate solution.

Surface morphology	EDX spectroscopy (weight and atomic ratio)
	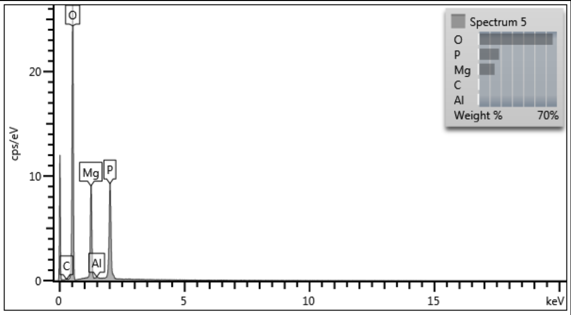 Mg/P/O= 11/11/77

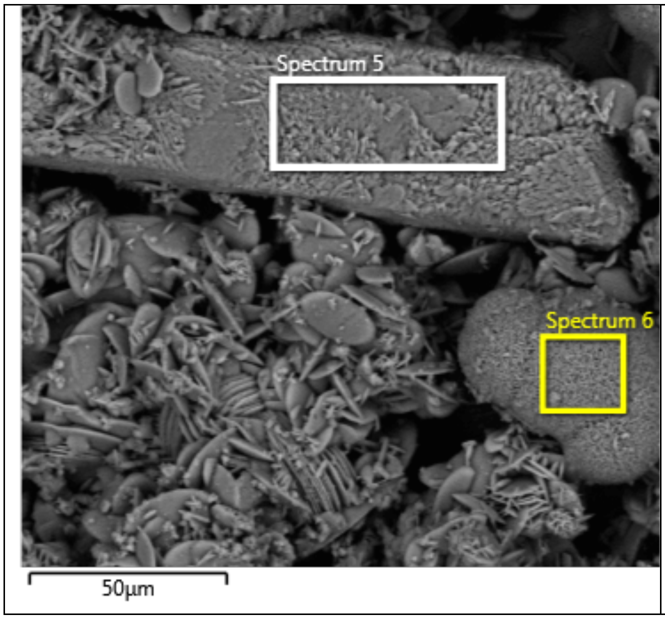

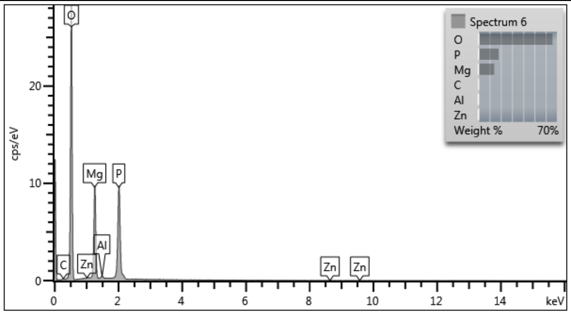

Mg/P/O= 11/11/76

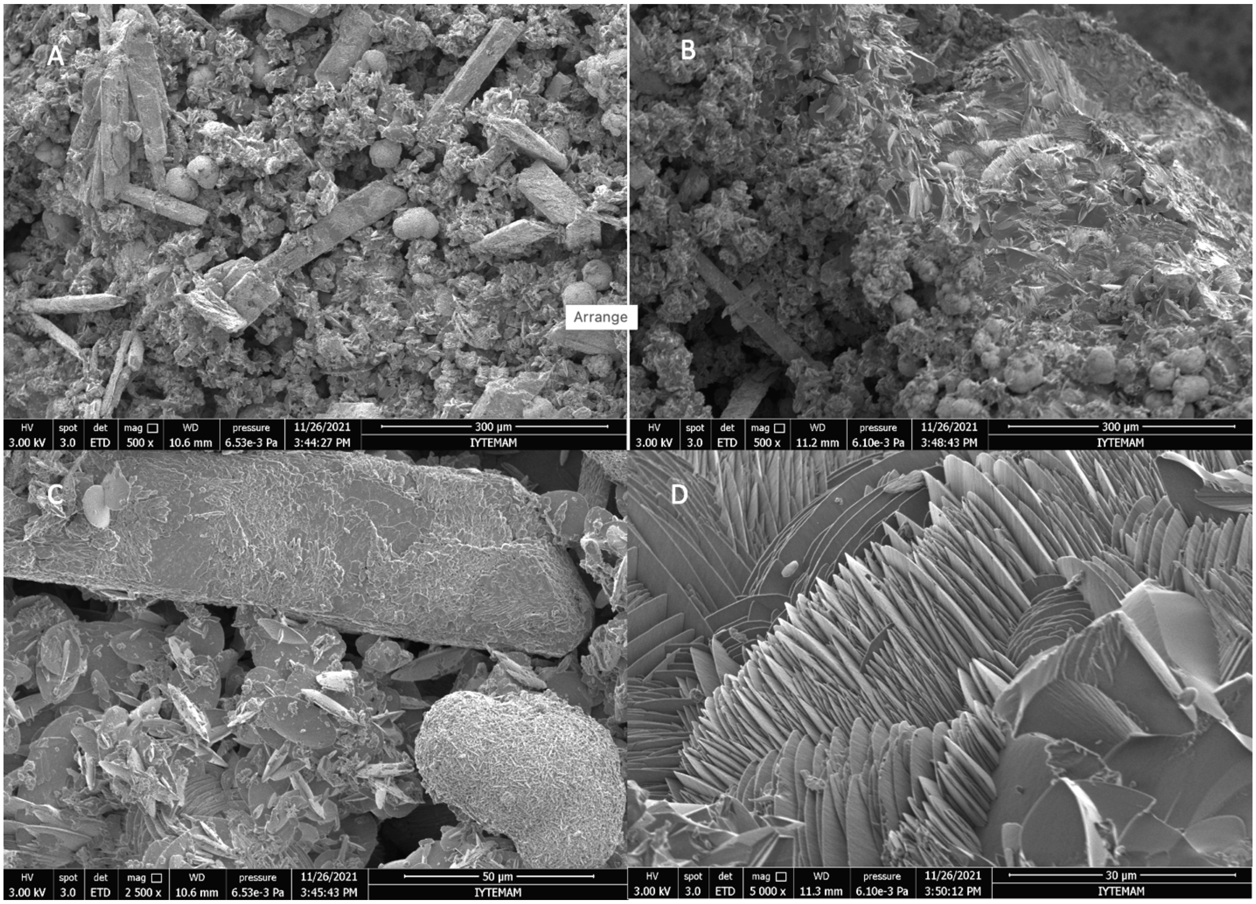

Figure S6. Surface morphology of AZ31 sample immersed in 1.5M OPA and 0.1M trisodium citrate solution for 21 days: (A) 500X, (B) 500X, (C) 2500X, (D) 5000X.

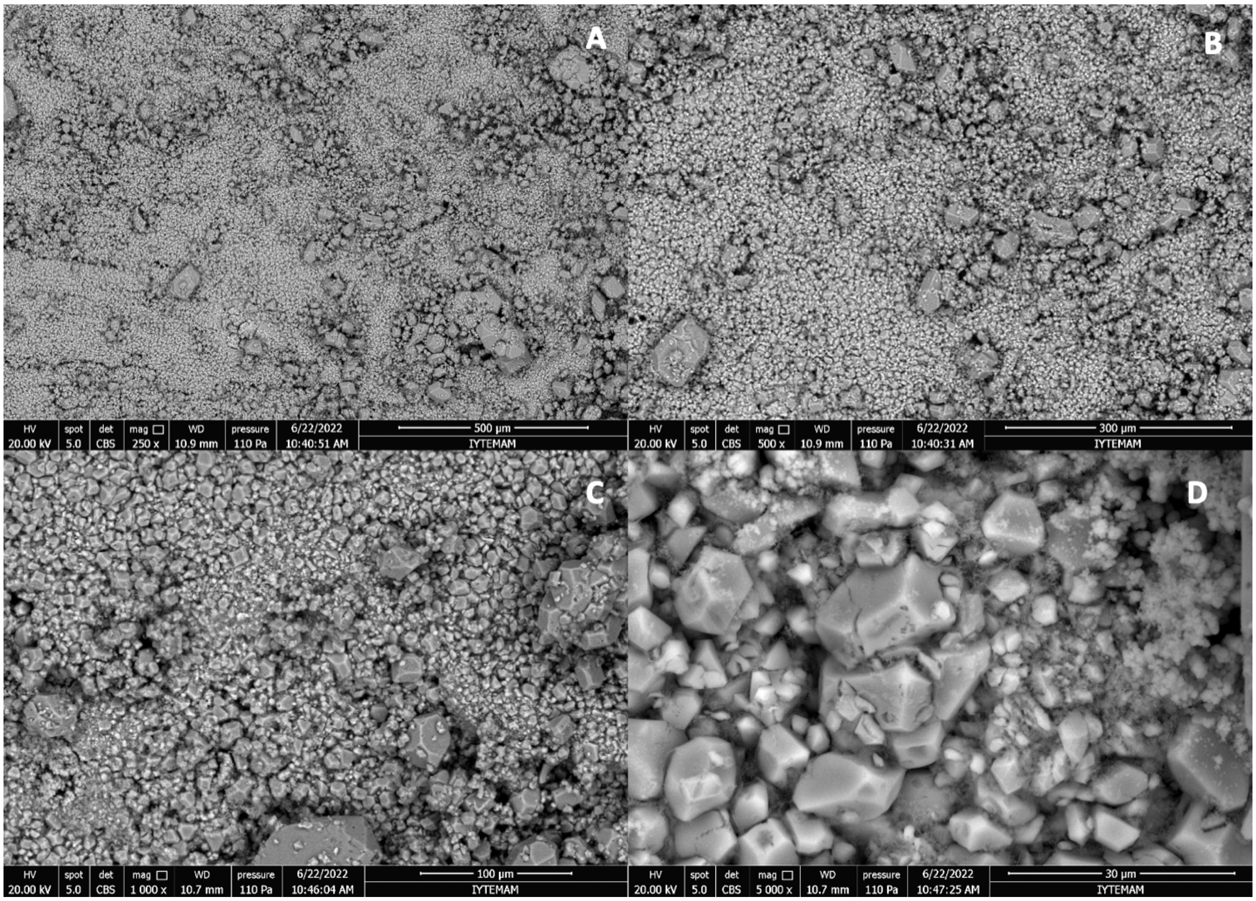

Figure S7. Surface morphology of the AZ31 plate kept for 21 days in 1.5M OPA solution saturated with Mg: A) 500X, B) 500X, C) 2000X, D) 5000X.

Table S8. Surface analysis of AZ31 plate immersed in 1.5M OPA saturated with Mg.

Surface morphology	EDX spectroscopy (weight and atomic ratio)
	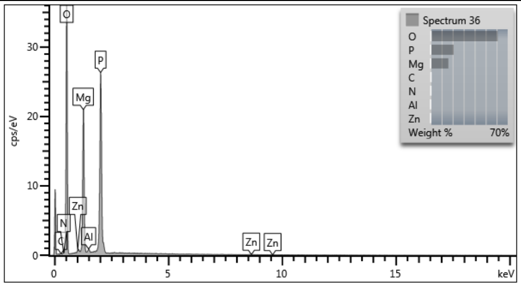 Mg/P/O=13/13/73

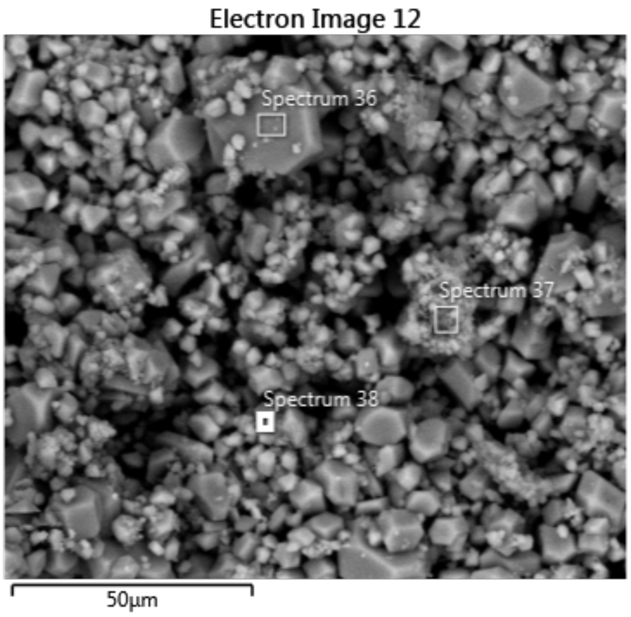	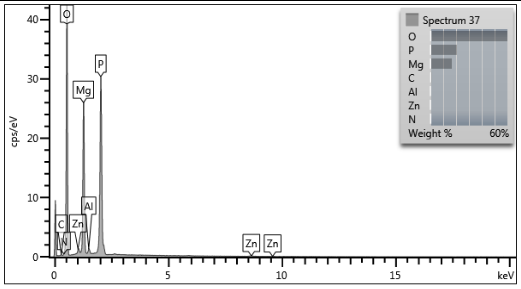<br>Mg/P/O=13/12.5/71
	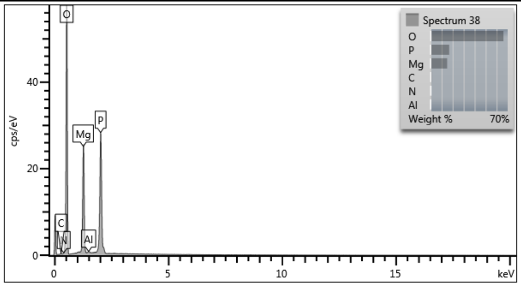<br>Mg/P/O=11.5/10/76

Table S9. Phase composition deposited on the surface of AZ31 plate kept for 21 days in 1.5M OPA solution containing 2wt% HEC saturated with Mg.

Surface morphology	EDX spectroscopy (weight and atomic ratio)
	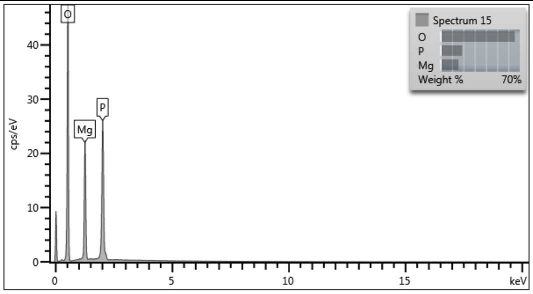<br>Mg/P/O=12/11.5/76.5

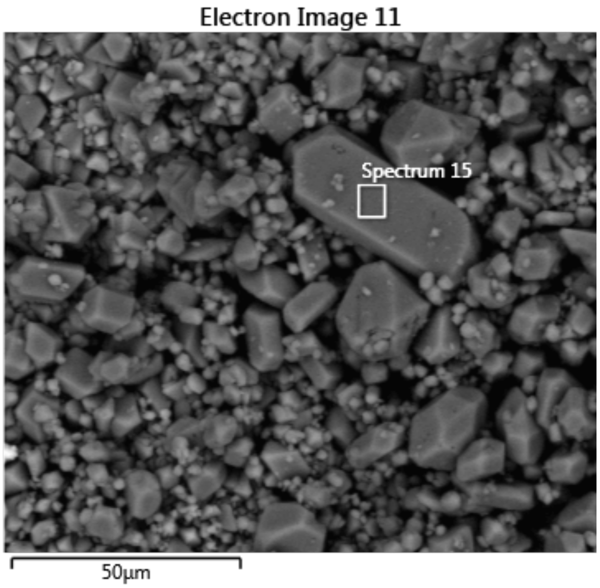

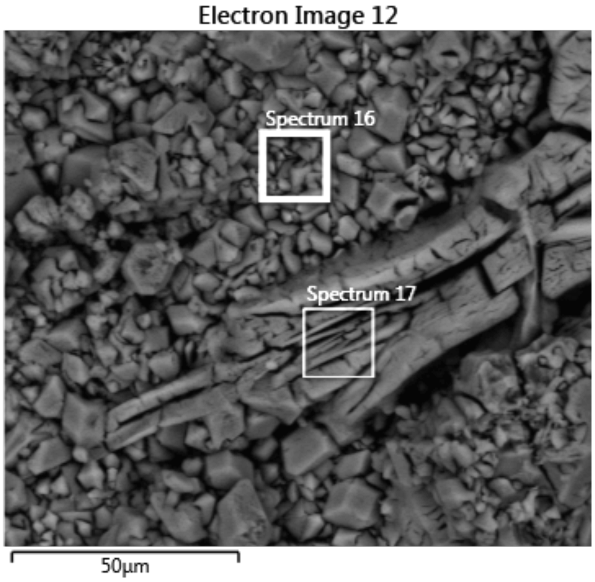

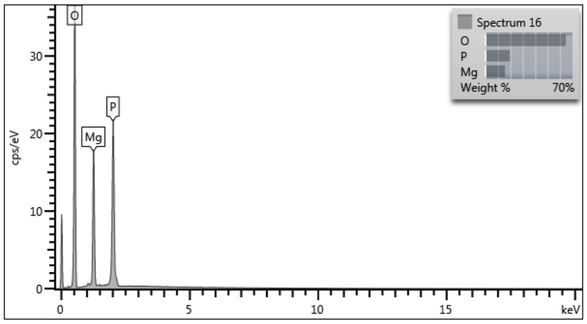

Mg/P/O=12/12/76

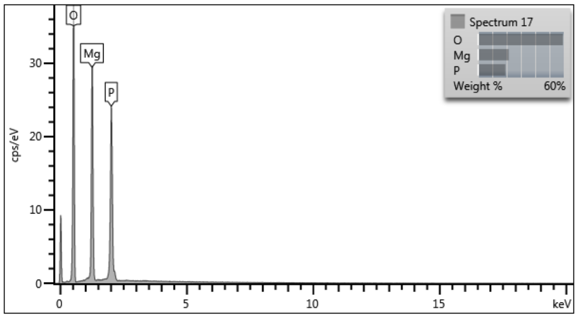

Mg/P/O=17/12/71

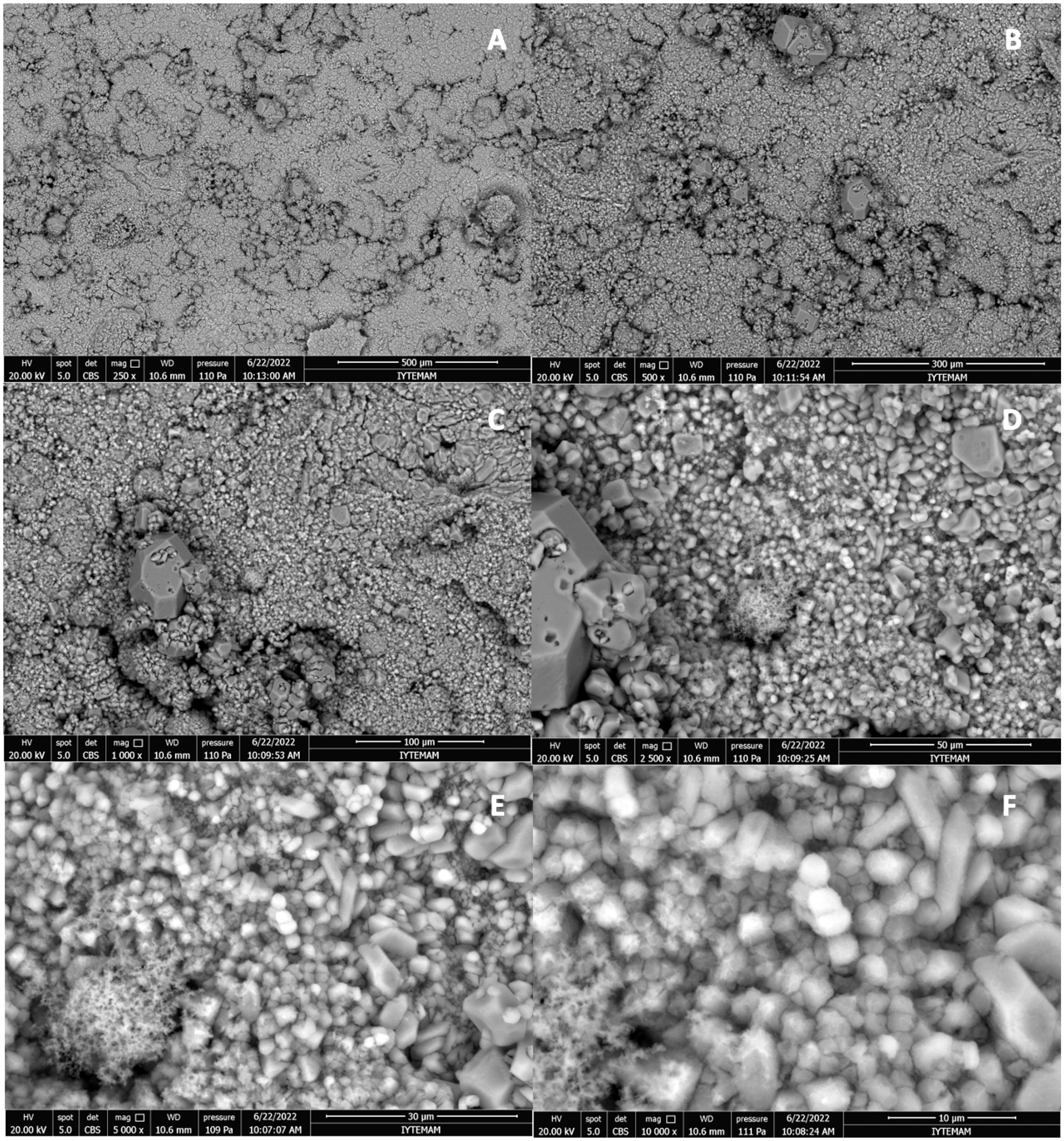

Figure S8. Surface morphology of the AZ31 plate kept for 21 days in 1.5M OPA, 3wt% NaCl solution saturated with Mg: A) 500X, B) 500X, C)2000X, D) 4000X, E) 6000X, F) 10000X.

Table S10. Phase composition deposited on the surface of AZ31 plate kept for 21 days in 1.5M OPA solution containing 3wt% NaCl saturated with Mg.

<table>
<tr><td>Surface morphology</td><td>EDX spectroscopy (weight and atomic ratio)</td></tr>
<tr><td rowspan="4">
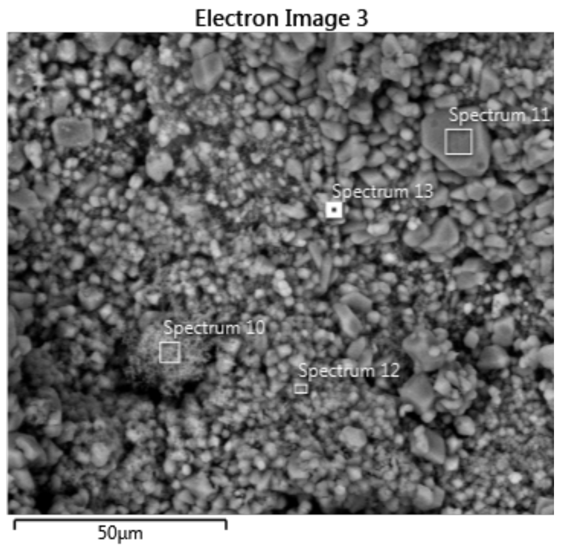

</td><td>
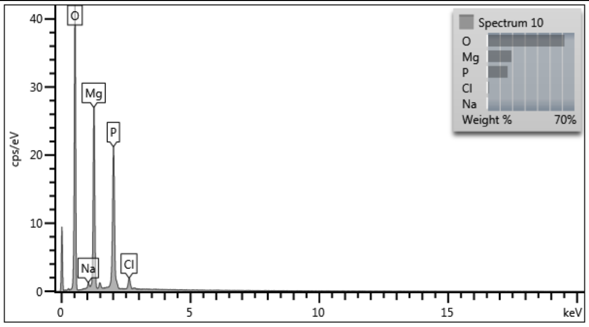

Mg/P/O=15/10/74 Cl=1
</td></tr>
<tr><td>
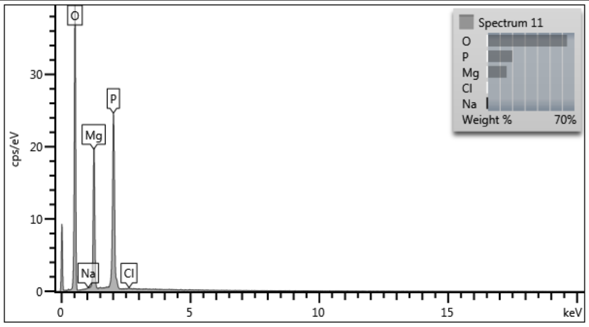

Mg/P/O=12/12.5/76
</td></tr>
<tr><td>
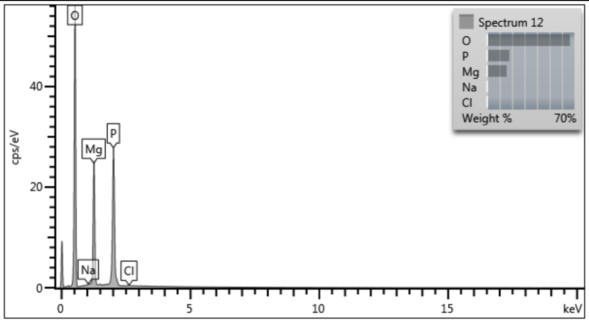

Mg/P/O=12/11/77
</td></tr>
<tr><td>
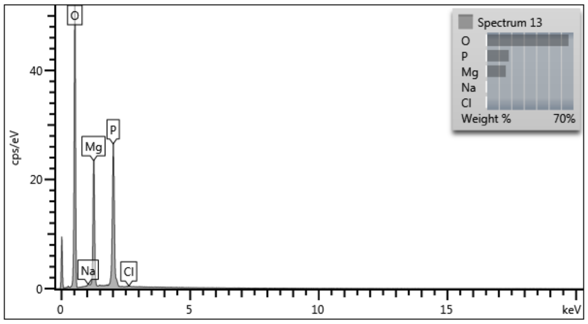

Mg/P/O=12/11/77
</td></tr>
</table>

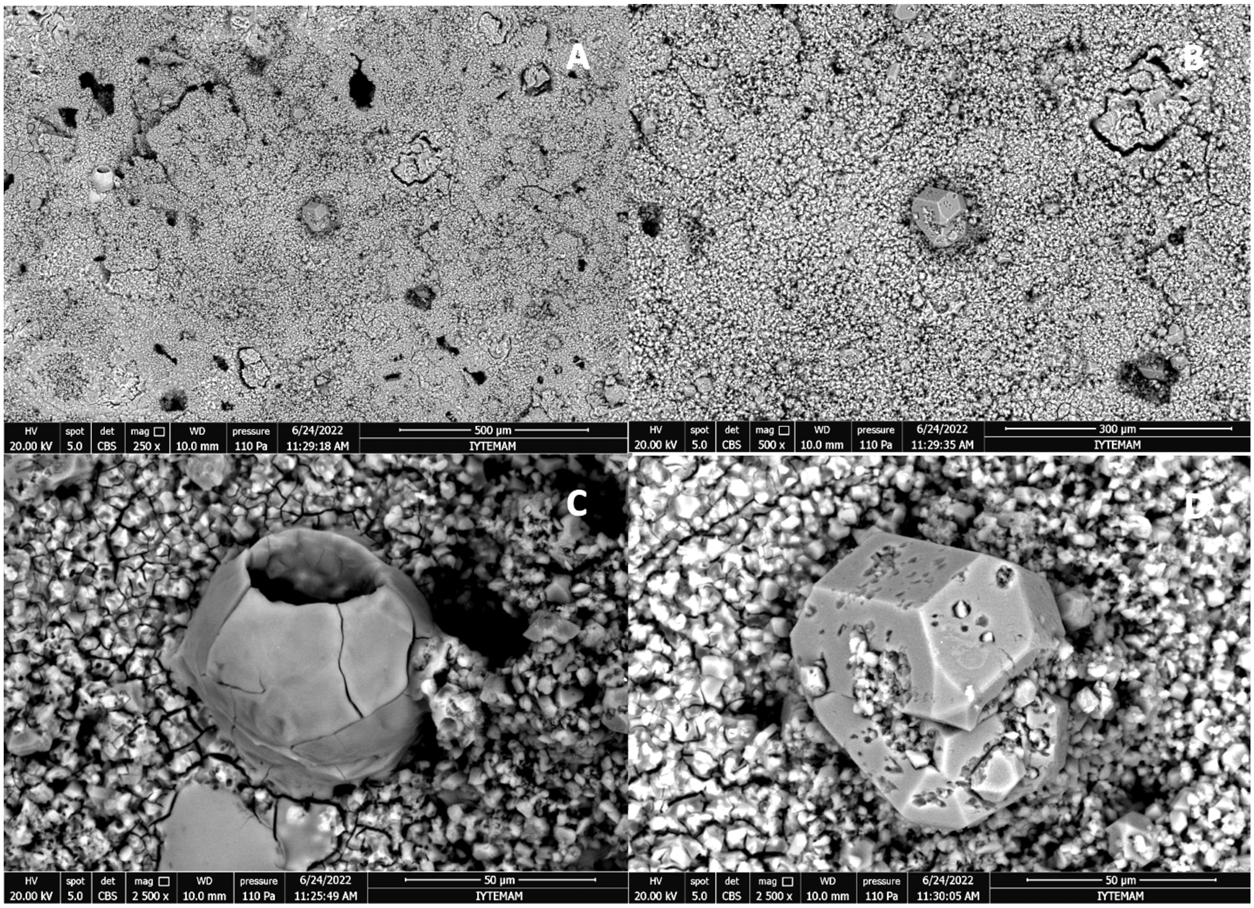

Figure S9. Surface morphology of the AZ31 plate kept for 21 days in 1.5M OPA, 3wt% $MgCl_2$ solution saturated with Mg: A) 500X, B) 500X, C)5000X, D) 4000X.

Table S11. Phase composition deposited on the surface of AZ31 plate kept for 21 days in 1.5M OPA solution containing 3wt% $MgCl_2$ saturated with Mg.

Surface morphology	EDX spectroscopy (weight and atomic ratio)
	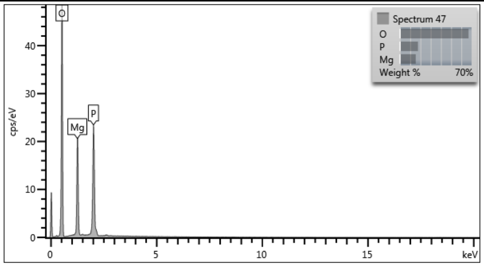 Mg/P/O=12/11/78
	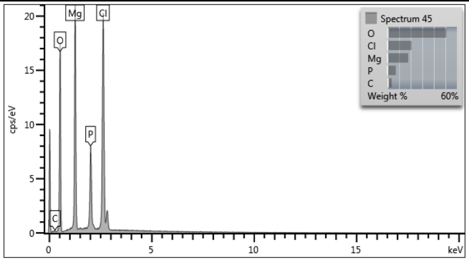 Mg/P/O=15/5/64 Cl=12

Electron Image 26

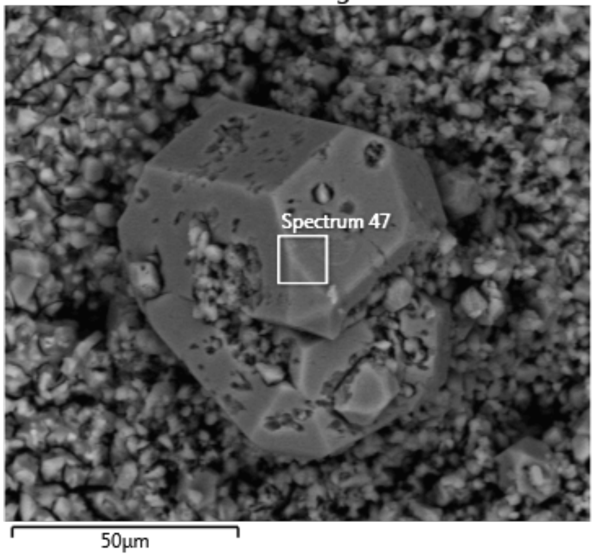

Electron Image 25

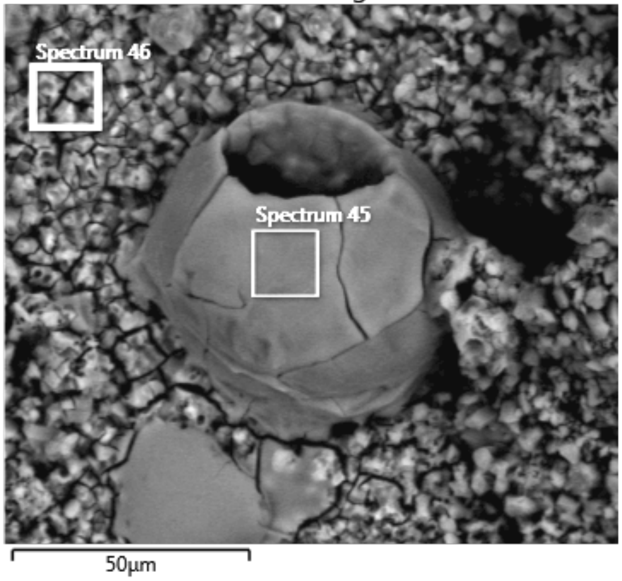

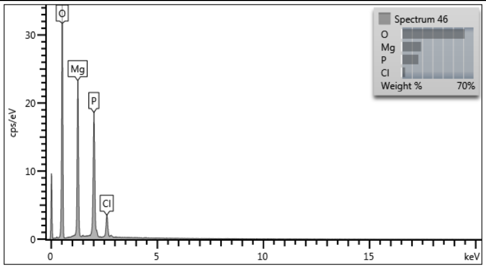

Mg/P/O=15/10/73 Cl=2

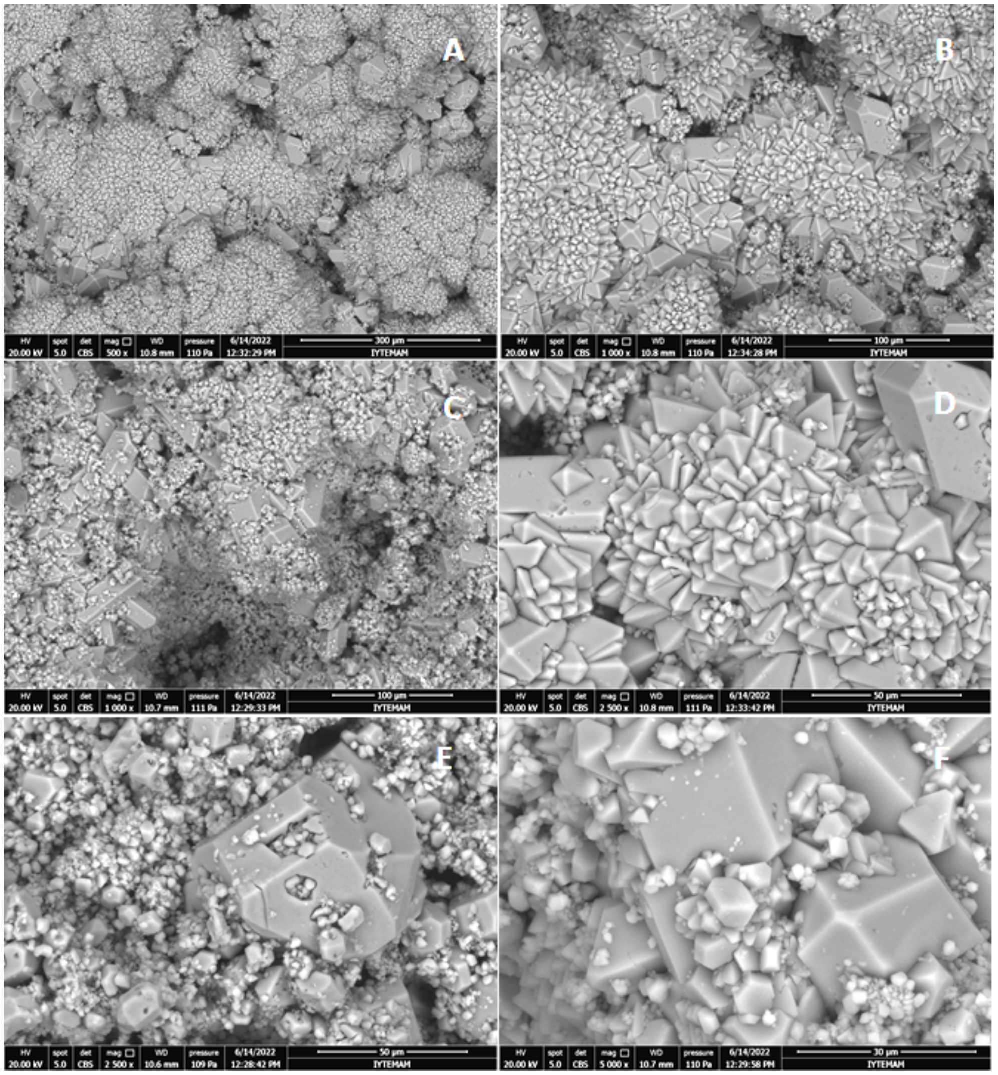

Figure S10. Surface morphology of the AZ31 plate kept for 21 days in 1.5M OPA, 3wt% Mg nitrate solution saturated with Mg: A) 500X, B) 500X, C) 2000X, D) 5000X, E) 5000X, F) 10000X.

Table S12. Phase composition deposited on the surface of AZ31 plate kept for 21 days in 1.5M OPA solution containing 3wt% Mg nitrate saturated with Mg.

Surface morphology	EDX spectroscopy (weight and atomic ratio)
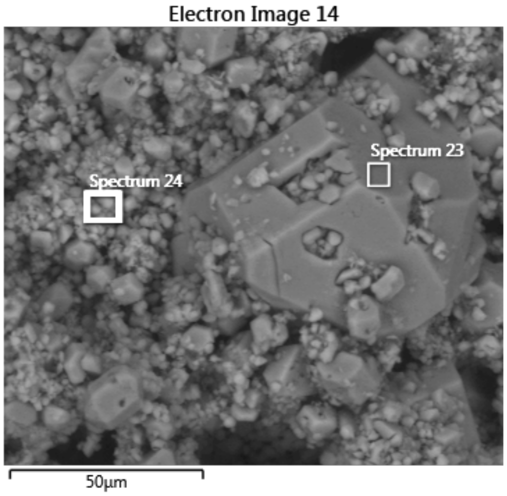	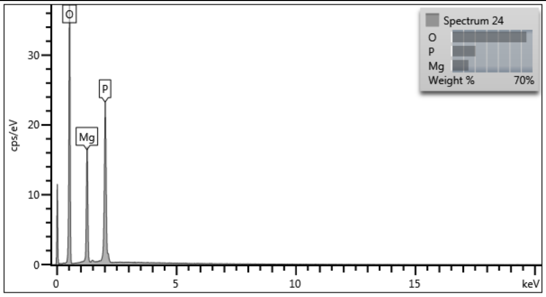 Mg/P/O=11/12/76

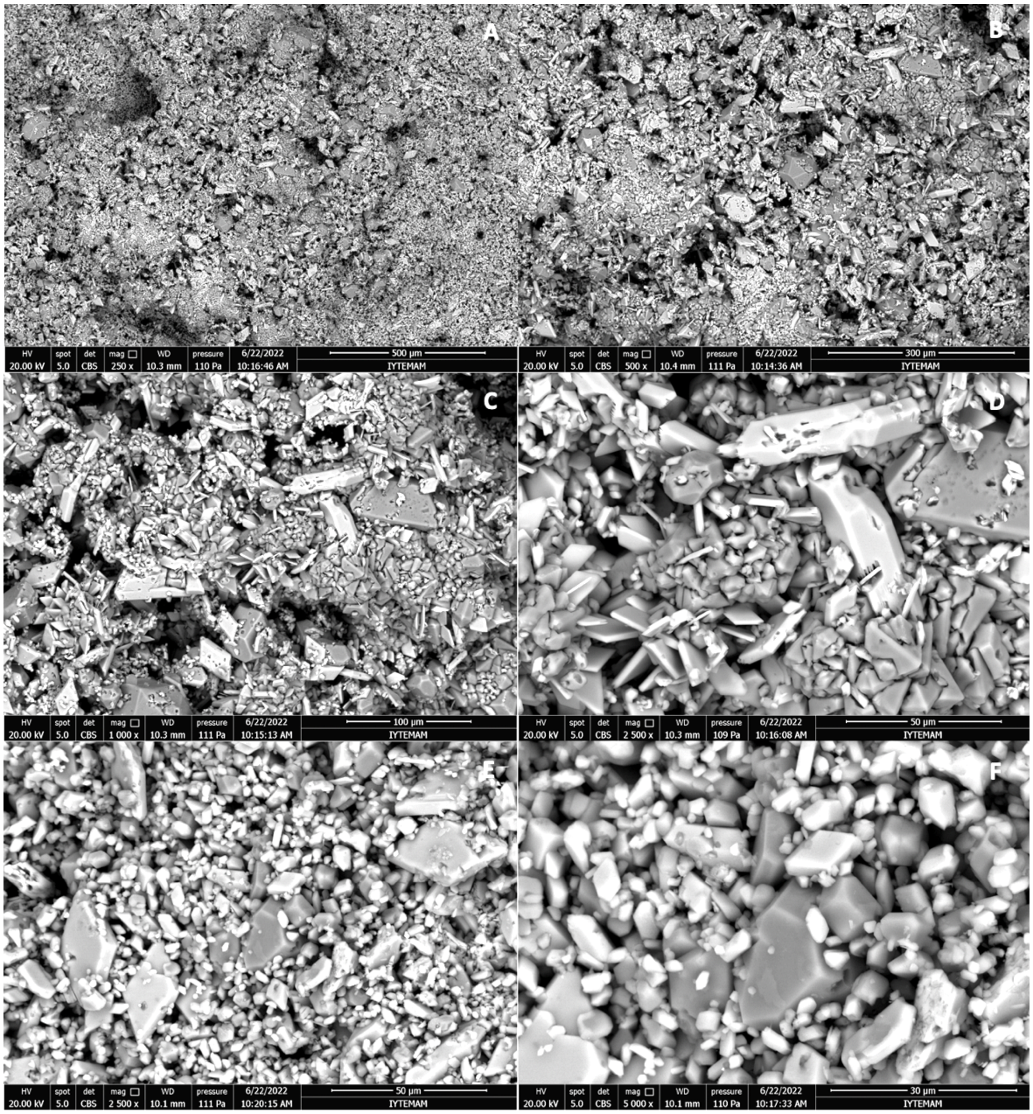

Figure S11. Surface morphology of the AZ31 plate kept for 21 days in 1.5M OPA, 3wt% Ca nitrate solution saturated with Mg: A) 500X, B) 500X, C) 2000X, D) 5000X, E) 5000X, F) 10000X.

Table S13. Phase composition deposited on the surface of AZ31 plate kept for 21 days in 1.5M OPA solution containing 3wt% Ca nitrate saturated with Mg.

Surface morphology	EDX spectroscopy (weight and atomic ratio)
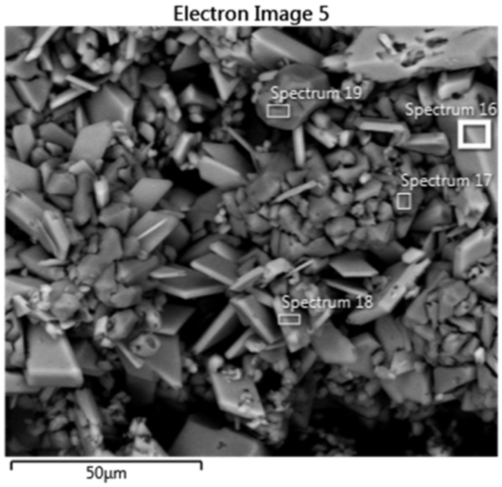	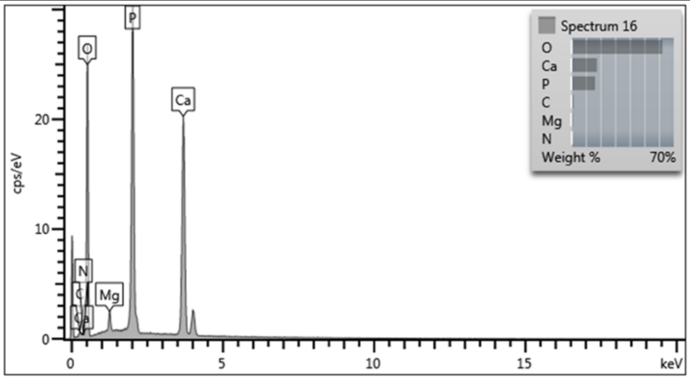 Mg/P/Ca=1/10/8.5 O=76 N=1

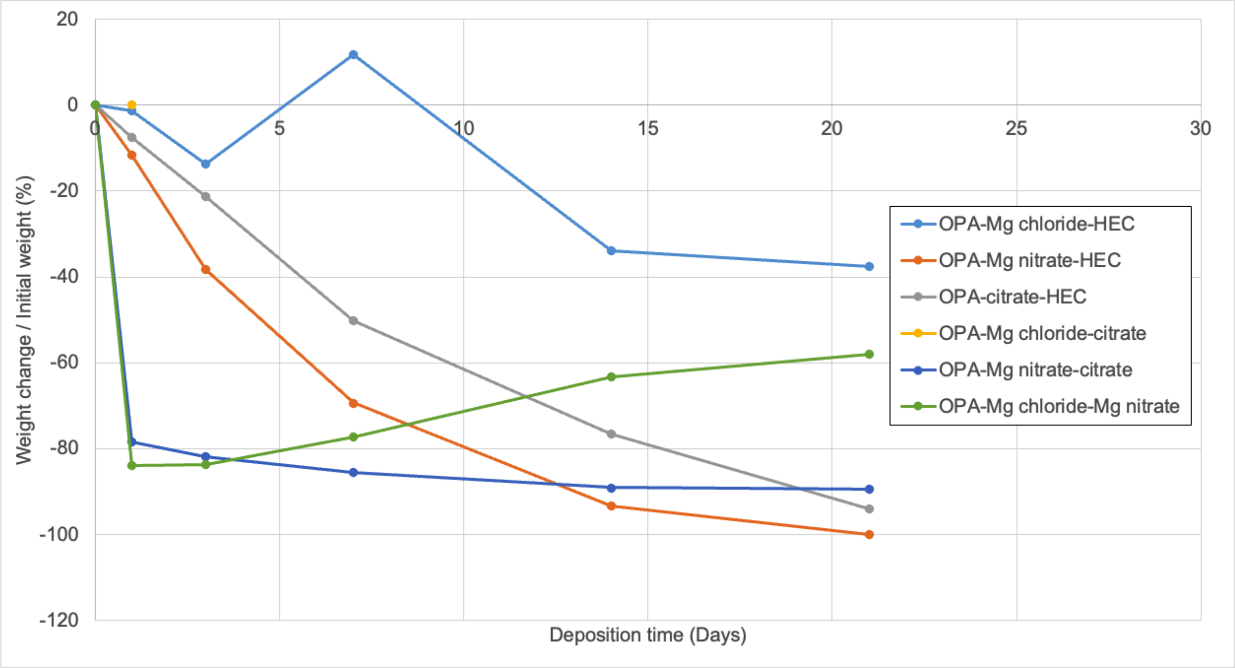

Figure S12. Gravimetric analysis of AZ31 plates immersed in three component solutions for 21 days.

Table S14. Electrochemical test results of AZ31 plates immersed in three component solutions for 21 days.

Sample	Open circuit potential (V)	E_{corr} (V)	I_{corr} (uA)	β_a (mV)	β_c (mV)	Corrosion resistance (ohm)	Corrosion rate mm/y
OPA-Mg chloride-HEC	-1.49	-1.418	7.395	119.6	242.8	4240	0.12718
OPA-Mg chloride-citrate	-1.51	-1.438	10.074	68.8	204.6	2910	0.17325
OPA-Mg chloride-Mg nitrate	-1.53	-1.448	14.903	70.8	217.2	1772	0.25630
OPA-citrate-HEC	-1.50	-1.470	14.928	51.1	188.4	303	0.25673
OPA-Mg nitrate-citrate	-1.50	-1.447	18.942	87.4	233.3	1278	0.32577
OPA-Mg nitrate-HEC	N/A	N/A	N/A	N/A	N/A	N/A	N/A

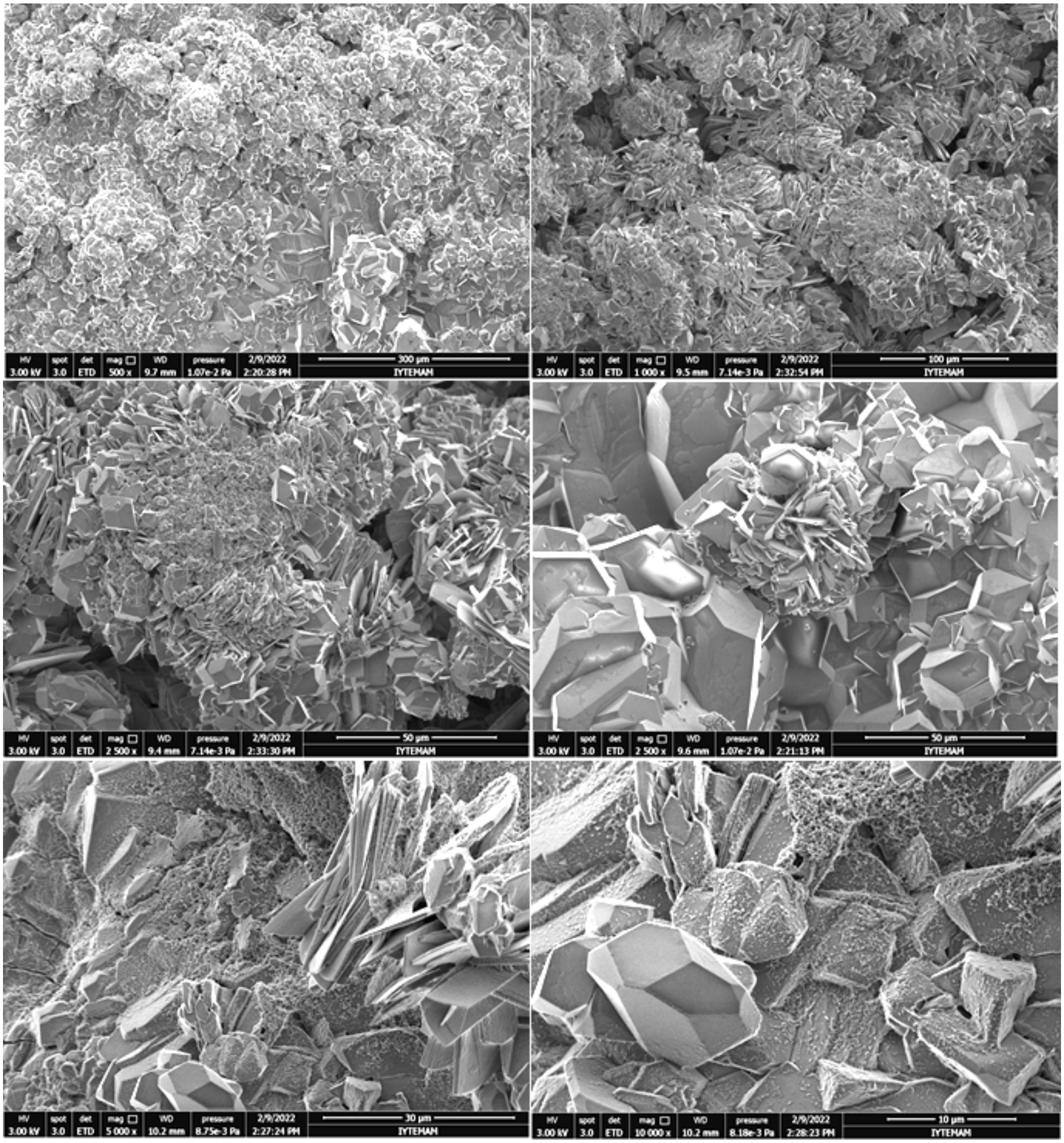

Figure S13. The phase structure accumulated on the surface of the AZ31 plate kept in a solution containing OPA, $MgCl_2$, and HEC for 21 days.

Table S15. Composition of the phase deposited on the surface of the AZ31 plate kept in the solution containing OPA, $MgCl_2$ and HEC for 21 days.

<table>
<tr><td>Surface Morphology</td><td>EDX spectroscopy (weight and atomic ratio)</td></tr>
<tr><td rowspan="3">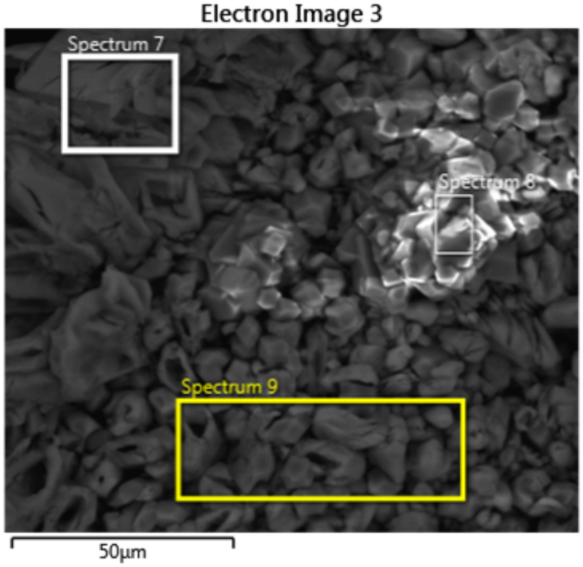
</td><td>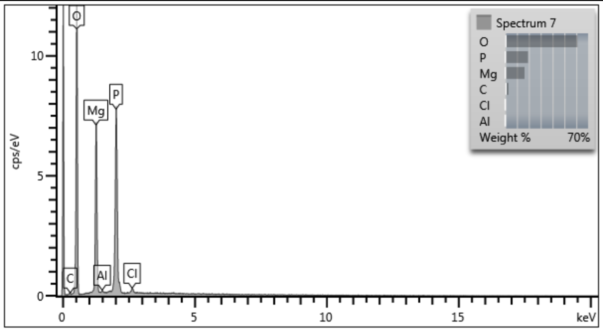

Mg/P/Ca=13/12/0 O=71</td></tr>
<tr><td>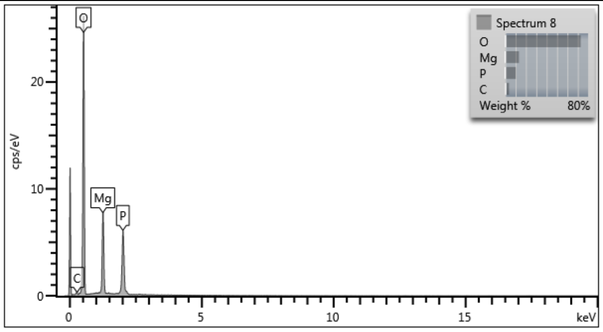

Mg/P/Ca=10/6/0 O=79</td></tr>
<tr><td>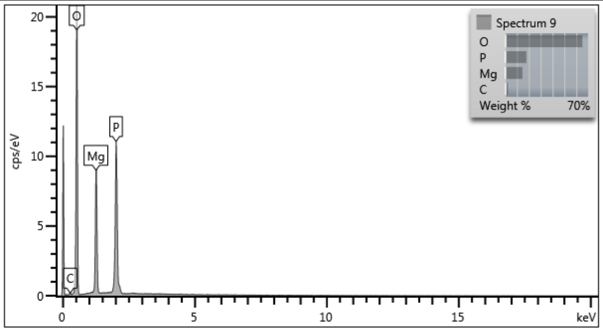

Mg/P/Ca=13/12/0 O=71</td></tr>
</table>

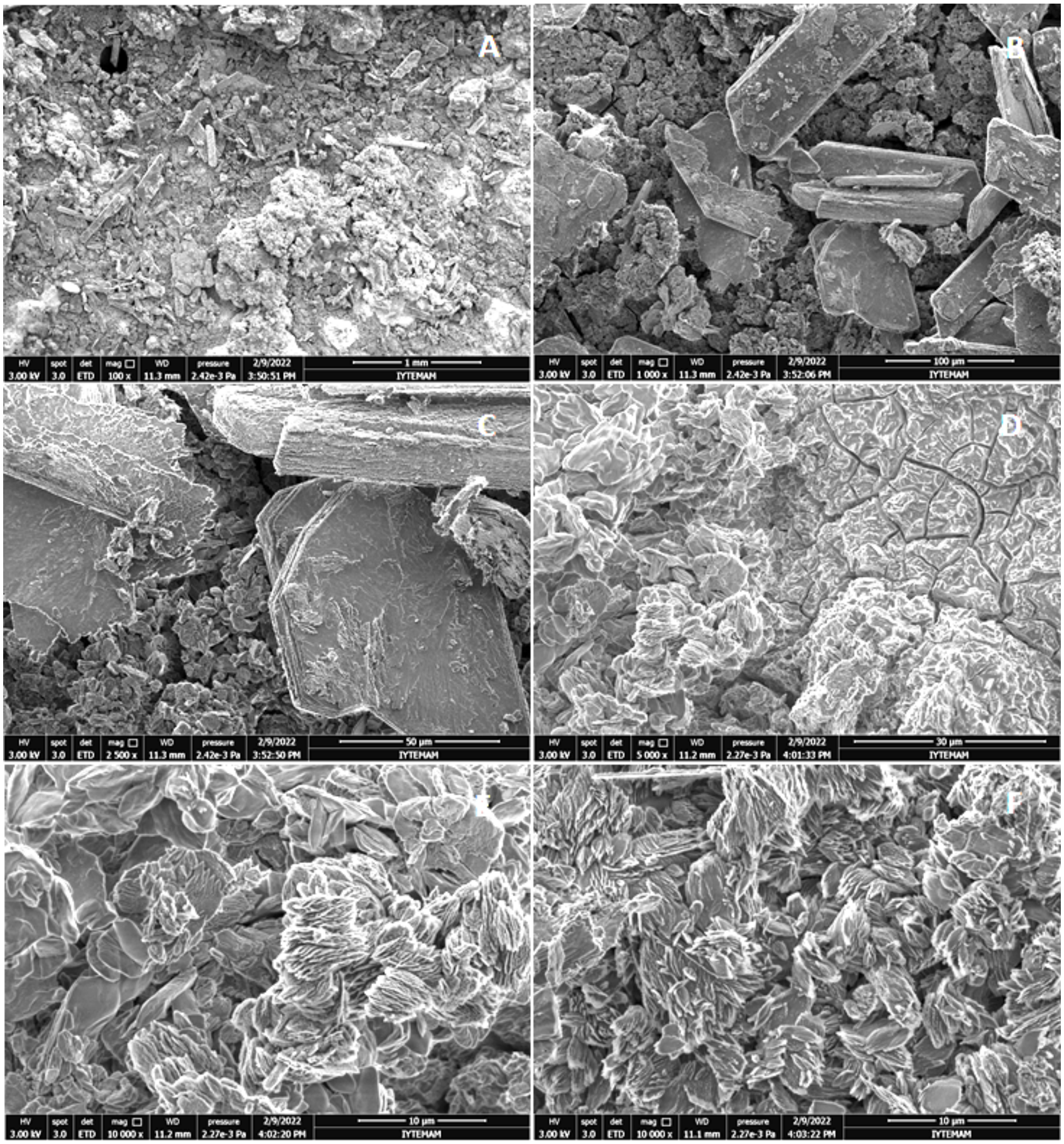

Figure S14. Surface morphology of the deposited layer on the surface of the AZ31 plate kept in a solution containing OPA, Citrate, and $MgCl_2$ for 21 days.

Table S16. Composition of the phase deposited on the surface of the AZ31 plate kept in the solution containing OPA, Citrate and $MgCl_2$ for 21 days.

Surface Morphology	EDX spectroscopy (weight and atomic ratio)
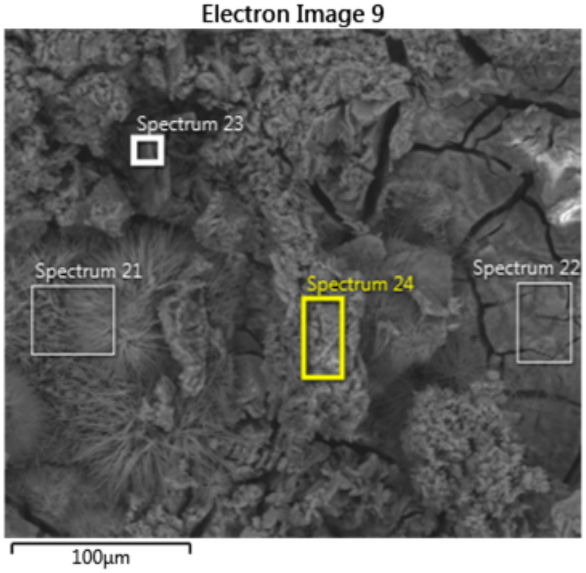	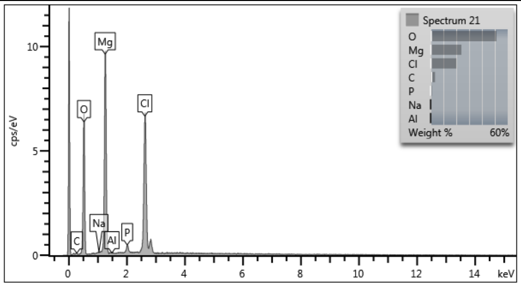 Mg/P/Ca=19/1/0 O=63 Cl=11
	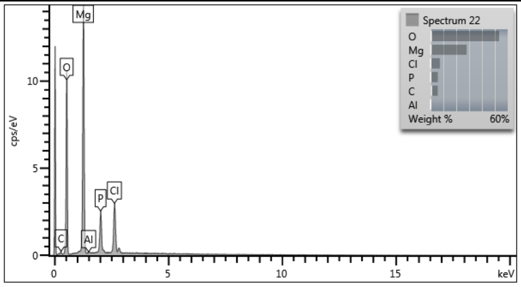 Mg/P/Ca=22/3/0 O=62 Cl=4
	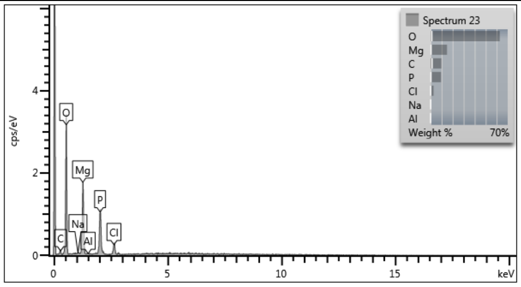 Mg/P/Ca=11/5/0 O=68 Cl=1
	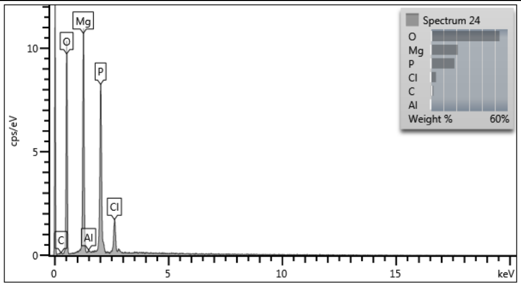 Mg/P/Ca=17/12/0 O=65 Cl=2

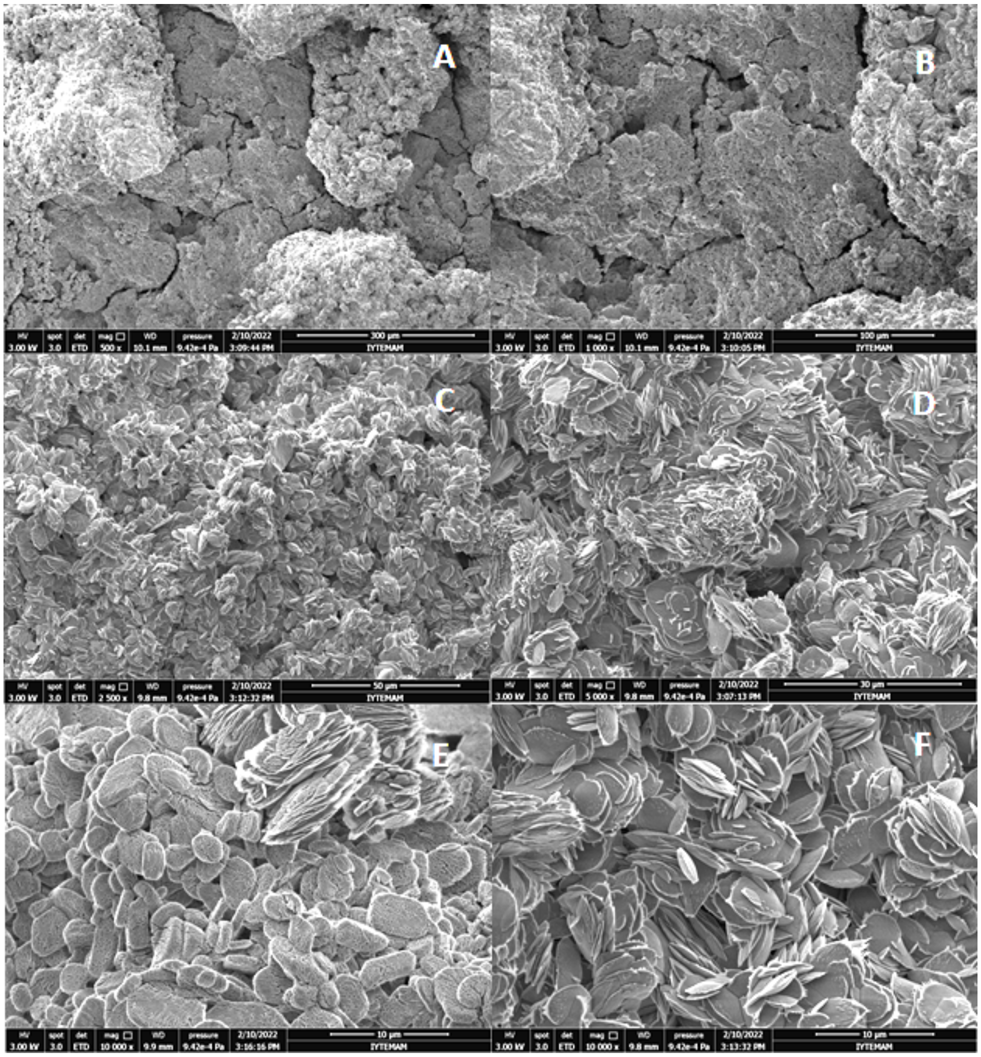

Figure S15. Surface morphology of the deposited layer on the surface of the AZ31 plate kept in a solution containing OPA, $MgNO_3$, and Citrate for 21 days.

Table S17. Composition of the phase deposited on the surface of the AZ31 plate kept in the solution containing OPA, $MgNO_3$, and Citrate for 21 days.

Surface Morphology	EDX spectroscopy (weight and atomic ratio)
	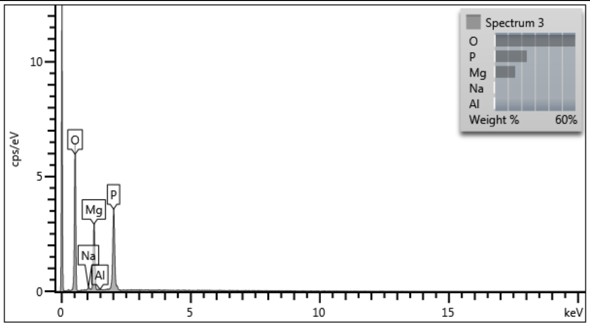 Mg/P/Ca=12/15/0 O=72

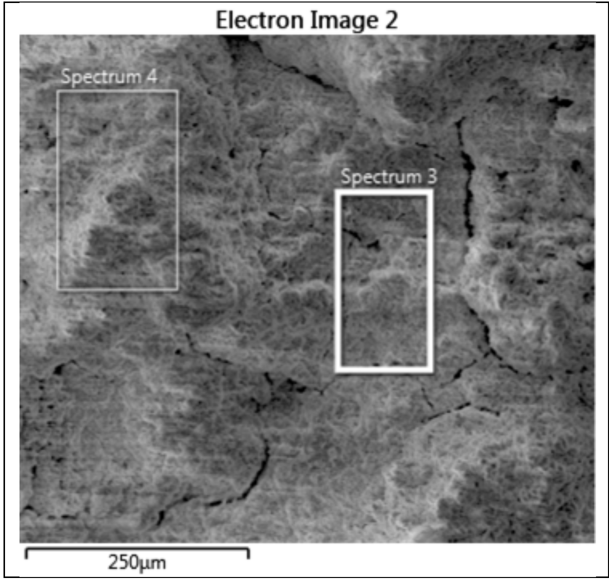

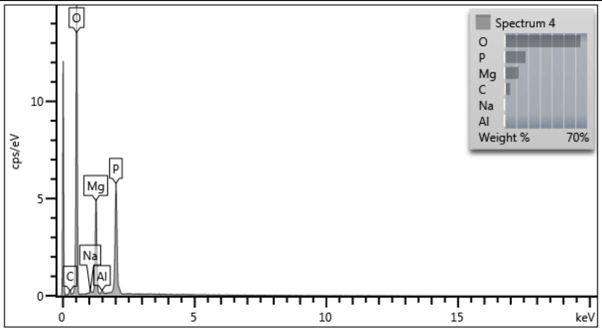

Mg/P/Ca=9/10/0 O=73

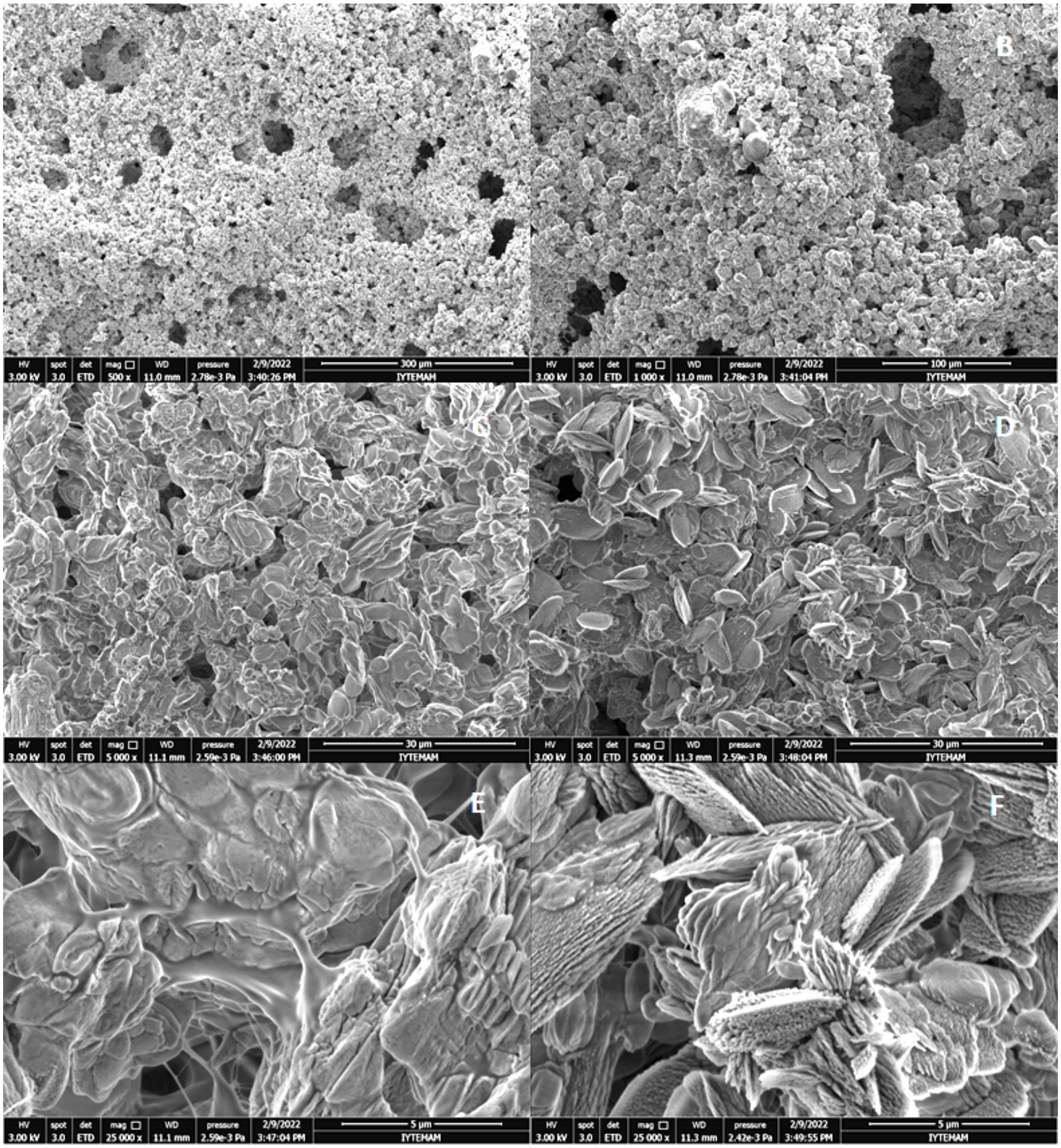

Figure S16. Surface morphology of the deposited layer on the surface of the AZ31 plate kept in a solution containing OPA, Citrate, and HEC for 21 days.

Table S18. Composition of the phase deposited on the surface of the AZ31 plate kept in the solution containing OPA, Citrate and HEC for 21 days.

<table>
<tr><td>Surface Morphology</td><td>EDX spectroscopy (weight and atomic ratio)</td></tr>
<tr><td rowspan="3">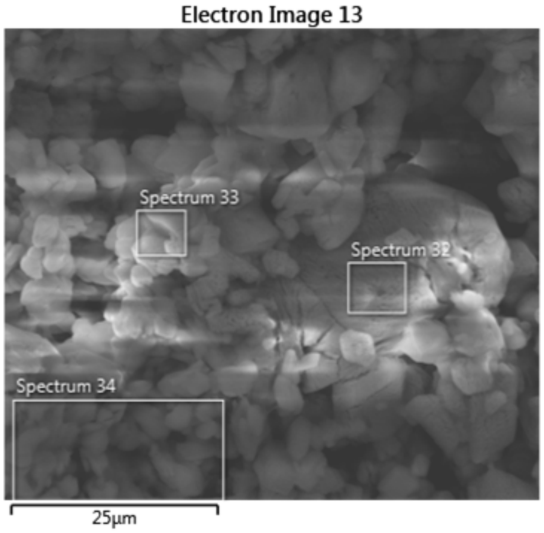
</td><td>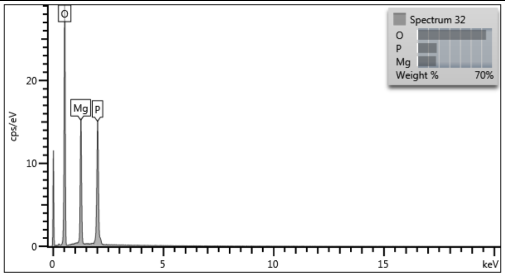

Mg/P/Ca=13/11/0 O=75</td></tr>
<tr><td>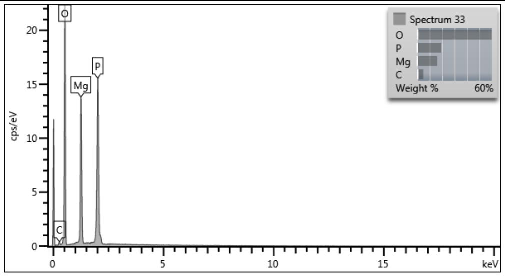

Mg/P/Ca=12/11/0 O=69</td></tr>
<tr><td>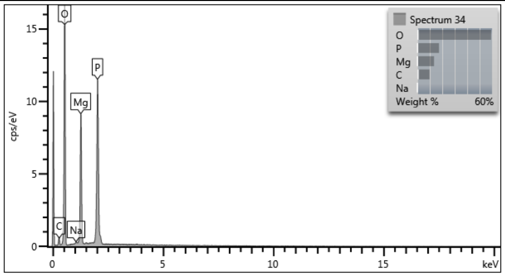

Mg/P/Ca=10/10/0 O=66</td></tr>
</table>

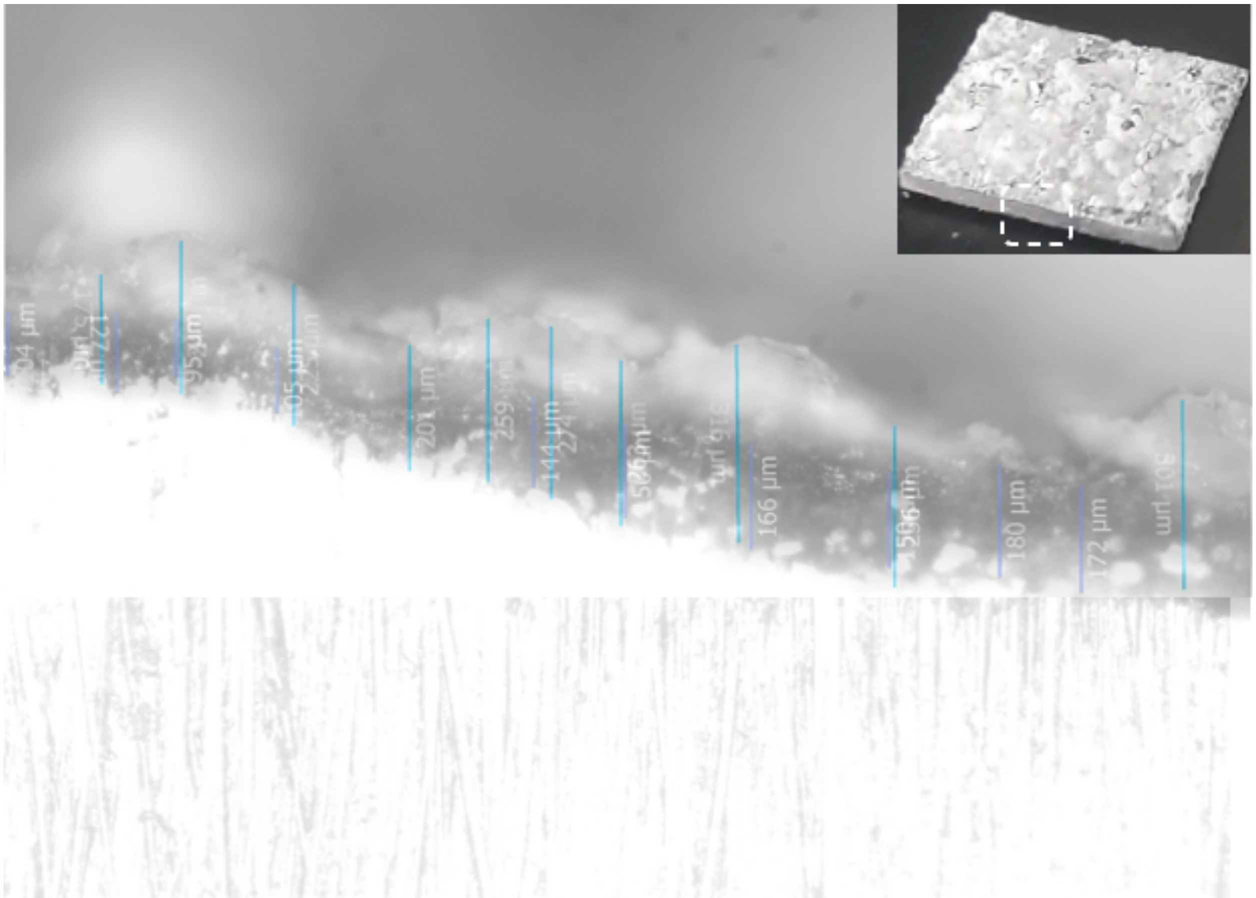

Figure S17. Coating thickness of RTC sample measured under 40X magnification.

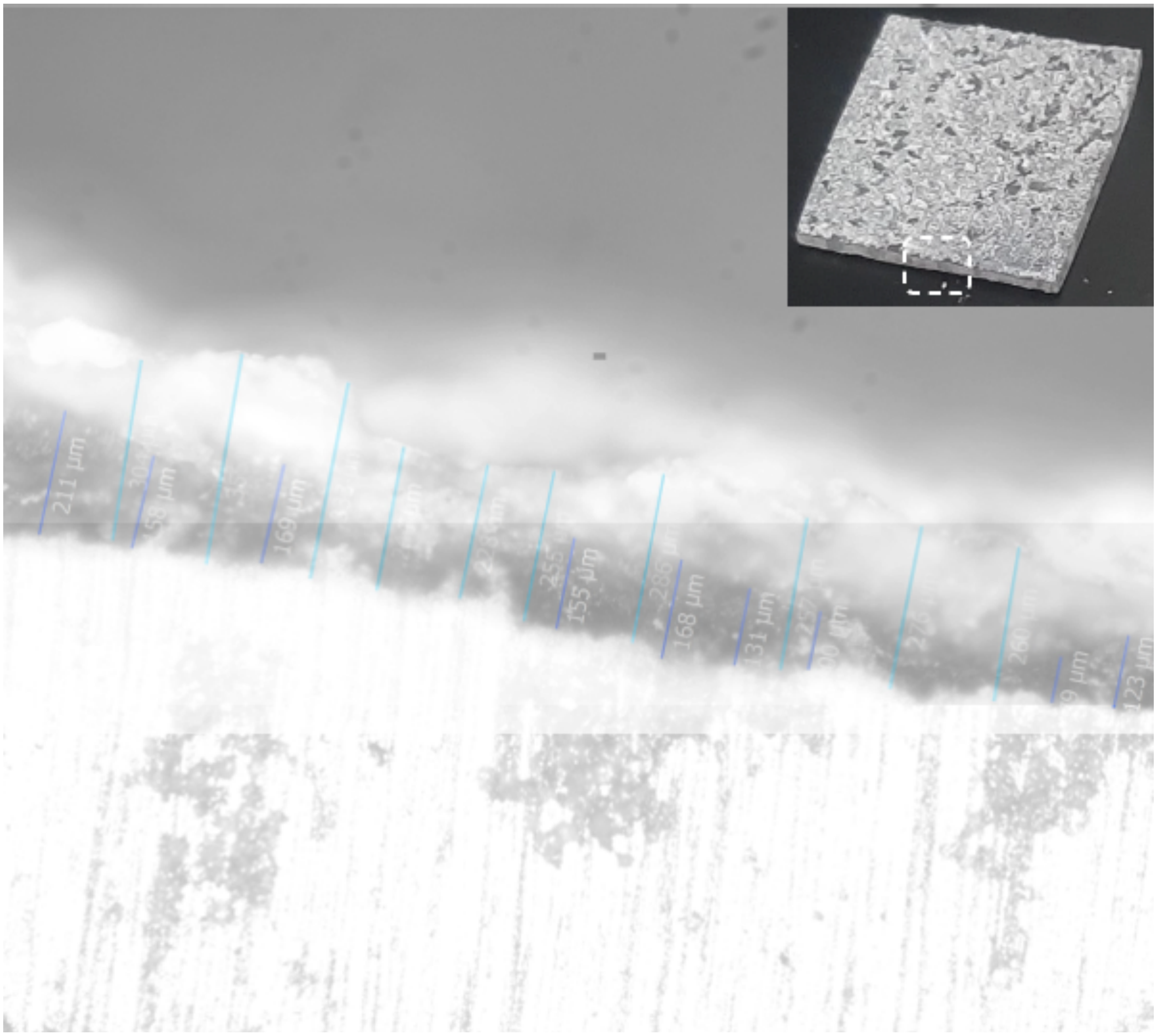

Figure S18. Coating thickness of HTC sample measured under 40X magnification.

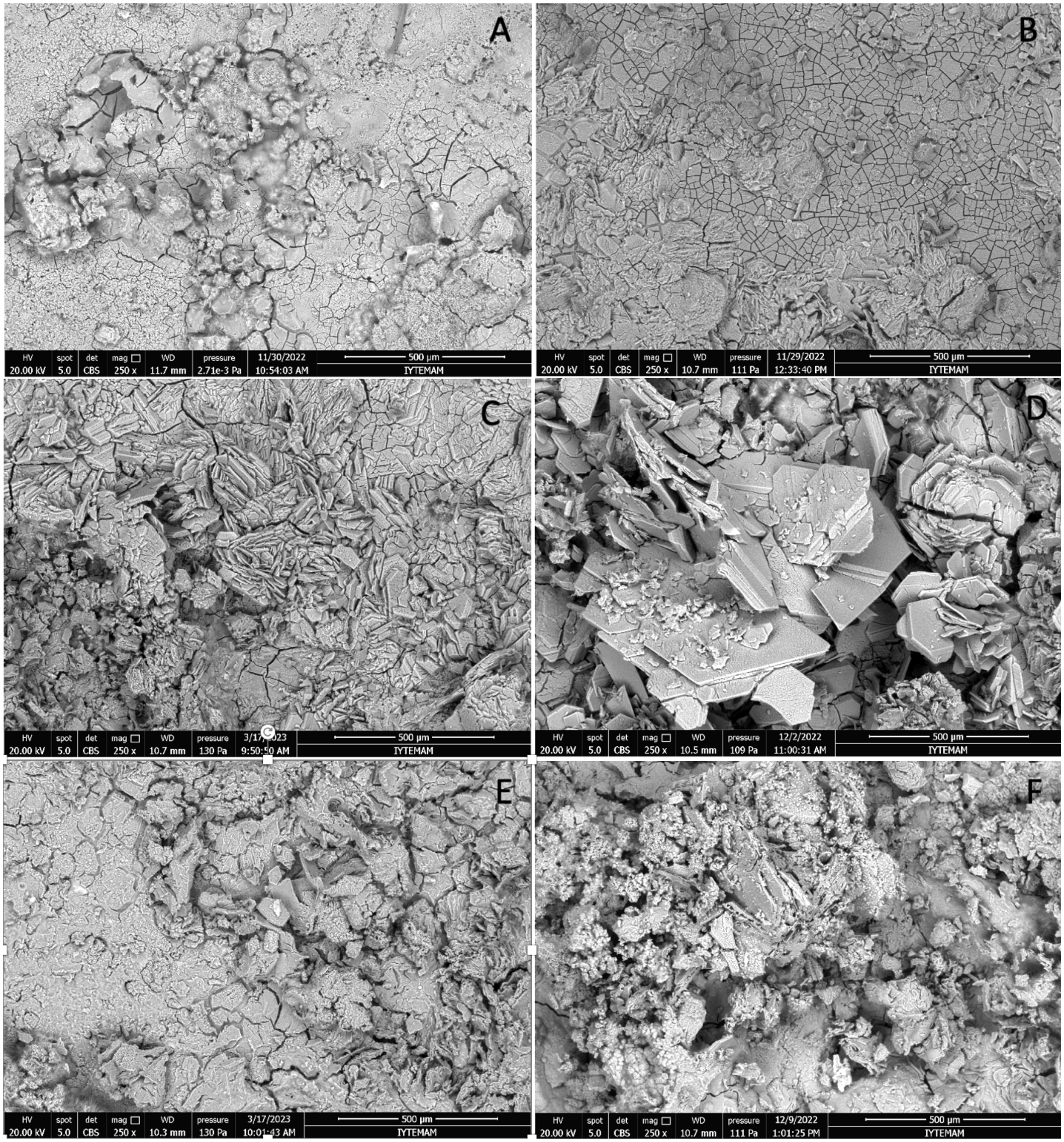

Figure S19. Surface morphologies of RTC samples upon immersion in 3.5% NaCl solution at various immersion periods: no immersion (A), 1 day immersion (B), 3 day immersion (C), 7 day immersion (D), 14 day immersion (E), 21 day immersion (F).

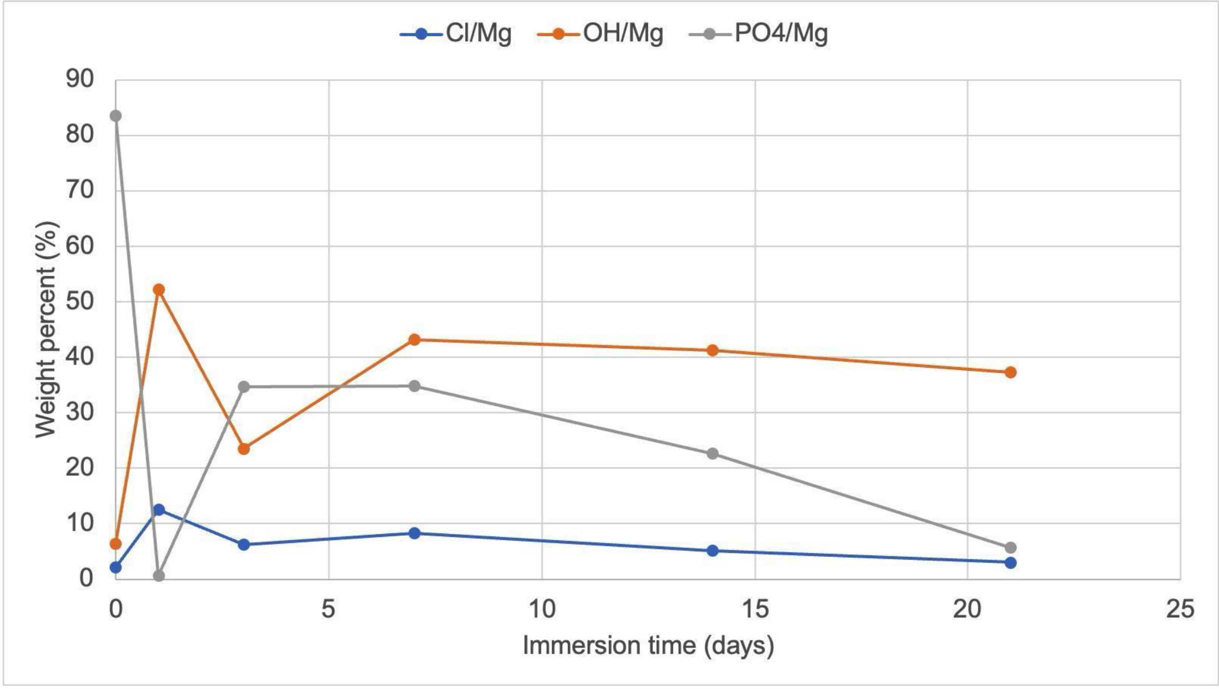

Figure S20. Variations in the anion/cation ratio of RTC samples upon immersion in 3.5% NaCl solution.

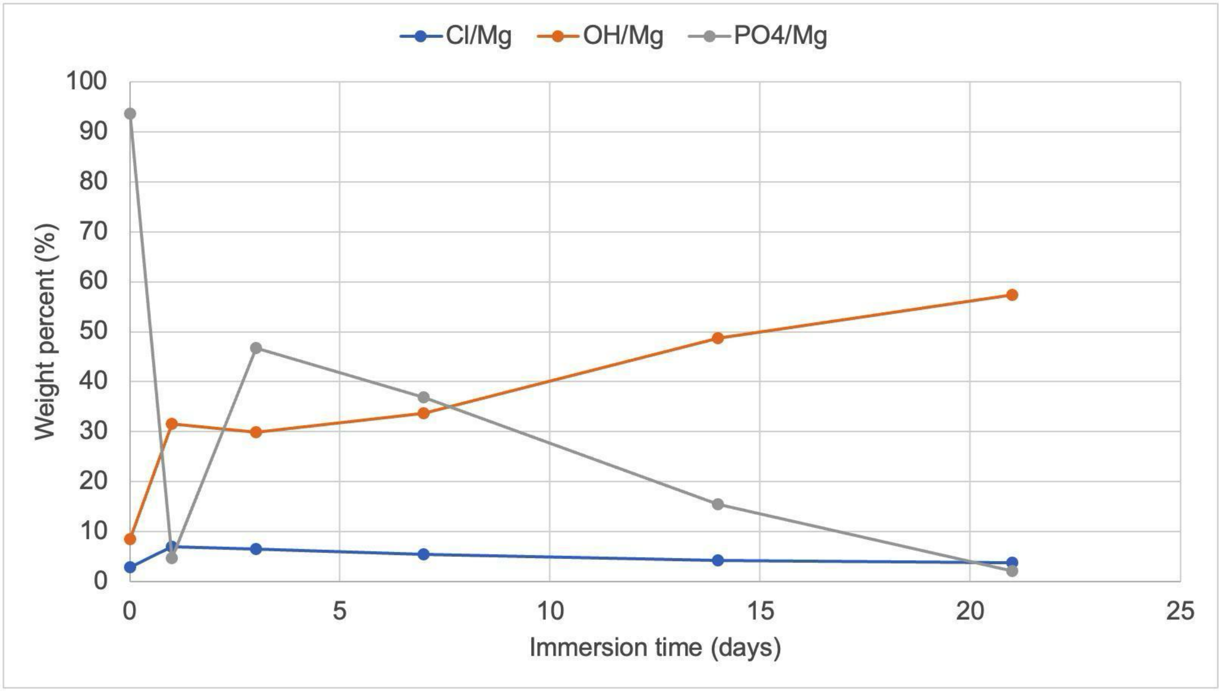

Figure S21. Variations in the anion/cation ratio of RTC samples upon immersion in 3.5% NaCl solution.

The evolution of the sample surfaces is also reflected in the corrosion behavior. In the open circuit voltage and polarization curves of the samples dried at room temperature, given in Figures S22 and S23, a significant difference is seen between the first samples and those kept in solution. While the samples not kept in salt solution reached an open circuit voltage of -1.58 V, those kept for one day reached -1.56 V, and those kept for a longer time reached -

1.55 V. Since there was little formation on the surface on the first day, the samples showed this value immediately, while it took time for the others to reach equilibrium. A similar trend is seen in the polarization curves. The first samples showed high current and voltage absolute values. Over time, the curves shifted to the left and up, and a shift to high current was observed again in 21 days. The excessive deterioration of the samples due to erosion in the long term and the loss of surface homogeneity caused low performance in the long term. Sample pieces containing too many cavities to be tested were also obtained.

The first samples showed high corrosion rates. It is thought that the open points on the surface due to bubbles have a short circuit effect. The low corrosion rate of the samples on the first day also shows that oxychloride formations have a barrier effect. Corrosion resistance showed fluctuations, decreased on the 7th day, increased on the 14th day and decreased again on the 21st day. The surface composition also showed sudden changes during these periods. It is thought that the formed and decomposed phases contributed to the electron transfer during the corrosion measurements and caused a higher corrosion rate than normal.

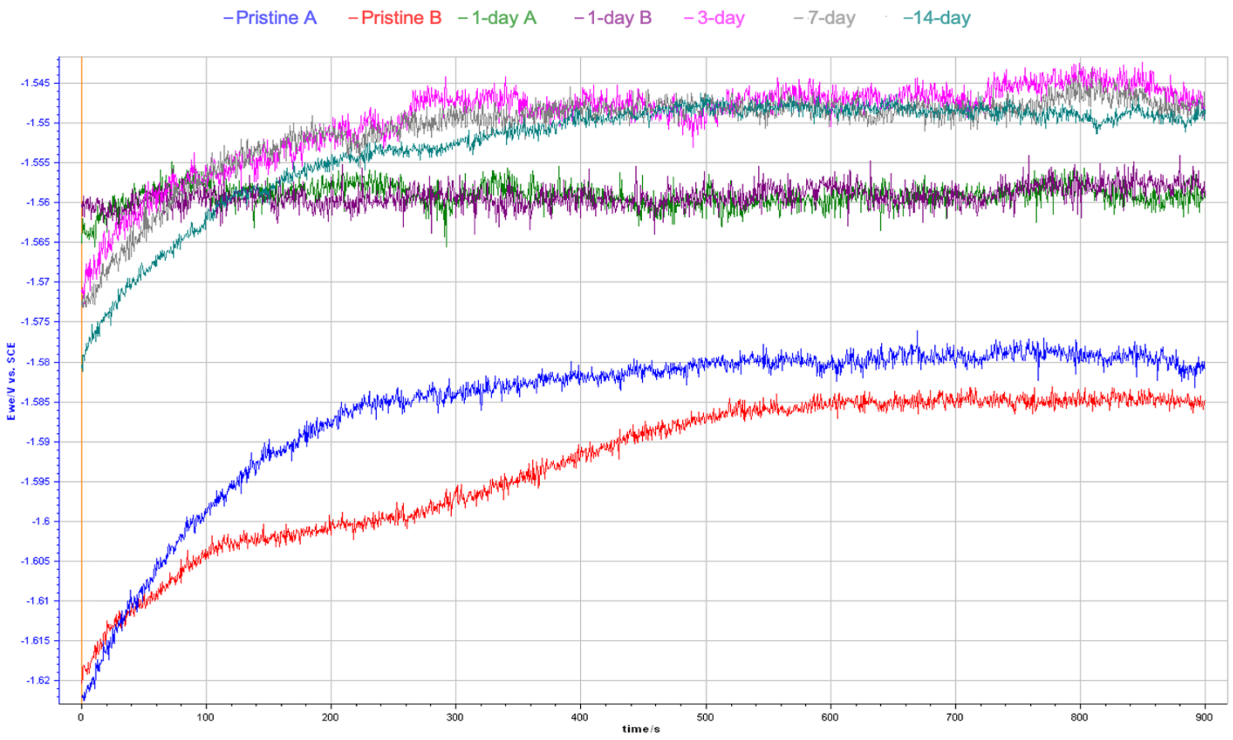

Figure S22. Open circuit potentials of RTC samples immersed in 3.5% NaCl solution for various periods.

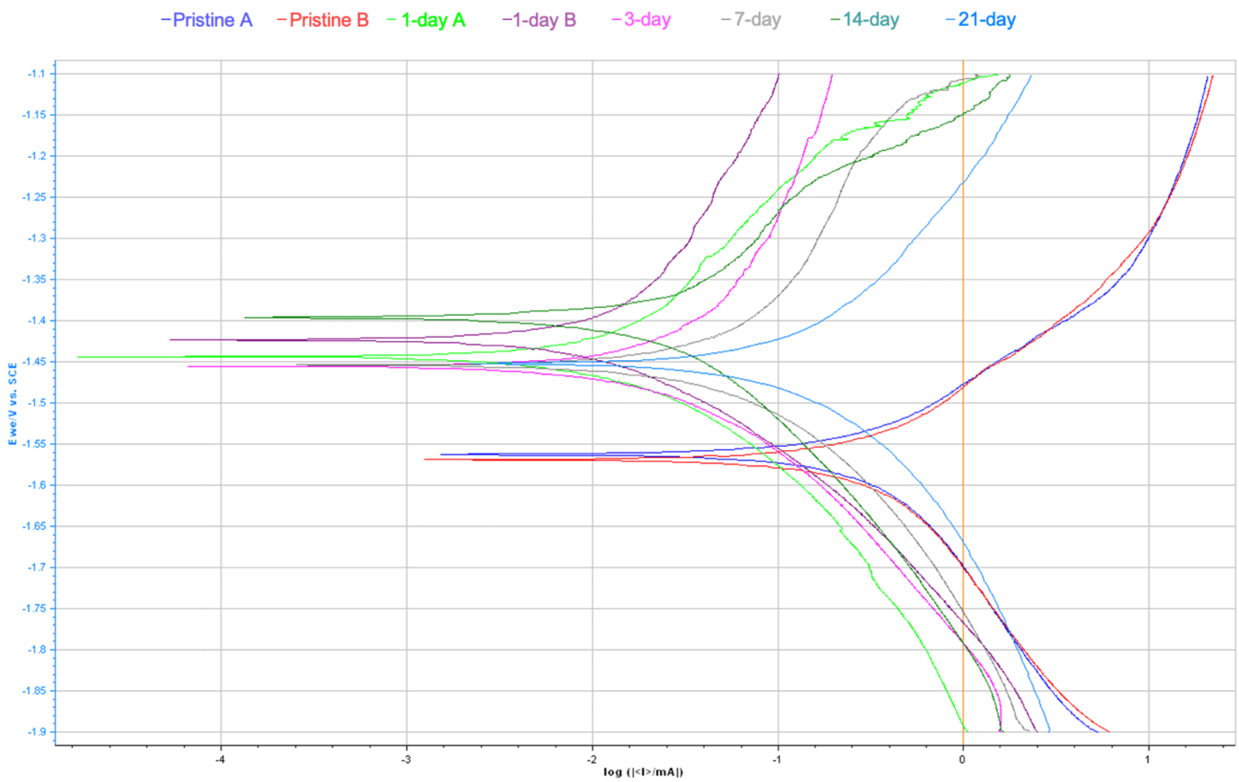

Figure S23. Potential scan curves of RTC samples immersed in 3.5% NaCl solution for various periods.

The main difference in the corrosion tests of coatings containing cross-linked hydrogel is that the first samples have as much corrosion resistance as the others. The open circuit voltages given in Figure S24 were at a similar level (-1.56 V) for all samples. In the polarization curves given in Figure S25, the first samples showed relatively low currents. It is thought that the hydrogel tightens during drying and closes the surface more effectively. The relationship between the waiting time in the solution and the voltage is also valid for this set, the decrease in resistance occurred earlier in the long term. According to the Tafel analysis results, the corrosion rate increased excessively on the 14th day and was measured at a similar level to the other set on the 21st day. It is thought that the reason for this is that the phase decomposition occurred earlier in this set. According to the morphological and compositional analysis, the surface was extremely eroded on the 14th day and continues to erode.

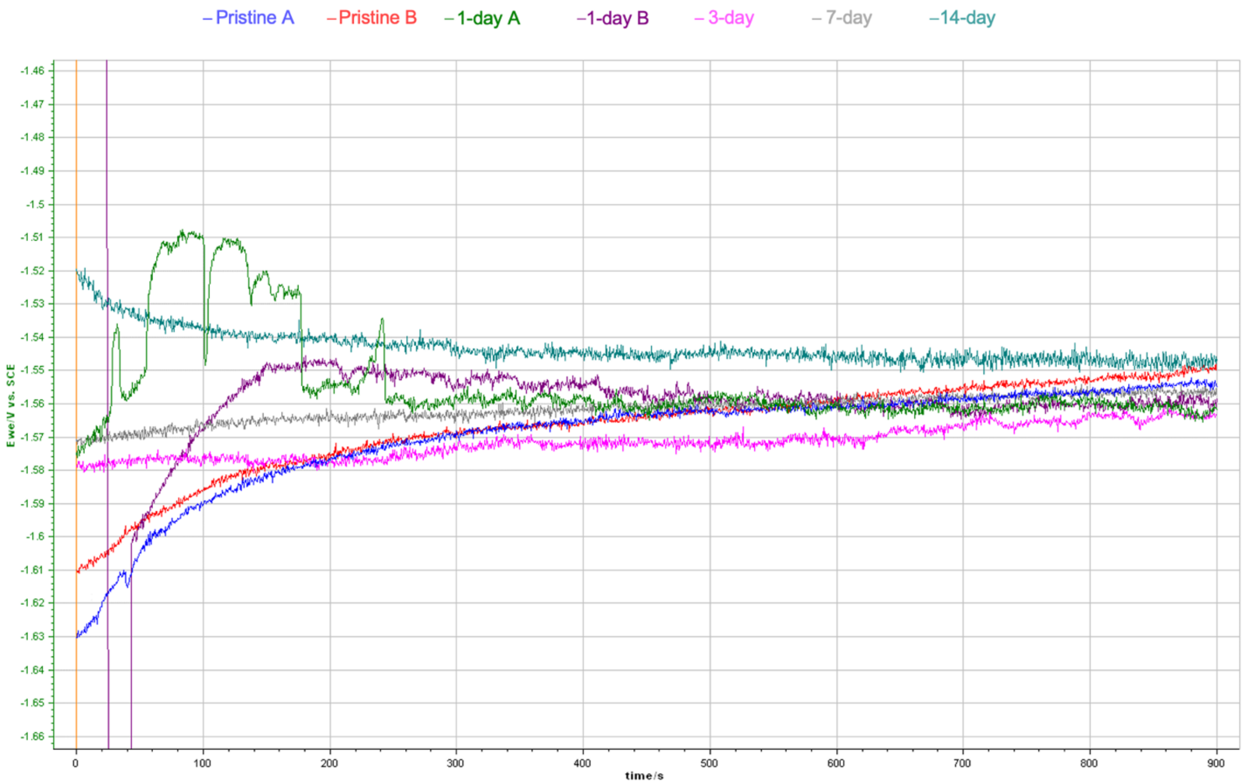

Figure S24. Open circuit potentials of HTC samples immersed in 3.5% NaCl solution for various periods.

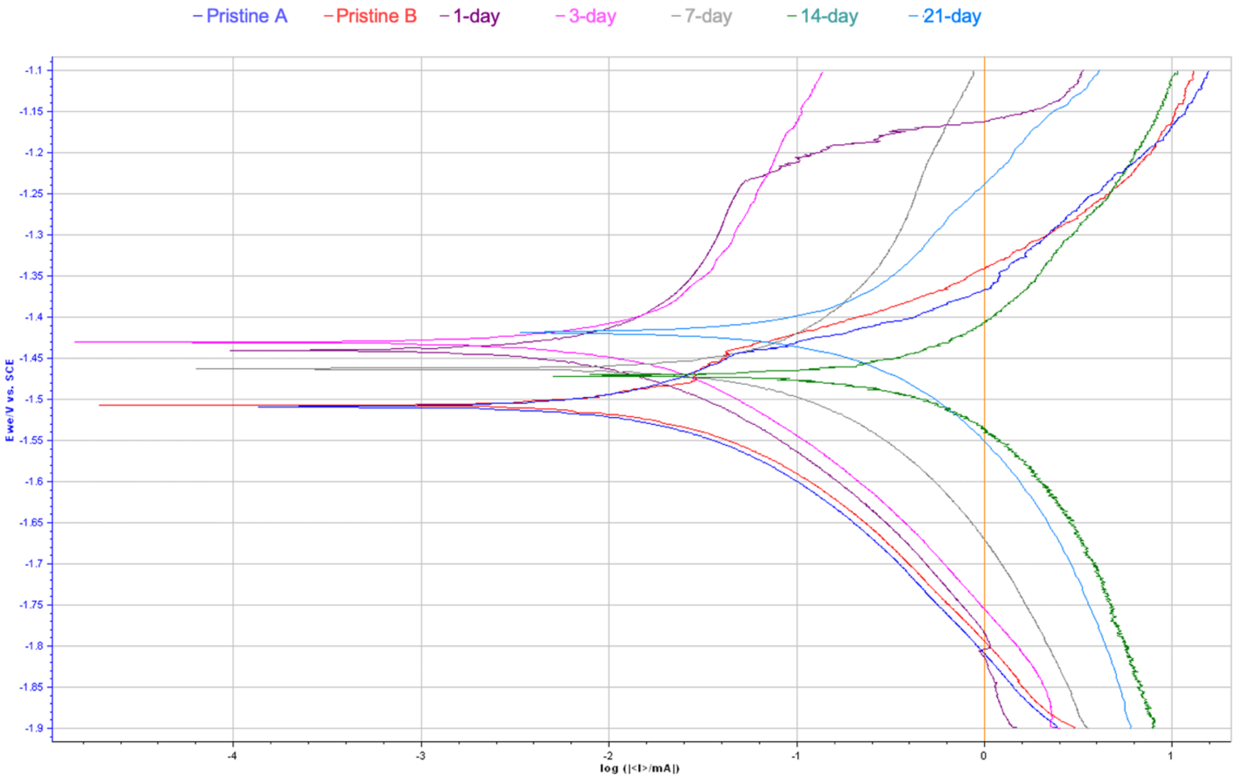

Figure S25. Potential scan curves of HTC samples immersed in 3.5% NaCl solution for various periods.

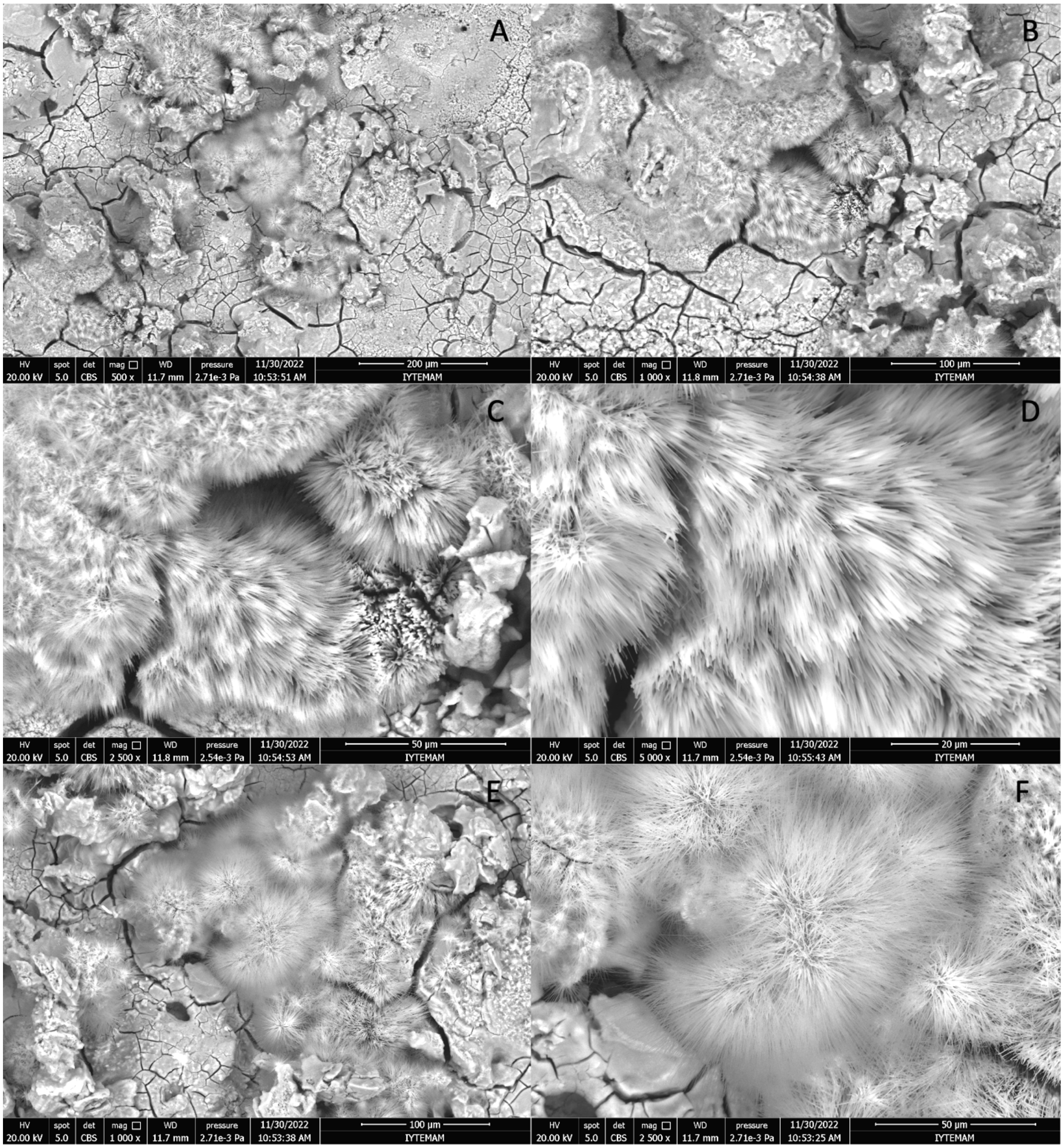

Figure S26. Surface morphologies of HTC samples that were not immersed in 3.5% NaCl solution prior to the corrosion test: wide view (A, B), close views of the degrading surface layers (C, D), close views of heterogeneous Mg oxychloride growth (E, F).

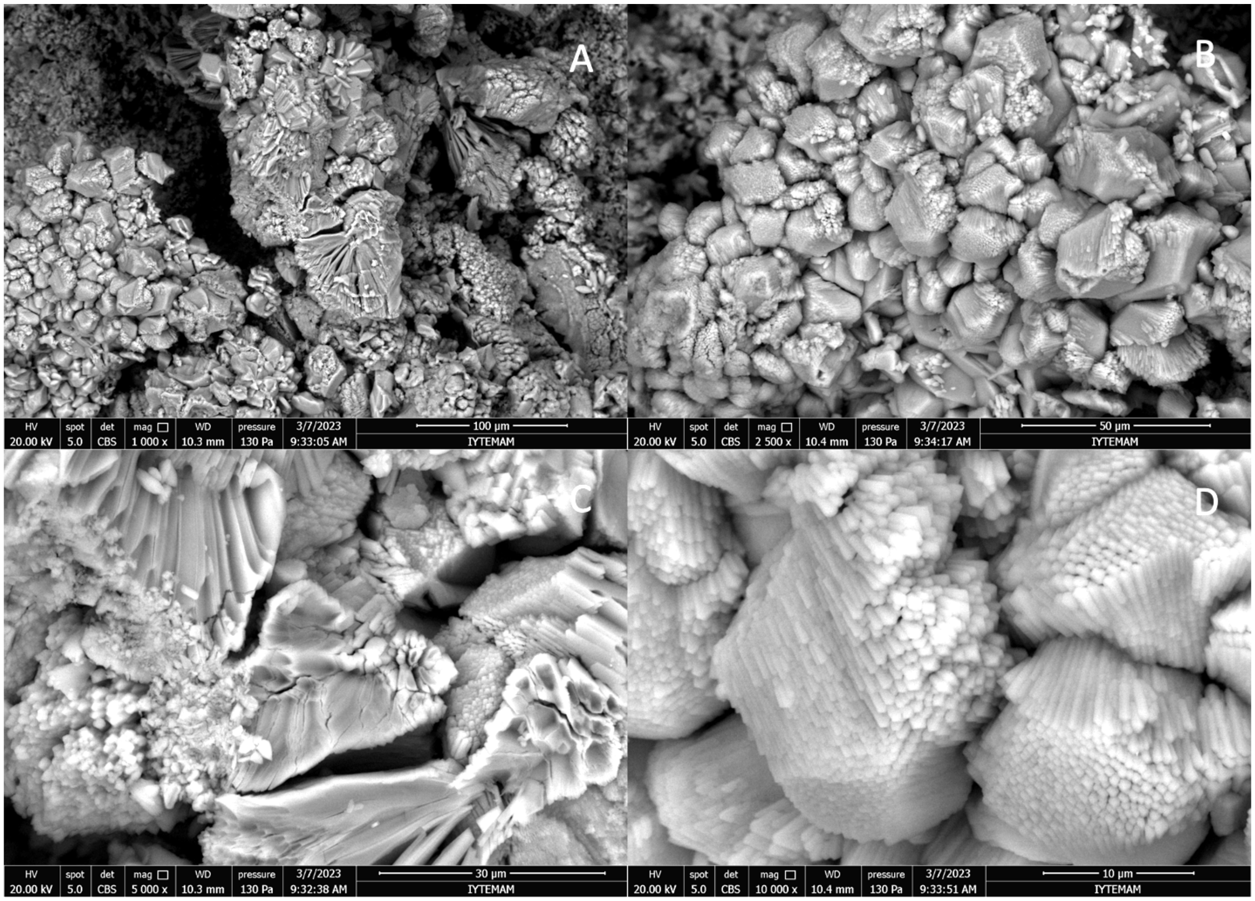

Figure S27. Surface morphologies of RTC samples that were not immersed in 3.5% NaCl solution prior to the corrosion test: wide view (A, B), close views of the degrading surface layers (C, D).

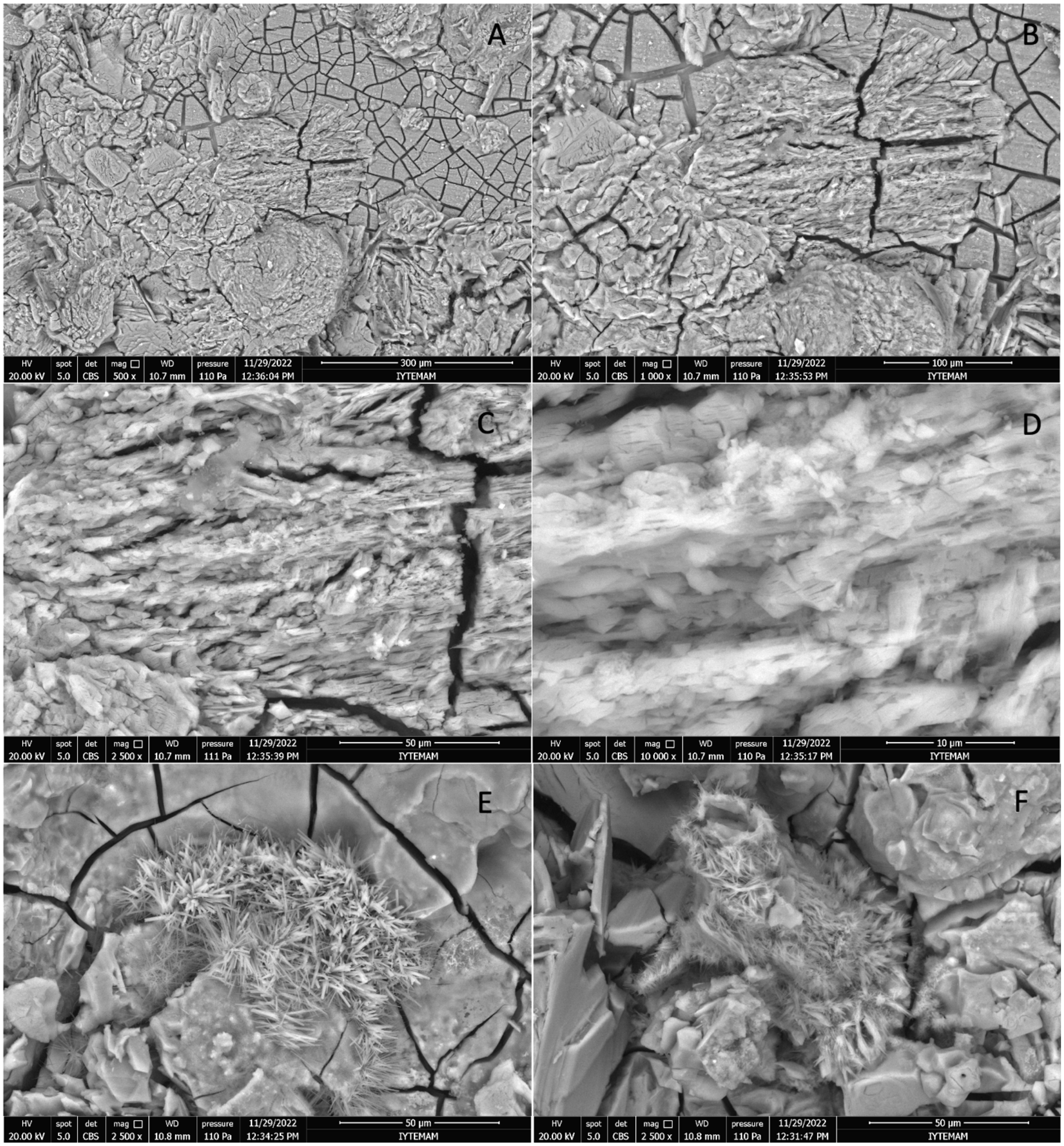

Figure S28. Surface morphologies of HTC samples after 1 day immersion in 3.5% NaCl solution prior to the corrosion test: wide view (A, B), close views of the same surface region (C, D), close views of heterogeneous Mg oxychloride growth (E, F).

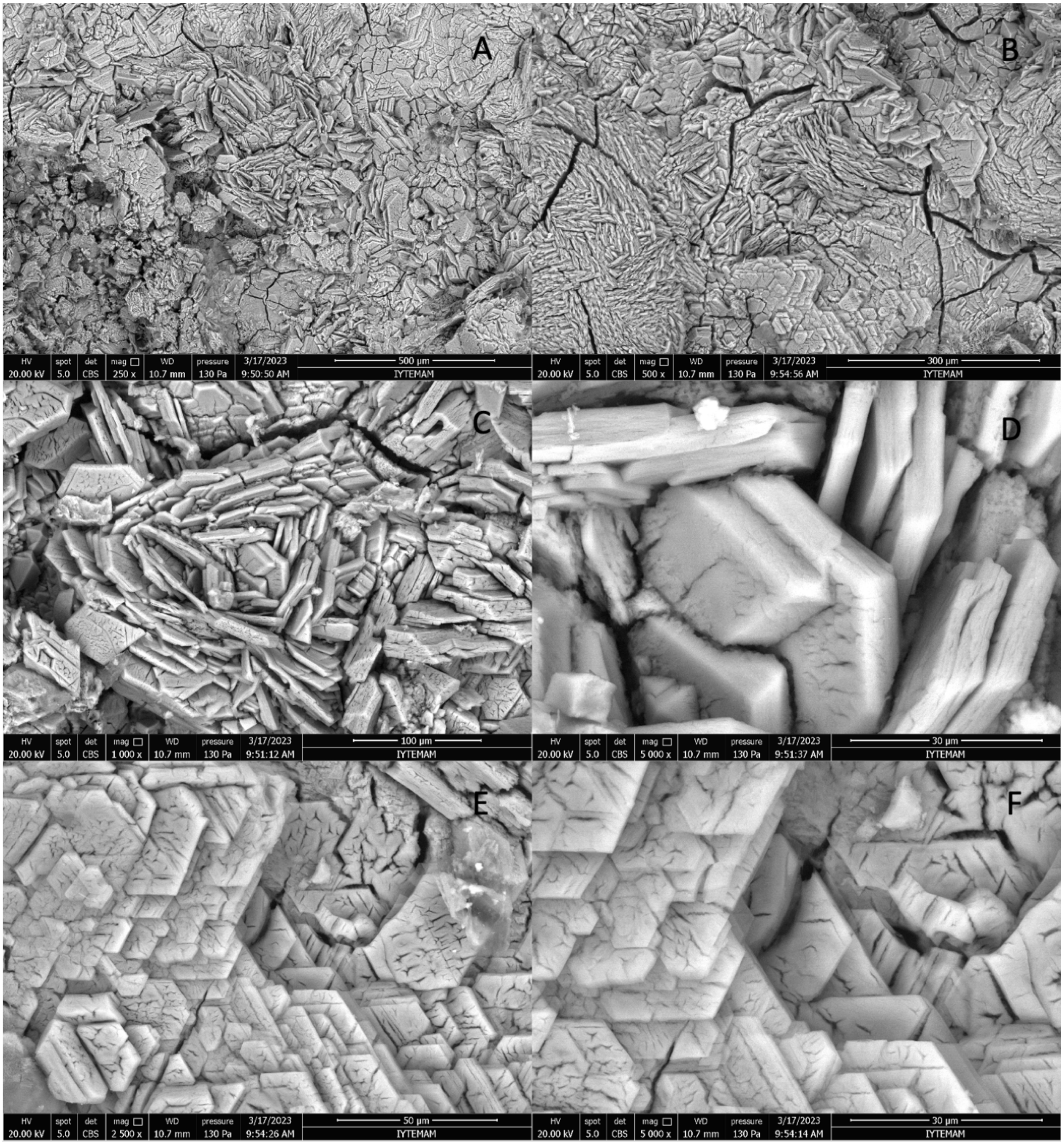

Figure S29. Surface morphologies of HTC samples after 3 day immersion in 3.5% NaCl solution prior to the corrosion test: wide view (A, B), close views of the same region (C, D), close views of terrace-like formations from another region (E, F).

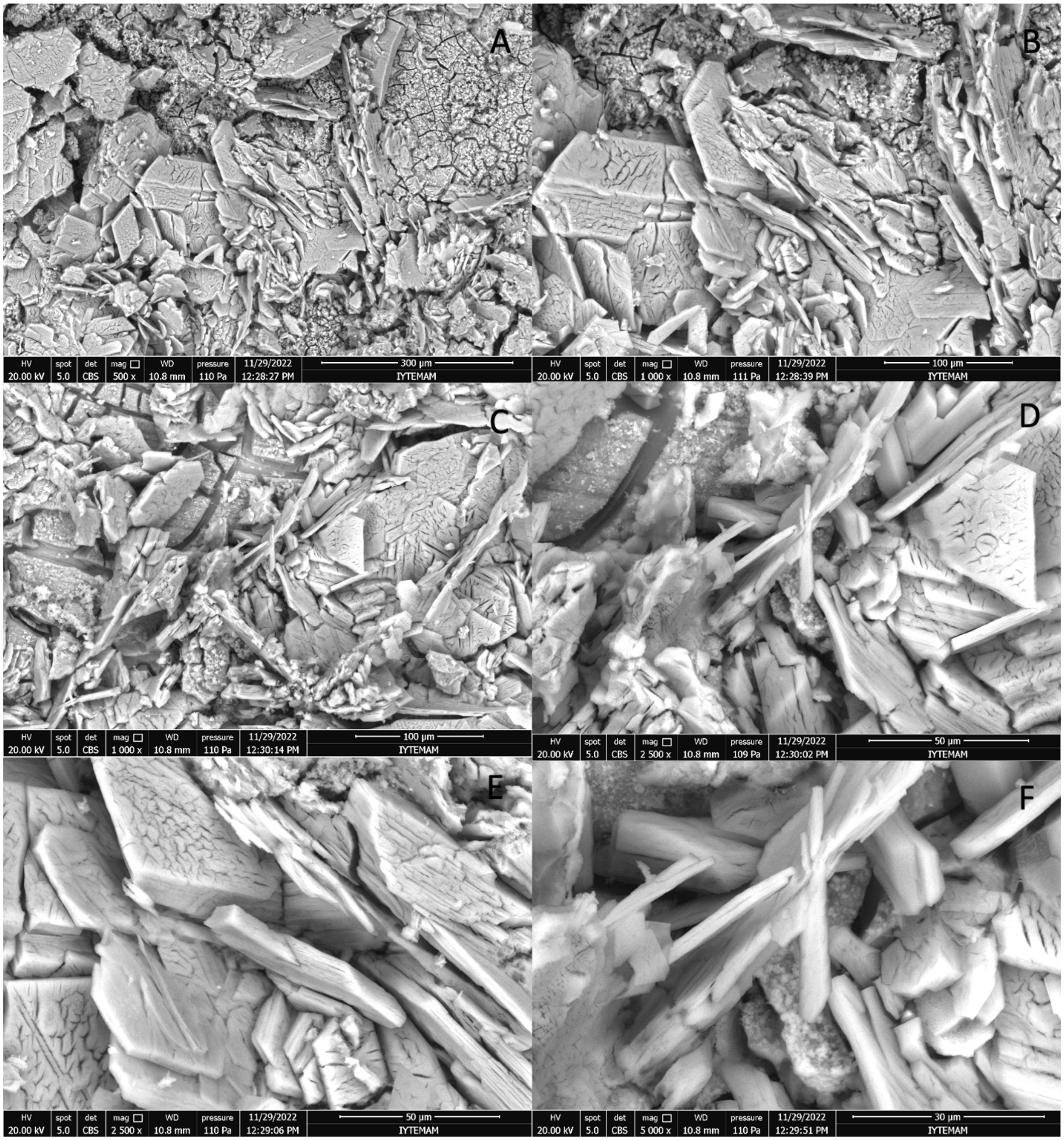

Figure S30. Surface morphologies of RTC samples after 7 day immersion in 3.5% NaCl solution prior to the corrosion test: wide view (A, B), close views of the same region (C, D, E, F).

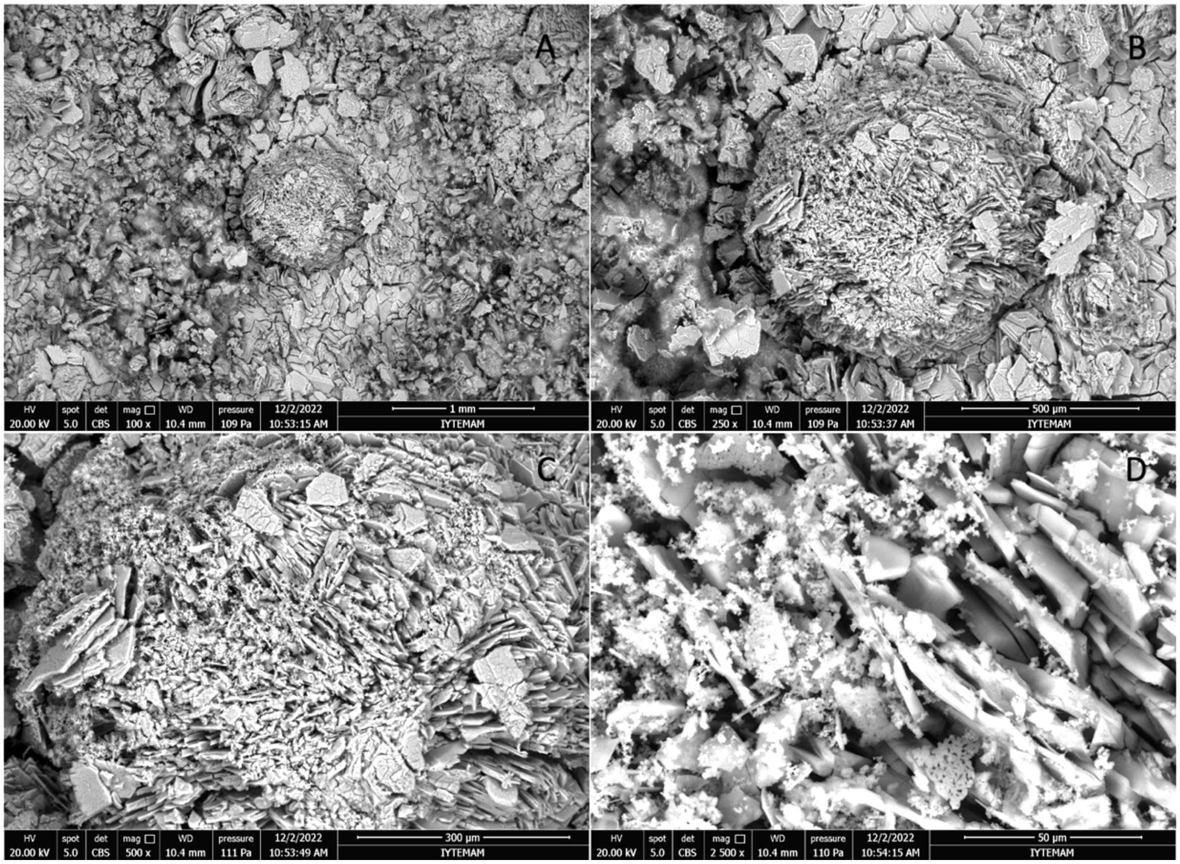

Figure S31. Surface morphologies of HTC samples after 7 day immersion in 3.5% NaCl solution prior to the corrosion test: wide view (A, B), close view of the same region (C, D).

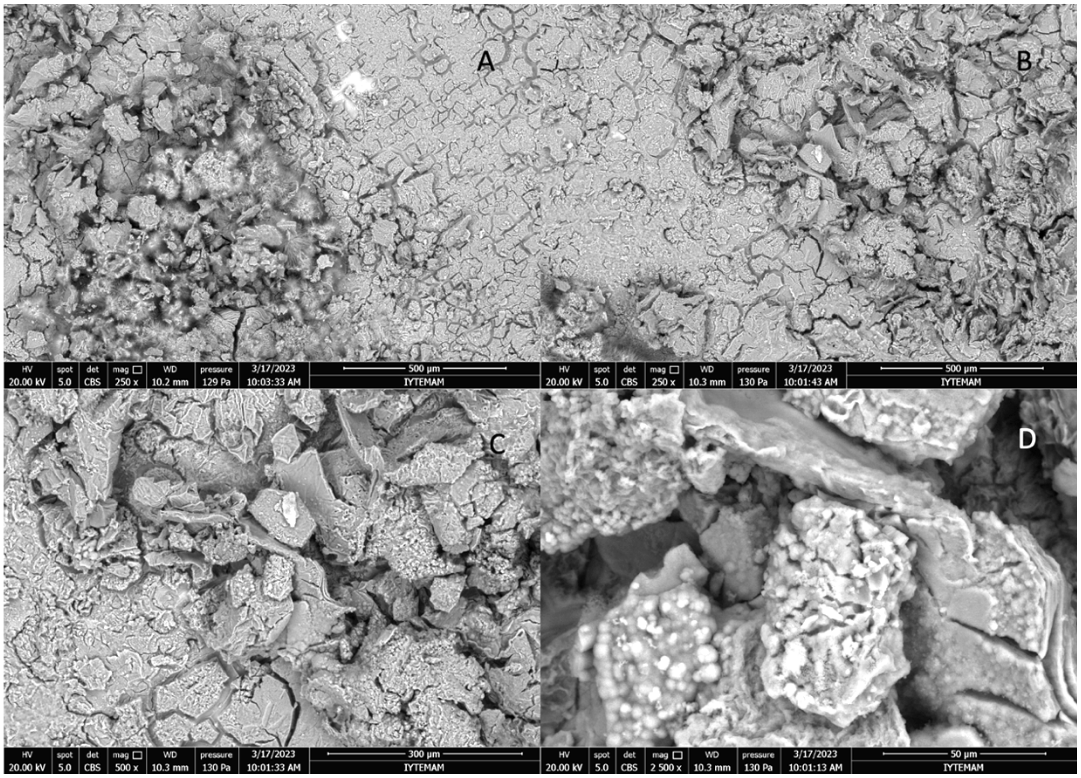

Figure S32. Surface morphologies of HTC samples after 14 day immersion in 3.5% NaCl solution prior to the corrosion test: wide view (A, B), close view of the same region (C, D).

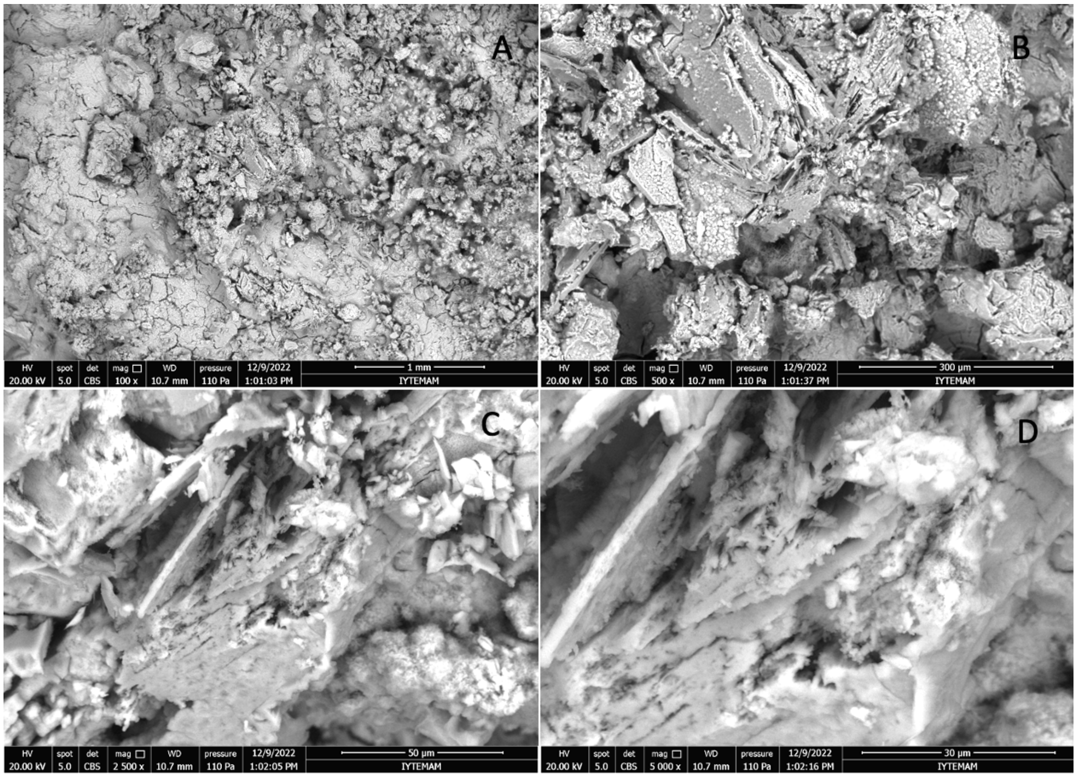

Figure S33. Surface morphologies of HTC samples after 21 day immersion in 3.5% NaCl solution prior to the corrosion test: wide view (A, B), close view of the same region (C, D).

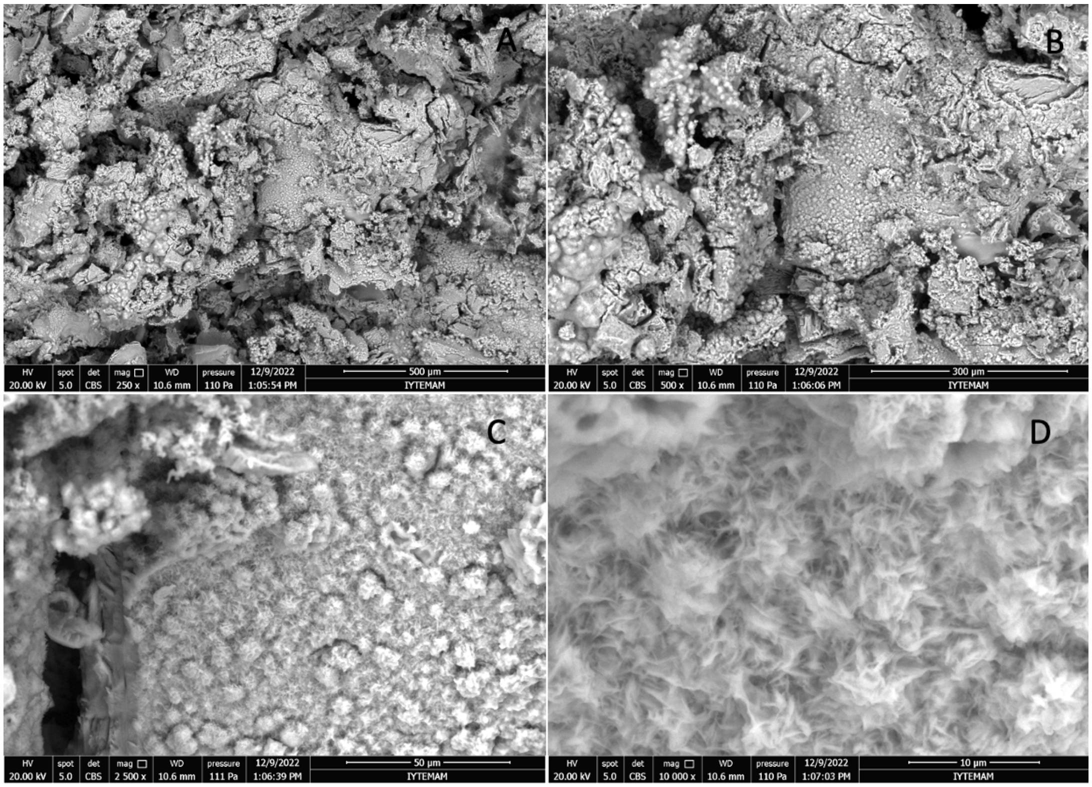

Figure S34. Surface morphologies of HTC samples after 21 day immersion in 3.5% NaCl solution prior to the corrosion test: wide view (A, B), close view of the same (C, D).

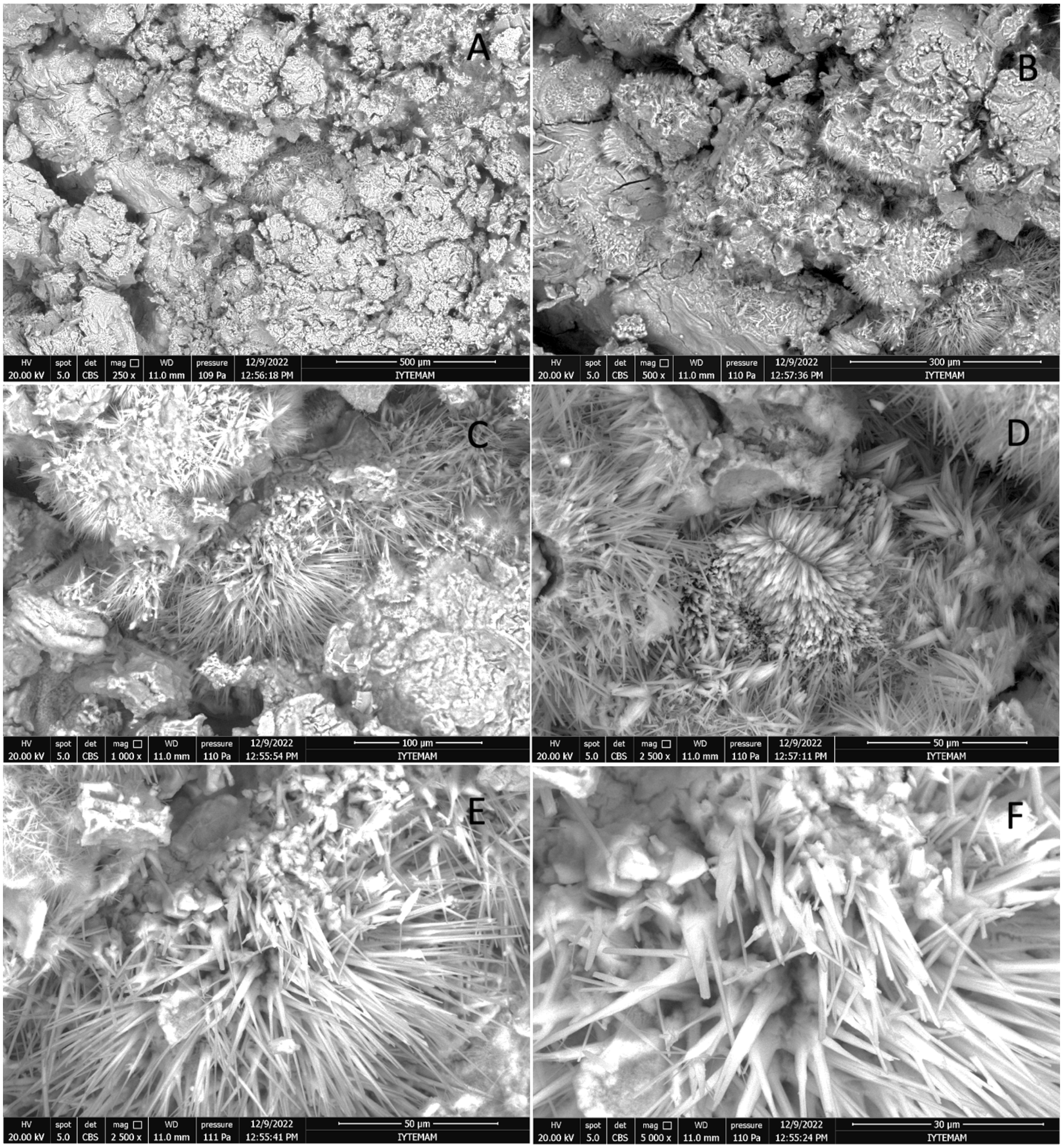

Figure S35. Surface morphologies of RTC samples after 21 day immersion in 3.5% NaCl solution prior to the corrosion test: wide view (A, B), close view of the same region (C, D), close views of needle-like formations in C (E, F).

Table S19. Surface elemental analysis of samples immersed in 3.5% NaCl solutions for various periods.

Surface morphology	EDX spectroscopy (weight and atomic ratio)
RTC-Initial<br>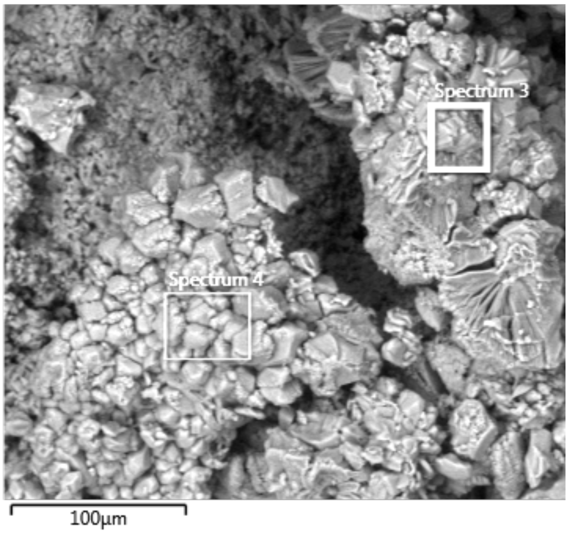	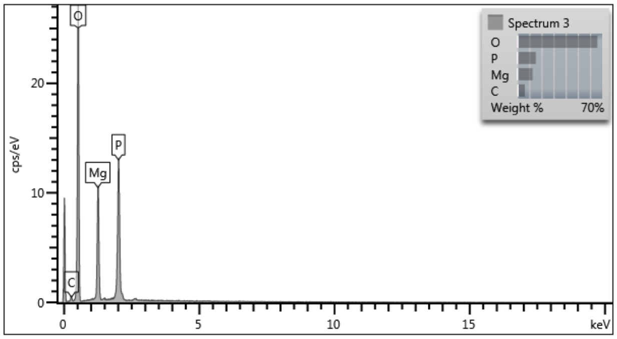<br>Mg/P/O/C= 9/9/73/9<br>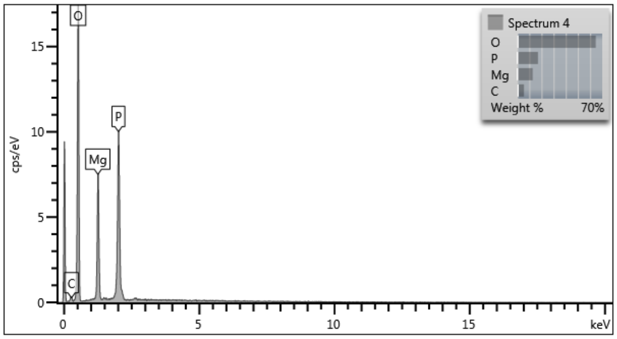<br>Mg/P/O/C=9/10/72/8
HTC-Initial<br>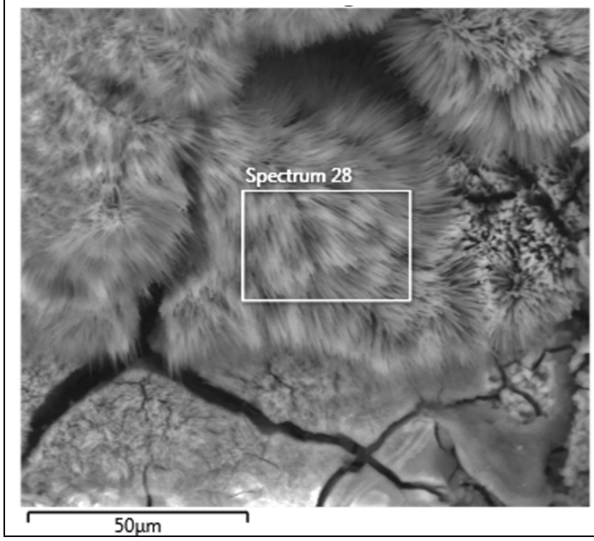	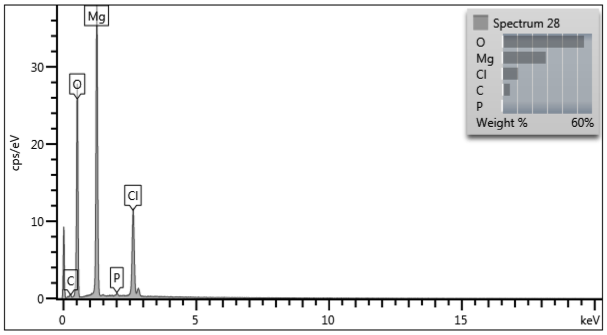<br>Mg/P/O/Cl= 22/0/64/6

HTC-Initial

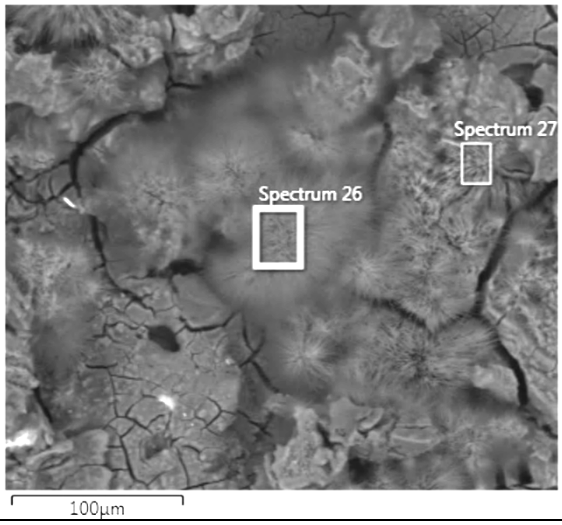

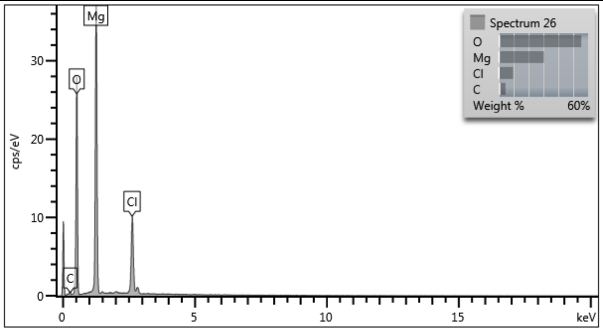

Mg/P/O/Cl= 23/0/65/5

RTC-Day1

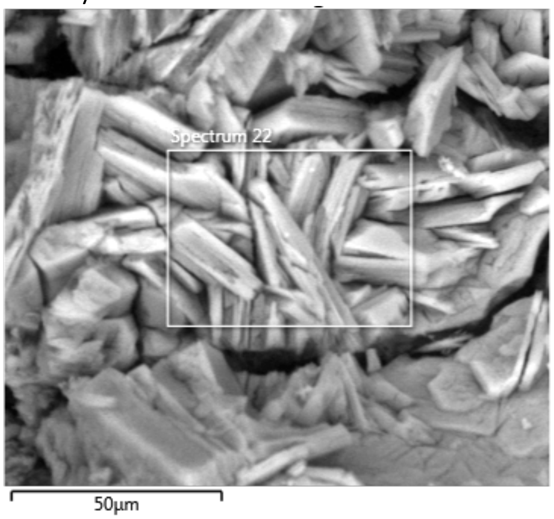

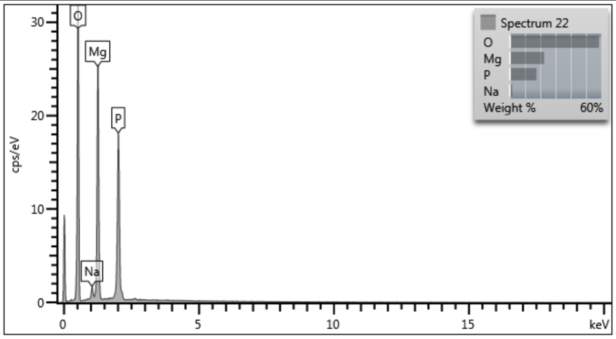

Mg/P/O/Na= 18/11/70/1

HTC-Day1

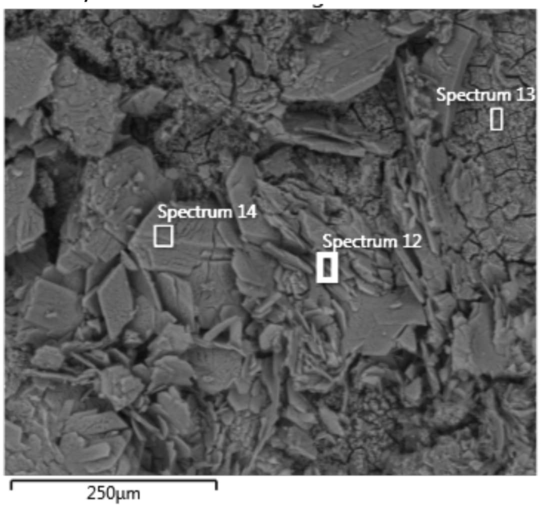

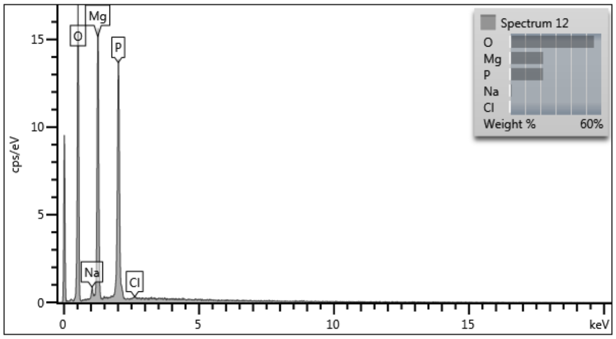

Mg/P/O=18/14/68

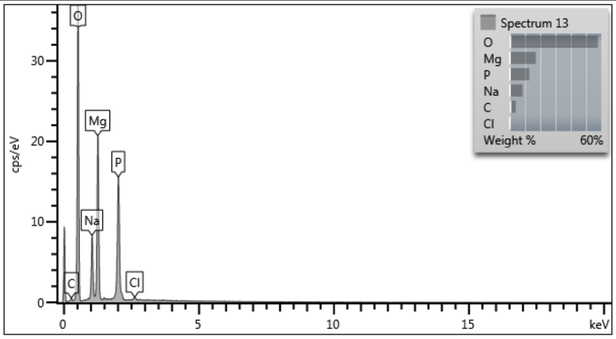

Mg/P/O/Na/C= 13/8/67/7/6

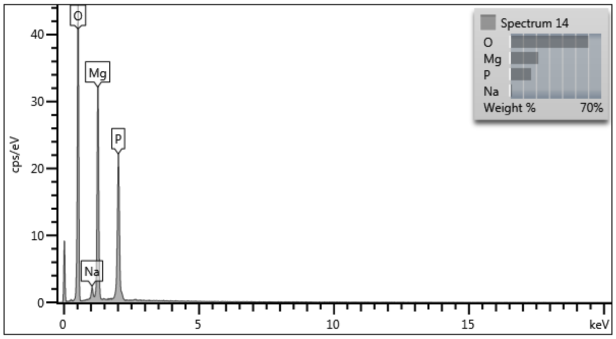

Mg/P/O= 17/10/72

RTC-Day1

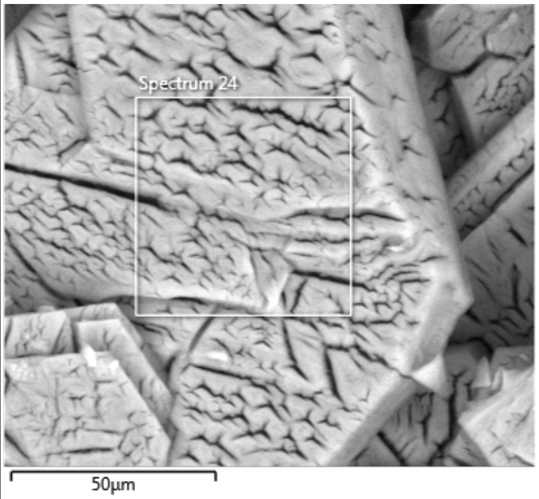

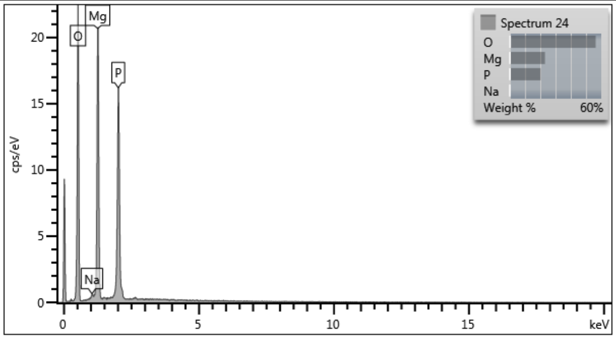

Mg/P/O= 18/13/69

RTC-Day3

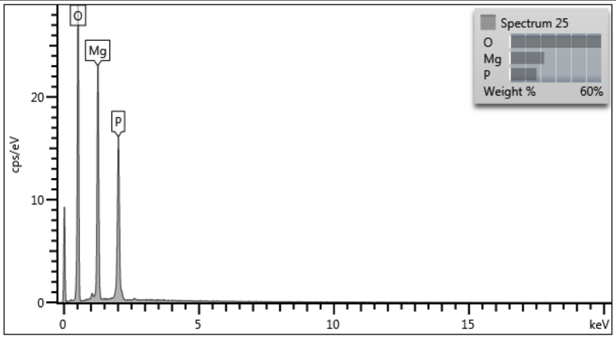

Mg/P/O= 18/11/71

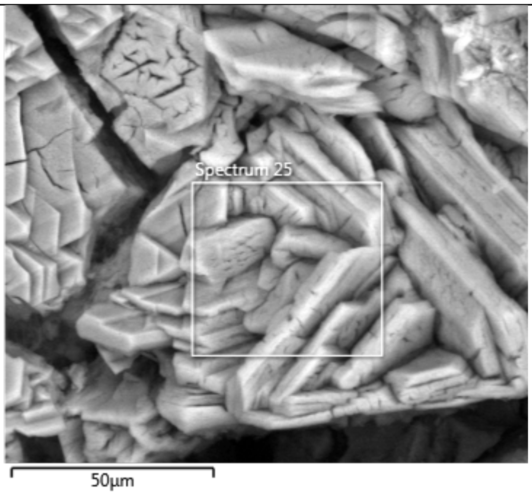	
HTC-Day3<br>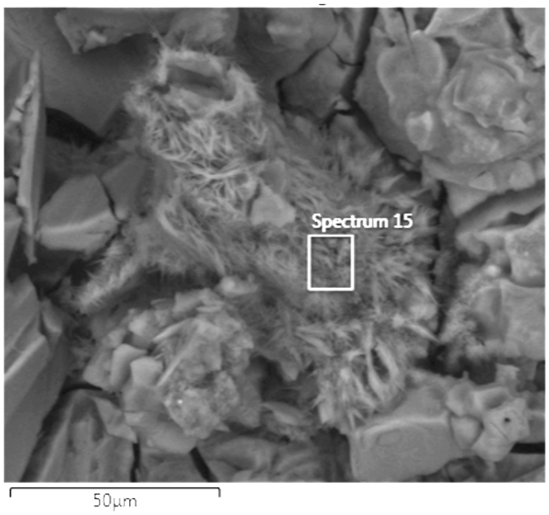	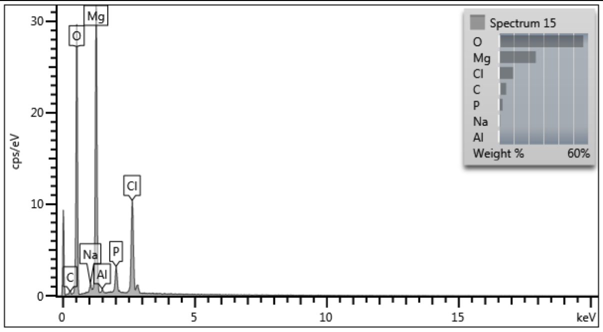<br>Mg/P/O/Cl= 19/2/66/5
HTC-Day3<br>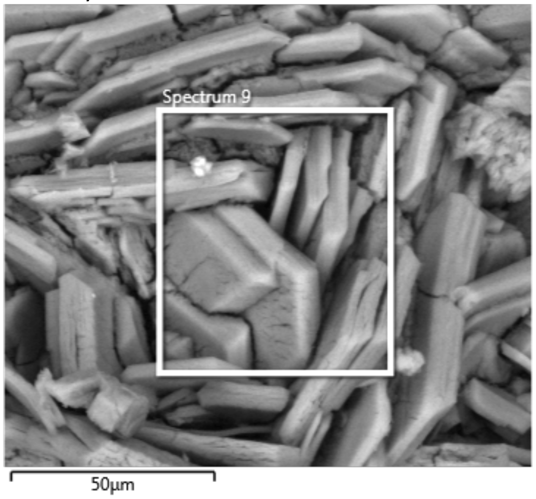	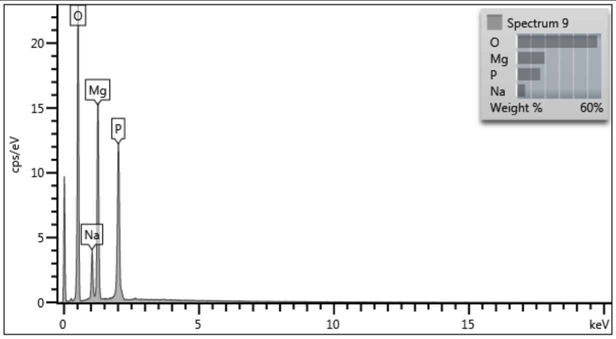<br>Mg/P/O/Na= 16/10/69/5

HTC-Day3

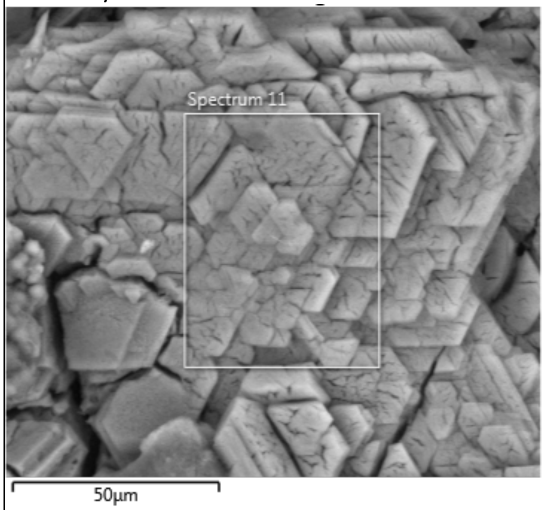

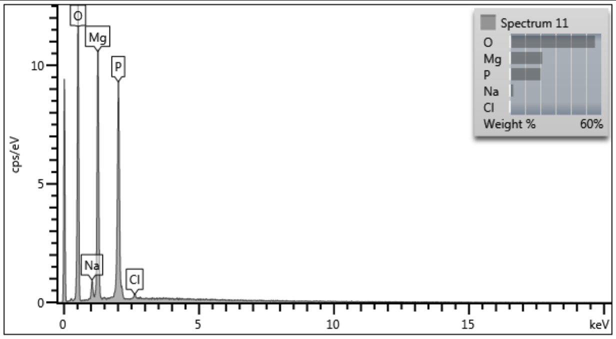

Mg/P/O/Na= 17/13/68/2

HTC-Day7

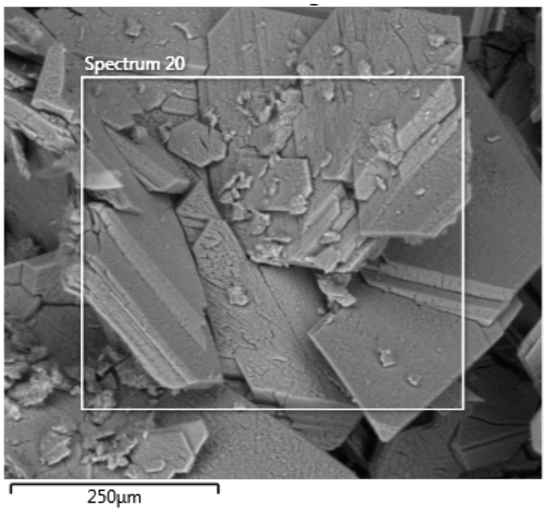

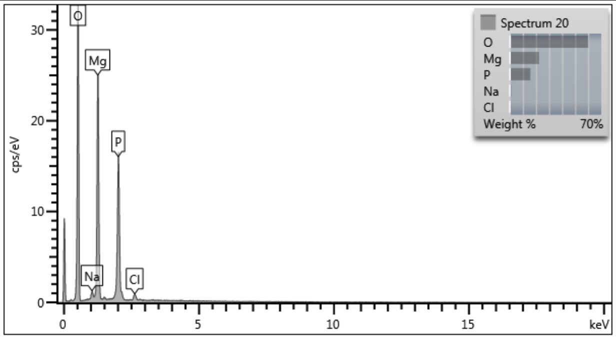

Mg/P/O= 17/10/72

HTC-Day7

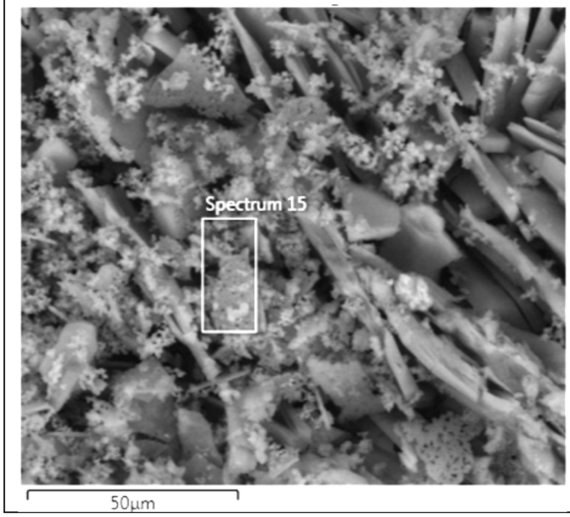

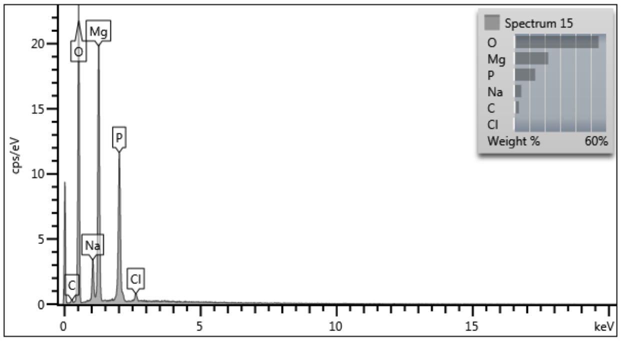

Mg/P/O= 17/8/65

HTC- Day7

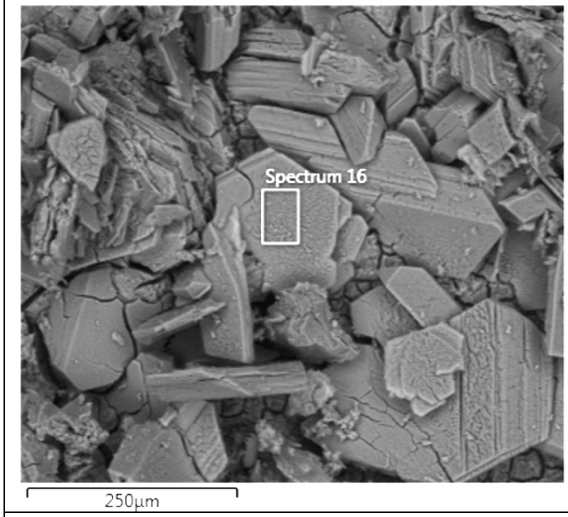

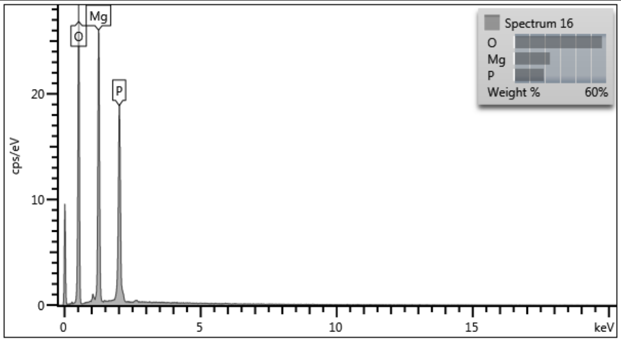

Mg/P/O= 19/12/69

RTC-Day14

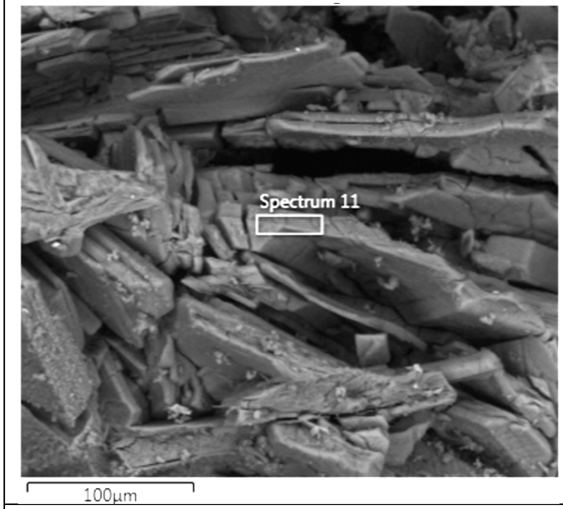

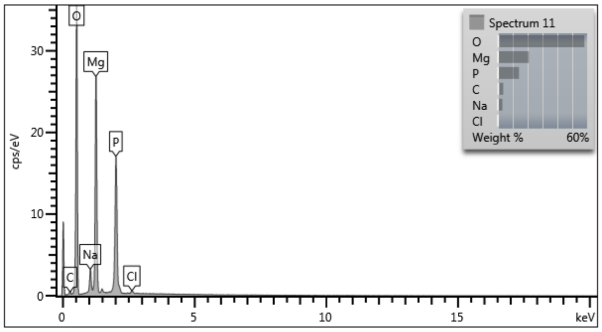

Mg/P/O= 16/8/68

HTC-Day14

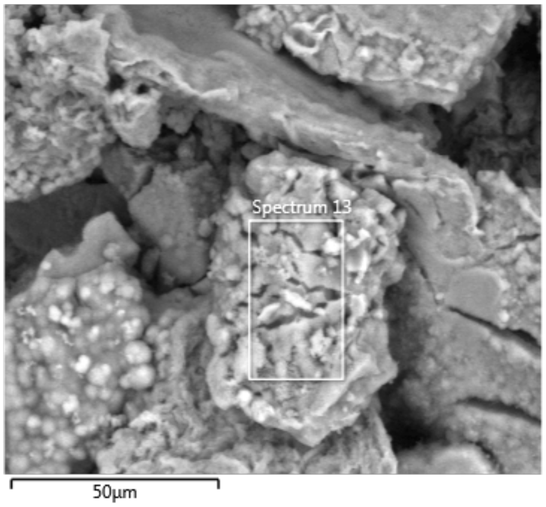

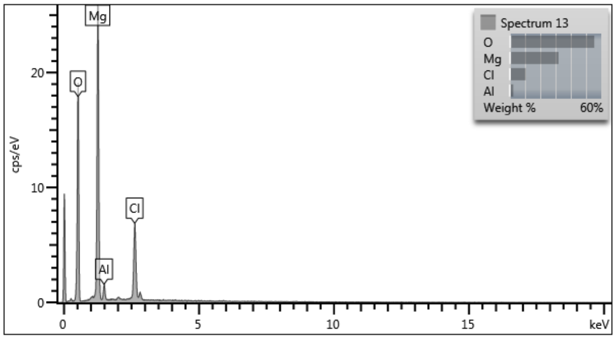

Mg/P/O/Cl=26/0/67/6

HTC-Day14

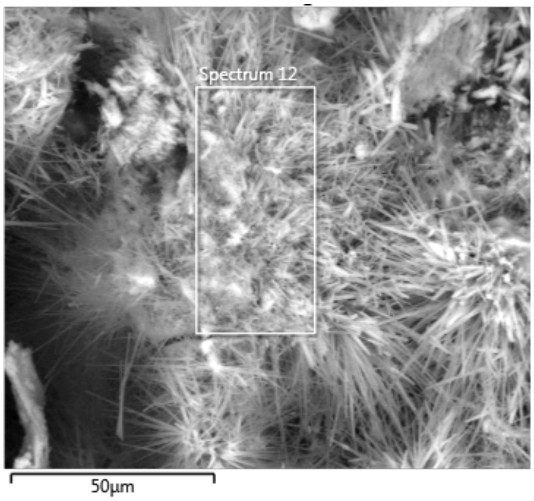

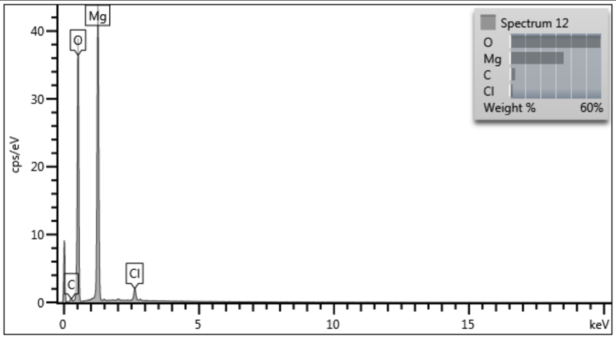

Mg/P/O/Cl/C= 26/0/67/1/5

RTC-Day21

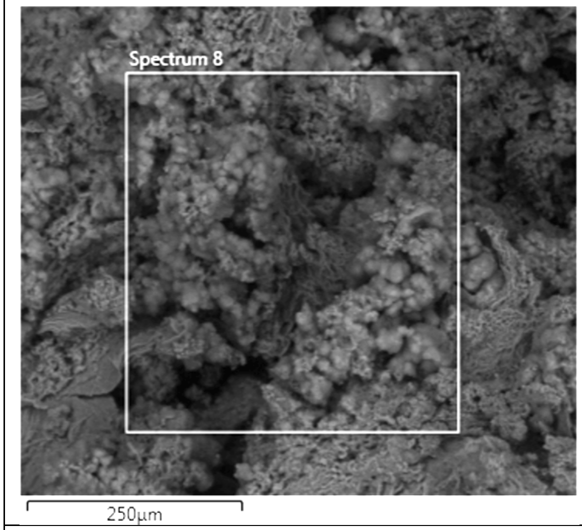

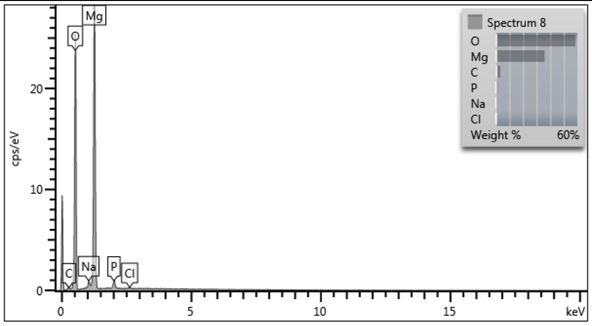

Mg/P/O/C= 27/1/67/5

RTC-Day21

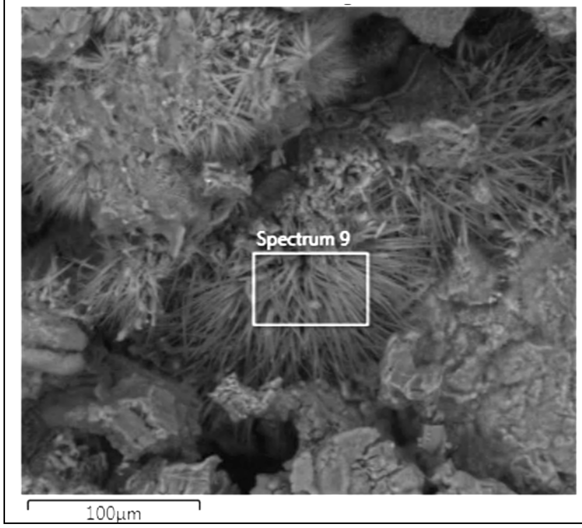

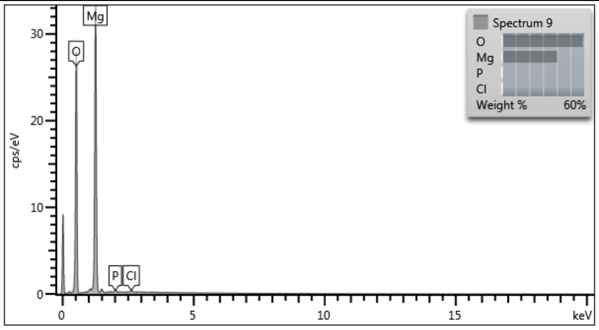

Mg/P/O= 31/0/69

RTC-Day21

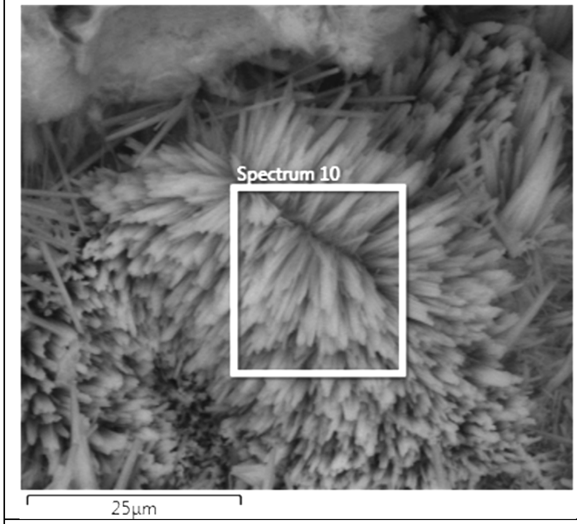

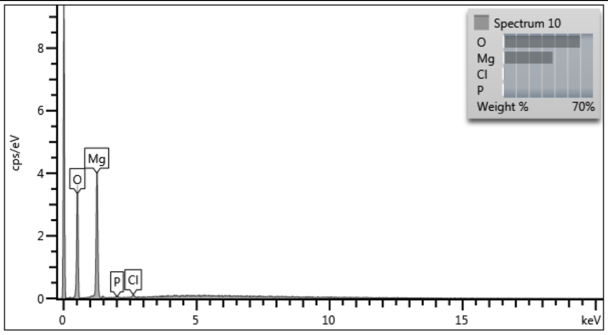

Mg/P/O/Cl= 29/0/70/0

HTC-Day21

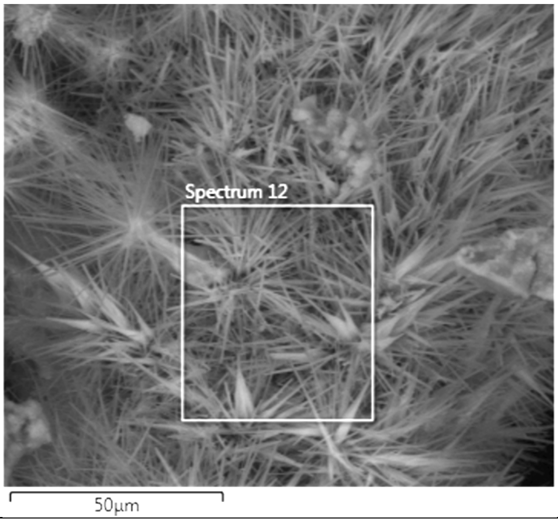

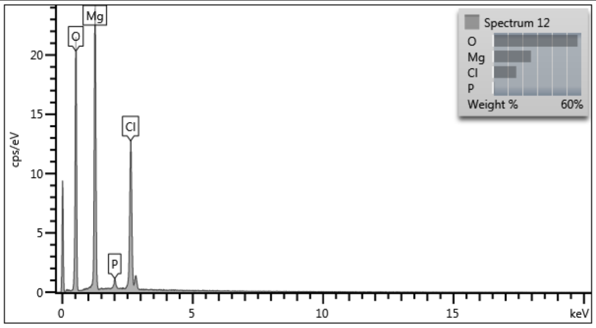

Mg/P/O/Cl= 21/1/70/9

HTC-Day21

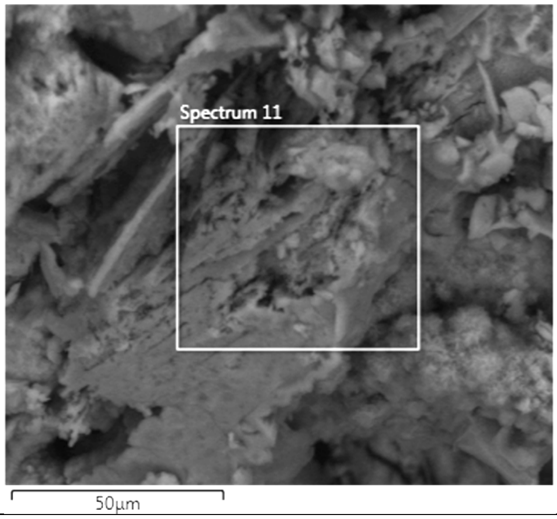

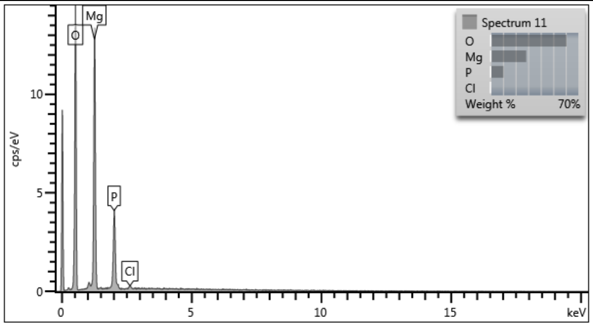

Mg/P/O= 22/6/71

HTC-Day21

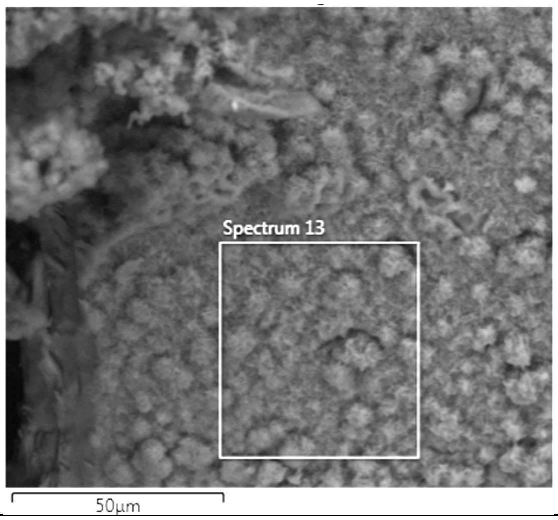

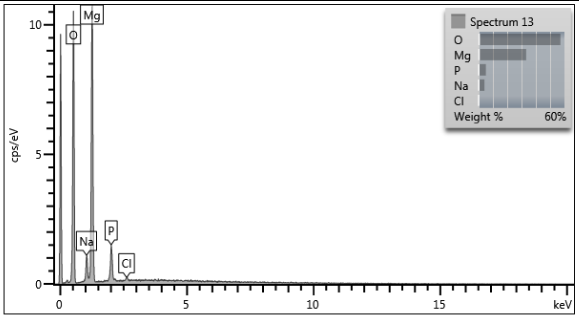

Mg/P/O/Na= 26/3/67/3
